# Working So Hard: Double‐Stranded RNAs That Can Perform Both Gene Activation and Gene Silencing

**DOI:** 10.1002/cbic.70434

**Published:** 2026-06-30

**Authors:** Virginia Wing‐Nam Chiu, Matthew Lawrence Hammill, Marwah Elabed, Sierra Varley, Yulia Lomonosova, Joseph Hoare, Jon Voutila, Henrik Hansen, Troels Koch, Jean‐Paul Desaulniers

**Affiliations:** ^1^ Faculty of Science Ontario Tech University Oshawa Canada; ^2^ MiNA Therapeutics London UK

**Keywords:** chemical modification, oligonucleotides, RNA, RNA activation, RNA interference, saRNA, siRNA

## Abstract

Double‐stranded RNAs (dsRNAs) are powerful tools with therapeutic potential because they can upregulate or downregulate proteins of interest within biological systems. Small activating RNAs (saRNAs) are a type of dsRNA, identical in structure and composition to small interfering RNAs (siRNAs). We previously observed that RNA duplexes with modifications such as 2′‐*O*‐methyl (2′‐*O*‐Me), 2′‐fluoro (2′‐F), locked (LNA), and unlocked nucleic acids (UNA), and a C_3_‐propyl spacer linker affect gene upregulation potency, where some of these chemical modifications are commonly used in siRNA design. In this study, we tested whether some of these same chemically modified saRNAs, along with new duplex variants, could also modulate gene silencing of the same target gene, *STING*, using an endogenous plasmid system. We demonstrate that saRNAs, shown to induce gene expression via RNA activation (RNAa), can also perform gene silencing as siRNAs. We show that the type and number of chemical modifications on the guide strand have strong influence on siRNA and saRNA activities, but do not identify any that specifically bias one over the other. In exploring the effects of chemical modifications on short RNAs, our long‐term goal aims to reveal how specific chemical modifications influence the RNA interference (RNAi) or RNAa pathway.

## Graphical abstract

Working so hard: *STING* saRNAs are capable of not only entering the nucleus to induce subsequent target gene upregulation, but can also act as an siRNA to perform gene silencing. Chemical modifications in saRNA guide and passenger strands affect the strength of gene activation or gene silencing activity.

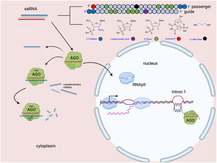

## 1 Introduction

Small activating RNAs (saRNAs) are double‐stranded RNAs that activate nuclear gene transcription via binding to regulatory elements near the DNA promoter region. Because aberrant gene expression is a hallmark of disease, being able to upregulate specific proteins offers a potential way to target diseases historically considered untreatable and undruggable [1, 2, 3, 4]. While there are no United States Food and Drug Administration‐approved saRNAs to date, there are saRNAs in clinical trials, demonstrating their relevance and upcoming potential [2, 5, 6, 7, 8]. Furthermore, saRNAs could also see useful benefit as a tool for biotechnology, where one could artificially upregulate specific gene(s) to answer questions of biological importance [4, 9, 10].

We recently reported that chemically modified saRNAs, consisting of 2′‐fluoro (2′‐F) adenosine, 2′‐*O*‐Me nucleic acids, locked nucleic acids (LNA), and a C_3_‐propyl linker, could induce high levels of gene activation to the gene target *STING* (stimulator of interferon genes), depending on the pattern and location of these specific modifications [11]. It is known that saRNAs utilize many common components (i.e., Argonaute 2 (AGO2)) to the well‐known gene silencing RNAs, short interfering RNAs (siRNAs). Among the four mammalian Argonautes (AGO1–4), AGO2 is unique in possessing endonucleolytic “slicer” activity, enabling direct cleavage of target RNAs to control gene expression. All AGO proteins function primarily as RNA‐guided regulatory scaffolds that mediate translational repression, RNA destabilization, and chromatin‐associated regulation through the recruitment of effector complexes [12, 13, 14, 15]. However, unlike siRNAs, the method of action of an saRNA is often dependent on context and design. For saRNAs, its target could be an antisense RNA or a promoter nascent RNA [16, 17]. For siRNAs, the target is solely the complementary target mRNA after proper RNA‐induced silencing complex (RISC) loading [14, 18].

While siRNAs downregulate gene expression, saRNAs induce upregulation by RNA activation (RNAa) [19, 20]. In 2006, Li and colleagues were the first to report the targeting of exogenously synthesized double‐stranded RNAs to the promoter region, triggering transcription—showing that exogenous dsRNAs could participate in RNA activation [9]. It has been observed that saRNAs are loaded onto AGO, as with siRNA. The saRNA–AGO complex translocates to the nucleus and forms a multiprotein complex [12, 13, 14, 21, 22]. Since siRNA and saRNA duplexes are compositionally identical and there are shared components between both activation and silencing mechanisms, it made us wonder if there is a connection between the activities from these RNA‐based molecules. While most current literature explores both saRNA and siRNA separately, there is little known regarding what factors control the relationship between siRNA and saRNA activities.

To overcome common obstacles of oligonucleotide therapeutics such as nuclease stability and off‐target effects, both saRNA and siRNA duplexes should be chemically modified [4, 23, 24, 25]. Combining the common siRNA chemical modifications such as 2′‐F and 2′‐*O*‐Me in combination with less studied siRNA modifications such as LNA and spacer linkages were implemented into the design of a library of chemically modified saRNAs to improve the upregulation of the target gene, *STING* [11, 26, 27, 28, 29]. *STING* plays an integral role in the immune response, responsible for signaling T‐cell priming and activation, and sensing cytosolic pathogens—making it a key target of interest for therapeutic purposes [30, 31, 32, 33, 34, 35, 36].

In that study, we observed that an RNA duplex modified with 2′‐F adenosines in both the passenger and guide strands, a single three‐carbon abasic C_3_‐propyl linker in the central position of the passenger strand, and a single LNA in the eighth position from the 5′‐end of the guide strand, resulted in a high level of upregulation (expression fold change relative quantification (RQ) of 9.6) [11]. In comparing the high performing saRNAs to those with poor gene upregulation, we observed that melting temperature (*T*_*m*_) played a crucial role in the saRNA's ability to drive gene upregulation. We observed that saRNAs with *T*_*m*_s within the 65°C–72°C range generally had higher RQs, affected and modulated by the chemical modifications present [11].

In this study, we further explore the effects that specific chemical modification patterns have on an saRNA's ability to perform gene upregulation and then test to see if the same saRNA duplexes are capable of acting like an siRNA to induce silencing. To the best of our knowledge, the dual role investigation that short RNAs can have for both gene activation and gene silencing has not yet been explored for this target. We believe this study is particularly important since unintended upregulation or silencing effects could be a source of off‐target effects for an saRNA—or for an siRNA. We report the ability of saRNAs to act like siRNAs that perform gene silencing. Additionally, we demonstrate that the presence of certain chemical modifications and their design composition can improve both the silencing activity and upregulation activity of a *STING* saRNA, but does not result in one gene expression pathway preferentially over the other. These findings are important for the oligonucleotide therapeutics field because they can inform and contextualize off‐target effects, which are important considerations for RNA duplex‐based drugs.

## 2 Materials and Methodology

### 2.1 RNA Synthesis and Purification

RNA passenger and guide strands were synthesized on an Applied Biosystems 394 DNA/RNA Synthesizer under argon gas at 55 psi, using 1.00 µmol solid supports, amidite reagents, and chemical modifications sourced from ChemGenes Corporation and Glen Research, used without further purification. Commercial phosphoramidites were dissolved to a 0.10 M concentration with anhydrous acetonitrile. Synthesis coupling times of all phosphoramidites were 600 s. To cleave strands from solid supports, 1.0 mL of EMAM (1:1 methylamine 40% wt. in H_2_O and methylamine in 33% wt. in ethanol) was exposed to the supports at full contact with the controlled pore glass for 30 min at room temperature. Oligonucleotides were then incubated with the EMAM solution at room temperature overnight. The next day, oligonucleotides were concentrated on a Centrovap evaporator and resuspended in a solution of 100 µL of filter sterilized DMSO and 125 µL of HF, and incubated for 3 h at 65°C for removal of 2′‐*O*‐TDBMS protecting groups. Oligonucleotides were concentrated again via Centrovap evaporator and precipitated over dry ice with 3 M NaOAc in EtOH. Millipore Amicon Ultra 3000 MW cellulose was used for final desalting.

Crude RNA yield was quantified using ThermoFisher GENESYS 10 UV–Vis spectrophotometer. RNA was purified using high‐performance liquid chromatography (HPLC) with a Waters 1525 binary HPLC pump with a Waters 2489 UV/Vis detector with a Avantor Vydac 218 MS 10 μm C18 150 × 22 mm 300 Å column with particle size 5 µm and confirmed purity with Avantor Vydac 218 MS 5 µm C18 150 × 4.6 mm 300 Å column with particle size 5 µm column. Over the course of 30 min, HPLC purification was performed using conditions that eluted from 5% to 100% acetonitrile in a triethylamine–acetic acid (0.1 M TEAA) buffer, at pH 7.00. Purified RNA was requantified by UV–Vis spectrophotometry. Purified RNAs were greater than 95% pure as analyzed by HPLC. Analytical HPLC spectra of newly tested strands can be found in the supporting information (SI) (Figures S24–S28).

Equimolar amounts of the desired guide and passenger strand for each duplex were suspended in a final volume of 200 µL of annealing buffer (75 mM KCl, 50 nM Tris‐Cl, 3 mM MgCl_2_, pH 6) in a 95°C water bath for 2 min to obtain a 20,000 nM duplex stock. Strands were left to anneal in the water bath until it was cooled to room temperature.

### 2.2 Thermal Denaturation and Circular Dichroism Studies

Duplex structure was verified and melting temperature was measured using Circular Dichroism (CD) Jasco J‐815 spectrophotometer. Equimolar amounts (10 µM) of each desired passenger and guide strand were annealed together in 900 µL of sodium phosphate buffer (10.0 mM Na_2_HPO_4_, 1.00 mM EDTA, 90.0 mM NaCl, pH 7.00) at 95°C in a water bath for 2 min and allowed to cool slowly to room temperature. The melting temperature calculated is the average of three runs and was obtained by the Meltwin v3.5 software, where temperature was increased to 95°C from 10°C at 0.5°C increase per minute. UV absorbance was measured at 260 nm. CD spectra were obtained in triplicates by scanning from 200 to 350 nm at 20.0 nm/min with a 0.20 nm data pitch at 25°C. Triplicates were averaged with the Jasco Spectra Manager v2 software.

### 2.3 Liquid Chromatography Quadrupole Time‐of‐Flight Mass Spectrometry

The molecular weight of each RNA strand was obtained from a liquid chromatography quadrupole time‐of‐flight mass spectrometer (Agilent 6545 QTOF‐MS), using an Agilent 1260 Infinity Binary HPLC Pump. To generate liquid chromatography–mass spectrometry chromatograms, the following parameters were used: 3200 *m*/*z* mass range, extended dynamic range 2 GHz, negative tuning mode, high‐resolution mode. An Agilent Poroshell 120 SB‐C18 2.1 × 100 mm 1.8 Micron Agilent column was used. Mobile phase gradient started at 95:5 of H_2_O:ACN, with a flow rate of 0.6 mL/min. Final injection volume was 20 μL, with 0.01 O.D/μL sample concentration. Chromatogram data were analyzed using the Agilent Technologies MassHunter Workstation Qualitative Analysis Software (Qual. 10.0). Mass spectrometry confirmation of newly tested strands is in the SI (Table S1).

### 2.4 Plasmid Design and Primer Site Mutagenesis

The target *STING* sequence, perfectly complementary to and targeted by the guide strand of the *STING* duplex, was cloned into a pGL3 plasmid at the 3′‐UTR of the luciferase gene via primer extension. Primers were designed to bind to the 3′‐UTR at the desired insertion site. The passenger *STING* DNA sequence was added to the 5′‐end of one primer, and the guide was added to the 5′‐end of another primer. All primers were ordered from Integrated DNA Technologies. The passenger strand primer sequence was 5′‐CGATTGGTTTCTCCACAACGGCCGCTTCGAGCAG‐3′, and the guide primer sequence was 3′‐GATCTCAGCCCCGCCGCTAACCAAAGAGGTGTTG‐5′. We note that the above‐mentioned perfect complement site is not present in annotated human mRNAs, making this silencing system an engineered surrogate readout for canonical RNAi activity. Such readout acts as a model to observe potential saRNA activity if the saRNA had a perfect complement in an endogenous mRNA transcript.

Polymerase chain reaction (PCR) was performed using PFU (Agilent) high fidelity polymerase, allowing the *STING* sequence on the primers to hybridize with their complementary sequences within the plasmid, thereby facilitating the integration of the *STING* sequence into the amplified DNA fragment. The PCR reaction was treated with DpnI to remove the methylated, original pGL3 template, and purified by PCR cleanup kit (Bio Basic). The sample was transformed into 25 μL of SIG10 chemically competent *E. coli* cells (Sigma‐Aldrich), through a heat shock at 40°C, and the cells were recovered in 500 μL of Super Optimal broth with Catabolite repression (SOC) broth for 1 h at 37°C. The cells were spun down to concentrate them and resuspended in 100 μL of Lysogeny Broth (LB). Fifty μL of cells was plated onto LB agar plates with 100 µg/mL of ampicillin (Sigma‐Aldrich) and incubated for 16 h at 37°C. Eight colonies that grew were selected and placed into 4 mL of LB broth and incubated at 4°C for 16 h. Plasmid DNA was extracted using a Bacterial Plasmid Purification kit (Bio Basic) and sent for sequencing (Genome Quebec) using primers that were designed to amplify a 1042‐base‐pair sequence, which would contain the *STING* sequence insertion site. Small insertion primers were designed to aid in the insertion of the *STING* sequence into pGL3 Luciferase reporter vectors by insertional site mutagenesis and one‐step PCR. *E. coli* was used to generate many copies by overnight bacterial culture incubation. The *STING* sequence's insertion was confirmed by PCR products and sequencing results. Plasmids were diluted and incubated overnight in sterile Falcon tubes with 5 mL LB broth and 5 µL ampicillin stock solution. Plasmid DNA from the *E.coli* was extracted using the Qiagen plasmid miniprep kit and the associated “Plasmid DNA Purification Using the QIAprep spin miniprep kit and a Microcentrifuge” protocol. The extracted pGL3 *STING* plasmids were quantified using the Genesys 10S UV–Vis spectrophotometer using absorbance readings at 260 nm. Plasmid map (Figures S30 and S31) and plasmid sequencing results for forward and reverse insertion primer alignments (Figure S32) can be found in the SI.

### 2.5 Procedure for HeLa Cell Transfection of saRNA With Lipofectamine 2000

On the day of cell passaging, HeLa cells (*Homo sapiens*, female, cervix, RRID: CVCL_0030 (ATCC CCL‐2)) were seeded into 12‐well plates that contained a final volume of 800 µL DMEM (10% fetal bovine serum (FBS), 1% penicillin–streptomycin) at with 100 µL of cell suspension from a 1.0 × 10^6^ cells/mL density per well. Plates were incubated with 5% CO_2_ at 37°C for 24 h. For each sample well, 1 µL of test saRNA, 2 µL of pGL3‐*STING* plasmid, 0.5 µL pRL‐SV40 plasmid, and 100 µL Gibco opti‐MEM were combined in a microcentrifuge tube on ice. In a second tube, 1 µL Lipofectamine 2000 and 100 µL Gibco opti‐MEM were incubated together at room temperature for 5 min. Contents of both tubes were combined and incubated at room temperature for 20 min, then aliquoted into their respective sample wells for a transfection incubation period of 24 h with 5% CO_2_ at 37°C.

### 2.6 Procedure for A549 Cell Transfection of saRNA With Lipofectamine 2000

On the day of cell passaging, A549 cells (*Homo sapiens*, male, lung, RRID: CVCL_0023) were seeded into 24‐well plates that contained a final volume of 400 µL F‐12K (10% FBS, 1% penicillin–streptomycin) with 40 µL of a 1.0 × 10^6^ cells/mL cell suspension per well to obtain 40,000 cells per well. Plates were incubated with 5% CO_2_ at 37°C for 24 h. For each sample well, 0.6 µL of Lipofectamine 2000 and 25 µL of Gibco opti‐MEM were incubated in a microcentrifuge tube at room temperature for 5 min. These contents were combined with a second tube which had 85 µL of opti‐MEM and the volume required of test saRNA to obtain a final concentration of 10 nM in the well. This solution was incubated at room temperature for 15 min. The contents were added to the respective well and were incubated at 5% CO_2_ at 37°C for 72 h.

### 2.7 Silencing Assay—HeLa Cells

Standard instructions for the Promega dual‐luciferase assay kit were followed from previously published procedures [37, 38]. Luminescence data were obtained using a Bio‐Tek CYTATION 5 imaging reader. Lysates were loaded into a 96‐well opaque Costar plate in triplicates. Lar II and Stop & Glo substrates were added to the lysates sequentially for the measurement of enzyme activity via luminescence for both *firefly/Renilla* luciferase.

### 2.8 RNA Extraction and Reverse Transcription—A549 Cells Upregulation Monitoring

Qiagen RNeasy Mini kit was used to manually extract RNA from A549 cells, following QIAGEN's “Purification of Total RNA from Animal Cells Using Spin Technology” protocol, obtained from the RNeasy Mini Handbook. A MBI BioDrop machine (1.00 dilution factor, fact 40.00, 10 mm pathlength) was used to quantify lysates, which were each normalized with to UltraPure DNase/RNase‐free distilled water (Thermofisher) 40 ng/µL. With a BioRad T100 Thermocycler and the Qiagen QuantiTect Reverse Transcription kit, reverse transcription for each lysate was performed. Twelve µL aliquots of each lysate and 2 µL of wipeout were added to a PCR plate and incubated at 42°C for 2 min. One µL of reverse transcription reagent, 1 µL of primer mix, and 4 µL of reaction buffer (5×) were added to each lysate well, and the plate was incubated at 42°C for 30 min. The temperature was changed to 95°C for 3 min to halt reverse transcription. The complementary DNA (cDNA) products were diluted with RNAse‐free distilled water to obtain a final volume of 100 µL. cDNA was quantified with the Biodrop machine and subsequently diluted to 20 ng/µL before undergoing quantitative polymerase chain reaction (qPCR), or storage at −18°C.

### 2.9 Quantitative Polymerase Chain Reaction—A549 Cells Upregulation Monitoring

To prepare the mastermix for each gene primer, Qiagen QuantiTect Primer assays for GAPDH ((GeneGlobe ID—QT00079247—Catalog No.—249900), AHSA ((GeneGlobe ID—QT00040670—Catalog No.—249900), POLR2A ((GeneGlobe ID—QT00033264—Catalog No.—249900), TMEM173 (GeneGlobe ID—QT00055440—Catalog No.—249900), UltraPure DNase/RNase‐free distilled water (Thermofisher), and SsoFast EvaGreen Supermix (BioRad) were used. Each primer assay was resuspended in 1.10 mL TE buffer (0.50 M EDTA, 1.0 M Tris‐Cl, H_2_O, pH 7.00). One µL primer assay mix, 5 µL EvaGreen Supermix, and 10 µL of purified distilled water were mixed together for each sample well.

Three µL of each cDNA sample was pipetted into a qPCR plate for qPCR, in triplicates for each different gene primer assay. Fifteen µL of each respective master mix was mixed into its respective wells to yield a final volume of 18 µL per well. After Microseal “B” PCR Plate Sealing Film (BioRad) was used to seal it, the qPCR plate was centrifuged to bring all reagents to the bottom of the plate. A BioRad CFX Connect qPCR machine was used. The qPCR protocol with the parameters; 95°C for 5.00 min, 95°C for 15.00 s, and 60°C for 30 s for total 40 cycles was used. Upon the end of the 40th cycle, melt curve data were obtained by adjusting temperature from 65°C to 95°C in 0.50°C increments for 5.00 s.

## 3 Statistical Analysis

Cycle threshold (Ct) values from qPCR were subjected to expression fold change calculations, where the untreated control was set at RQ = 1 and GAPDH was used as the housekeeping gene. The RQ for each bioreplicate was consolidated into Prism 10.4.1 (GraphPad Software). The mean and standard deviation were calculated using the descriptive statistics function, from minimum three bioreplicates. Data were analyzed using the one‐way analysis of variance (ANOVA) function built into the GraphPad software to calculate statistical significance to the untreated control, where the number of asterisks (*) indicated: *p < 0.05, **p < 0.005, ****p < 0.0001.

For silencing, *firefly/Renilla* luminescence ratios were expressed as a percentage of *f* *irefly* protein luminescence relative to saRNA efficacy. These ratios were compared to untreated controls, set as 100% luciferase expression. Resulting values for each bioreplicate experiment at each concentration tested were compiled into Prism v10.4.1 (GraphPad Software), where concentrations were transformed to logarithms (base of 10). IC_50_ values and ±95% CI of Log of IC_50_ were calculated using the nonlinear regression model (log[inhibitor] vs. normalized response—variable slope), from minimum two bioreplicates. The descriptive statistics function was used to calculate mean percent luciferase expression and standard deviation at each respective concentration. From the results of the nonlinear regression, an extra sum‐of‐squares *F* test was performed, where in the null hypothesis (*H*_0_), all curves share the same IC_50_, and in the alternate hypothesis (*H*_1_), at least one curve has a different IC_50_ (*p *< 0.05). Gene silencing data were further analyzed using one‐way ANOVA to calculate the statistical significance (*p *= 0.05) of the mean percent relative luciferase expression at the highest tested concentration for each respective duplex to the untreated control (100% relative luciferase expression), where the number of asterisks (*) indicated: ****p < 0.0001.

## 4 Results

### 4.1 Thermal Stability and Circular Dichroism Studies

**FIGURE 1:**
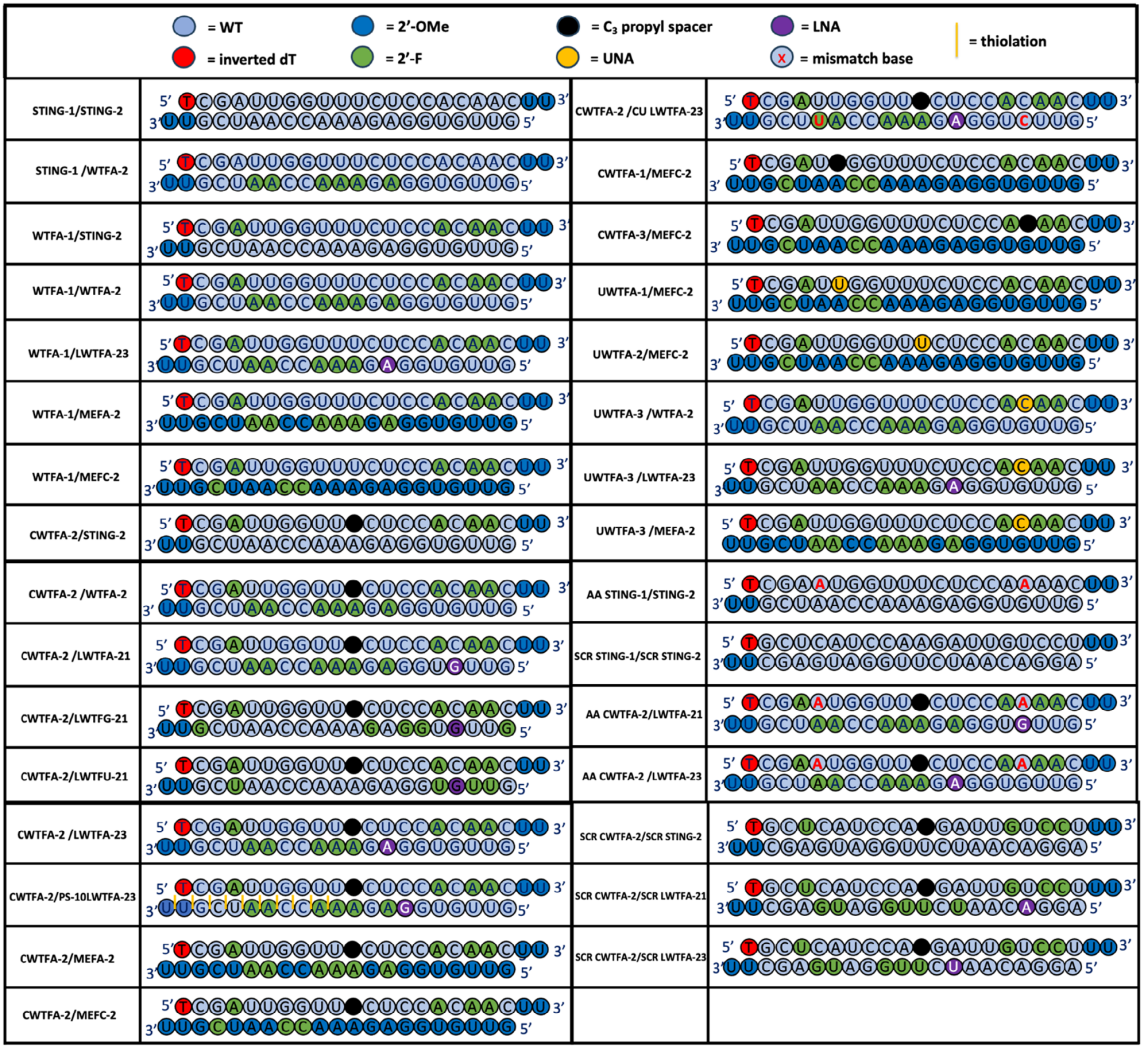
Chemical modification composition and placement of saRNA designs targeting *STING* gene. Bubbles are color coded to correspond to a type of chemical modification for visualization.

RNAs were annealed to their respective complementary strands using the methodology described above. The duplexes annealed for testing are shown in Table 1. Figure 1 is also generated in a colored “bubble” format to highlight the types and positions of the various chemical modifications. Table 2 highlights the melting temperatures of the duplexes. CD spectra were acquired to confirm the A‐form helical conformation of saRNA duplexes, where at 210 nm, negative CD bands and positive CD bands at 265 nm were observed. Generally, chemical modifications created differences in both amplitude and crossover points, but did not change the overall A‐form helical conformation shape. CD spectra and melting curves for each duplex can be found in the SI (Figures S1–S11) and (Figures S20–S23).

**TABLE 1:** Composition and placement of chemical modifications in RNA duplex designs, targeting the *STING* gene.^a^

Duplex	Sequence
STING‐1/STING‐2	5′‐TCGAUUGGUUUCUCCACAACU_M_U_M_‐3′ 3′‐U_M_U_M_GCUAACCAAAGAGGUGUUG‐5′
STING‐1/WTFA‐2	5′‐TCGAUUGGUUUCUCCACAACU_M_U_M_‐3′ 3′‐U_M_U_M_GCUA_F_A_F_CCA_F_A_F_A_F_GA_F_GGUGUUG‐5′
WTFA‐1/STING‐2	5′‐TCGA_F_UUGGUUUCUCCA_F_CA_F_A_F_CU_M_U_M_‐3′ 3′‐U_M_U_M_GCUAACCAAAGAGGUGUUG‐5
WTFA‐1/WTFA‐2	5′‐TCGA_F_UUGGUUUCUCCA_F_CA_F_A_F_CU_M_U_M_‐3′ 3′‐U_M_U_M_GCUA_F_A_F_CCA_F_A_F_A_F_GA_F_GGUGUUG‐5′
WTFA‐1/LWTFA‐23	5′‐TCGA_F_UUGGUUUCUCCA_F_CA_F_A_F_CU_M_U_M_‐3′ 3′‐U_M_U_M_GCUA_F_A_F_CCA_F_A_F_A_F_GA_L_GGUGUUG‐5′
WTFA‐1/MEFA‐2	5′‐TCGA_F_UUGGUUUCUCCA_F_CA_F_A_F_CU_M_U_M_‐3′ 3′‐U_M_U_M_G_M_C_M_U_M_A_F_A_F_C_M_C_M_A_F_A_F_A_F_G_M_A_F_G_M_G_M_U_M_G_M_U_M_U_M_G_M_‐5′
WTFA‐1/MEFC‐2	5′‐TCGA_F_UUGGUUUCUCCA_F_CA_F_A_F_CU_M_U_M_‐3′ 3′‐U_M_U_M_G_M_C_F_U_M_A_M_A_M_C_F_C_F_A_M_A_M_A_M_G_M_A_M_G_M_G_M_U_M_G_M_U_M_U_M_G_M_‐5′
CWTFA‐2/STING‐2	5′‐TCGA_F_UUGGUUC_3_UCCA_F_CA_F_A_F_CU_M_U_M_‐3′ 3′‐UUGCUAACCAAAGAGGUGUUG‐5′
CWTFA‐2/WTFA‐2	5′‐TCGA_F_UUGGUUC_3_UCCA_F_CA_F_A_F_CU_M_U_M_‐3′ 3′‐U_M_U_M_GCUA_F_A_F_CCA_F_A_F_A_F_GA_F_GGUGUUG‐5′
CWTFA‐2/LWTFA‐21	5′‐TCGA_F_UUGGUUC_3_UCCA_F_CA_F_A_F_CU_M_U_M_‐3′ 3′‐U_M_U_M_GCUA_F_A_F_CCA_F_A_F_A_F_GA_F_GGUG_L_UUG‐5′
CWTFA‐2/LWTFG‐21	5′‐TCGA_F_UUGGUUC_3_UCCA_F_CA_F_A_F_CU_M_U_M_‐3′ 3′‐U_M_U_M_G_F_CUAACCAAAG_F_AG_F_G_F_UG_L_UUG‐5′
CWTFA‐2/LWTFU‐21	5′‐TCGA_F_UUGGUUC_3_UCCA_F_CA_F_A_F_CU_M_U_M_‐3′ 3′‐U_M_U_M_GCU_F_AACCAAAGAGGUG_L_U_F_U_F_G‐5′
CWTFA‐2/LWTFA‐23	5′‐TCGA_F_UUGGUUC_3_UCCA_F_CA_F_A_F_CU_M_U_M_‐3′ 3′‐U_M_U_M_GCUA_F_A_F_CCA_F_A_F_A_F_GA_L_GGUGUUG‐5′
CWTFA‐2/PS‐10LWTFA‐23	5′‐TCGA_F_UUGGUUC_3_UCCA_F_CA_F_A_F_CU_M_U_M_‐3′ 3′‐U_M_*U_M_*G*C*U*A_F_*A_F_*C*C*A_F_*A_F_A_F_GA_F_G_L_GUGUUG‐5′
CWTFA‐2/MEFA‐2	5′‐TCGA_F_UUGGUUC_3_UCCA_F_CA_F_A_F_CU_M_U_M_‐3′ 3′‐U_M_U_M_G_M_C_M_U_M_A_F_A_F_C_M_C_M_A_F_A_F_A_F_G_M_A_F_G_M_G_M_U_M_G_M_U_M_U_M_G_M_‐5′
CWTFA‐2/MEFC‐2	5′‐TCGA_F_UUGGUUC_3_UCCA_F_CA_F_A_F_CU_M_U_M_‐3′ 3′‐U_M_U_M_G_M_C_F_U_M_A_M_A_M_C_F_C_F_A_M_A_M_A_M_G_M_A_M_G_M_G_M_U_M_G_M_U_M_U_M_G_M_‐5′
CWTFA‐2/CU LWTFA‐23	5′‐TCGA_F_UUGGUUC_3_UCCA_F_CA_F_A_F_CU_M_U_M_‐3′ 3′‐U_M_U_M_GCUU_F_A_F_CCA_F_A_F_A_F_GA_L_GGUCUUG‐5′
CWTFA‐1/MEFC‐2	5′‐TCGA_F_UC_3_GGUUUCUCCA_F_CA_F_A_F_CU_M_U_M_‐3′ 3′‐U_M_U_M_G_M_C_F_U_M_A_M_A_M_C_F_C_F_A_M_A_M_A_M_G_M_A_M_G_M_G_M_U_M_G_M_U_M_U_M_G_M_‐5′
CWTFA‐3/MEFC‐2	5′‐TCGA_F_UUGGUUUCUCCA_F_C_3_A_F_A_F_CU_M_U_M_‐3′ 3′‐U_M_U_M_G_M_C_F_U_M_A_M_A_M_C_F_C_F_A_M_A_M_A_M_G_M_A_M_G_M_G_M_U_M_G_M_U_M_U_M_G_M_‐5′
UWTFA‐1/MEFC‐2	5′‐TCGA_F_UU_U_GGUUUCUCCA_F_CA_F_A_F_CU_M_U_M_‐3′ 3′‐U_M_U_M_G_M_C_F_U_M_A_M_A_M_C_F_C_F_A_M_A_M_A_M_G_M_A_M_G_M_G_M_U_M_G_M_U_M_U_M_G_M_‐5′
UWTFA‐2/MEFC‐2	5′‐TCGA_F_UUGGUUU_U_CUCCA_F_CA_F_A_F_CU_M_U_M_‐3′ 3′‐U_M_U_M_G_M_C_F_U_M_A_M_A_M_C_F_C_F_A_M_A_M_A_M_G_M_A_M_G_M_G_M_U_M_G_M_U_M_U_M_G_M_‐5′
UWTFA‐3/WTFA‐2	5′‐TCGA_F_UUGGUUUCUCCAC_U_A_F_A_F_CU_M_U_M_‐3′ 3′‐U_M_U_M_GCUA_F_A_F_CCA_F_A_F_A_F_GA_F_GGUGUUG‐5′
UWTFA‐3/LWTFA‐23	5′‐TCGA_F_UUGGUUUCUCCAC_U_A_F_A_F_CU_M_U_M_‐3′ 3′‐U_M_U_M_GCUA_F_A_F_CCA_F_A_F_A_F_GA_L_GGUGUUG‐5′
UWTFA‐3/MEFA‐2	5′‐TCGA_F_UUGGUUUCUCCAC_U_A_F_A_F_CU_M_U_M_‐3′ 3′‐U_M_U_M_G_M_C_M_U_M_A_F_A_F_C_M_C_M_A_F_A_F_A_F_G_M_A_F_G_M_G_M_U_M_G_M_U_M_U_M_G_M_‐5′
AA STING‐1/STING‐2	5′‐TCGAAUGGUUUCUCCAAAACU_M_U_M_‐3′ 3′‐U_M_U_M_GCUAACCAAAGAGGUGUUG‐5′
SCR STING‐1/SCR STING‐2	5′‐TGCUCAUCCAAGAUUGUCCUU_M_U_M_‐3′ 3′‐U_M_U_M_CGAGUAGGUUCUAACAGGA‐5′
AA CWTFA‐2/LWTFA‐21	5′‐TCGA_F_ AUGGUUC_3_CUCCA_F_ AA_F_A_F_CU_M_U_M_‐3′ 3′‐U_M_U_M_GCUA_F_A_F_CCA_F_A_F_A_F_GA_F_GGUG_L_UUG‐5′
AA CWTFA‐2/LWTFA‐23	5′‐TCGA_F_ AUGGUUC_3_CUCCA_F_ AA_F_A_F_CU_M_U_M_‐3′ 3′‐U_M_U_M_GCUA_F_A_F_CCA_F_A_F_A_F_GA_L_GGUGUUG‐5′
SCR CWTFA‐2/SCR STING‐2	5′‐TGCUCAUCCAC_3_GAUUGUCCUU_M_U_M_‐3′ 3′‐U_M_U_M_CGAGUAGGUUCUAACAGGA‐5′
SCR CWTFA‐2/SCR LWTFA‐21	5′‐TGCUCAUCCAC_3_GAUUGUCCUU_M_U_M_‐3′ 3′‐U_M_U_M_CGAG_F_U_F_AGG_F_U_F_U_F_CU_F_AACA_L_GGA‐5′
SCR CWTFA‐2/SCR LWTFA‐23	5′‐TGCUCAUCCAC_3_GAUUGUCCUU_M_U_M_‐3′ 3′‐U_M_U_M_CGAG_F_U_F_AGG_F_U_F_U_F_CU_L_AACAGGA‐5′

a Subscript indicates chemical modifications to the monomer: *M* = 2′‐*O*‐Me, *F* = 2′‐fluoro, *L* = LNA, *U* = UNA *C*_#_ = abasic carbon spacer with subscript index indicating the number of carbon atoms, * = phosphorothioate linkage, *T* = inverted dT, *X* = mismatch. The top strand corresponds to the passenger strand; the bottom strand corresponds to the guide strand.

**TABLE 2:** Upregulation data, silencing data, IC_50_, ±95% CI of log IC_50_, and melting temperature of saRNA duplexes.^a^

Legend Upregulation (RQ): Inactive = activity < 1 (control) Moderate = 1 ≤ activity ≤ 2 Strong = activity > 2				Silencing (IC_50_): NS = no silencing Inactive = *x* > 3000 pM Moderate = 1501 < *x* < 3000 pM Strong = 1500 pM > *x*				
RNA passenger strand	RNA guide strand	Upregulation (RQ)	% Upregulation	% Silencing	Silencing power	IC_50_, pM	±95% CI of Log IC_50_	Melting temperature, °C
STING‐1	STING‐2	4.1	410	94.6	Strong	1.5	−0.7 to 0.8	68
STING‐1	WTFA‐2	5.4	540	53.9	Moderate	2720	3.1 to 4.1	73
WTFA‐1	STING‐2	3.6	360	62.7	Moderate	1783	2.9 to 3.8	77
WTFA‐1	WTFA‐2	8.1	810	91.4	Strong	55.8	1.6 to 1.9	73
WTFA‐1	LWTFA‐23	4.0	400	80.4	Strong	317.3	2.3 to 2.8	79
WTFA‐1	MEFA‐2	1.7	170	27.2	NS	NA	4.6 to 6.1	81
WTFA‐1	MEFC‐2	1.1	110	36.2	NS	NA	NA	82
CWTFA‐2	STING‐2	4.1	410	73.3	Strong	786.4	2.6 to 3.3	52
CWTFA‐2	WTFA‐2	6.2	620	70.6	Strong	330.3	2.3 to 2.7	64
CWTFA‐2	LWTFA‐21	9.0	900	64.6	Strong	385.4	2.3 to 2.9	72
CWTFA‐2	LWTFG‐21	6.2	620	84.3	Strong	167.2	2.1 to 2.4	68
CWTFA‐2	LWTFU‐21	4.2	420	78.7	Strong	143.5	1.9 to 2.4	69
CWTFA‐2	LWTFA‐23	9.6	960	77	Strong	197.3	2.0 to 2.6	68
CWTFA‐2	PS‐10‐LWTFA‐23	7.4	740	78.1	Strong	171.8	2.0 to 2.5	68
CWTFA‐2	MEFA‐2	1.0	100	4.1	NS	NA	NA	70
CWTFA‐2	MEFC‐2	0.9	90	25	NS	NA	3.9 to 9.2	73
CWTFA‐2	CU LWTFA‐23	0.8	80	0.1	NS	NA	NA	57
CWTFA‐1	MEFC‐2	1.0	100	0	NS	NA	NA	75
CWTFA‐3	MEFC‐2	1.2	120	7.9	NS	NA	NA	67
UWTFA‐1	MEFC‐2	1.0	100	9.9	NS	NA	NA	64
UWTFA‐2	MEFC‐2	0.9	90	1.9	NS	NA	NA	71
UWTFA‐3	WTFA‐2	2.1	210	67.4	Strong	497.6	2.2 to 3.3	70
UWTFA‐3	LWTFA‐23	1.7	170	80	Strong	58.5	1.4 to 2.1	73
UWTFA‐3	MEFA‐2	0.9	90	25.4	NS	NA	NA	73
AA STING‐1	STING‐2	1.2	120	30.8	Poor	NA	NA	56
SCR STING‐1	SCR STING‐2	1.3	130	0	NS	NA	NA	74
AA CWTFA‐2	LWTFA‐21	1.6	160	18.3	Poor	NA	NA	55
AA CWTFA‐2	LWTFA‐23	1.6	160	46.1	Poor	7943	NA	55
SCR CWTFA‐2	SCR STING‐2	1.3	130	8.8	NS	NA	NA	52
SCR CWTFA‐2	SCR LWTFA‐21	1.0	100	30.1	NS	NA	NA	61
SCR CWTFA‐2	SCR LWTFA‐23	0.8	80	2.2	NS	NA	NA	52

a NS indicates no silencing activity. NA indicates non‐applicable. IC_50_ values greater than 3000 pM are categorized as poor silencing activity. IC_50_ values between 1501 pM and 3000 pM are categorized as moderate silencing. IC_50_ values less than 1500 pM are categorized as strong silencing. Expression fold change (RQ) values less than 1.2 are categorized as poor upregulation. RQ values between 1.2 and 2.0 are categorized as moderate upregulation. RQ values greater than 2.0 are categorized as strong upregulation. RQ values of some saRNA duplexes are taken from data obtained in a previous publication but included in these figures for ease of comparison (11). Percent (%) upregulation indicates the RQ as a percentage. Percent (%) silencing indicates the percentage of silencing at the highest concentration (pM) each respective saRNA was tested at.

From our previous study, we observed that melting temperature (*T*_*m*_) increased with increasing amounts of LNA and 2′‐F units within the duplexes (11). In this study, we observed that duplexes with a completely modified guide strand, composed entirely of 2′‐*O*‐Me and 2′‐F modifications, had the highest melting temperatures (WTFA‐1/MEFA‐2, *T*_*m*_ = 82°C) in comparison to their corresponding duplexes containing a less modified guide strand (ex. WTFA‐1/WTFA‐2, *T*_*m*_ = 73°C). These duplexes included: CWTFA‐2/MEFA‐2, CWTFA‐1/MEFC‐2, CWTFA‐2/MEFC‐2, CWTFA‐2/MEFC‐2, UWTFA‐1/MEFC‐2, UWTFA‐2/MEFC‐2, UWTFA‐3/MEFA‐2, and WTFA‐1/MEFA‐2, with *T*_*m*_s ranging between 64°C and 82°C. We anticipated that these highly modified guide strand designs, when paired with its passenger strand complement, would have high thermal stability, given that 2′‐*O*‐Me and 2′‐fluoro modifications—and LNA in particular—are known to increase both thermal stability, stabilizing of right‐handed A‐form helical conformation and maintenance of the C3′‐endo ribose sugar pucker [39]. We aimed to optimize the use of stabilizing modifications, trying to keep a balance that allowed us to fall within the optimal melting temperature range we observed in our previous study [11].

### 4.2 Gene Activation Studies

Previously, we demonstrated that different combinations of chemical modifications on a *STING* saRNA could titrate the magnitude of *STING* mRNA upregulation [11]. In this saRNA, the guide (active) strand was in the 3′–5′ orientation and an inverted dT was added to the 5′‐terminus of the passenger (inactive) strand to inhibit AGO loading. Chemical blocking of the 5′‐phosphate on an siRNA or saRNA strand has been shown to inhibit AGO loading, as the 5′‐monophosphate is required for efficient recognition and incorporation into the RISC complex [40, 41].

**FIGURE 2:**
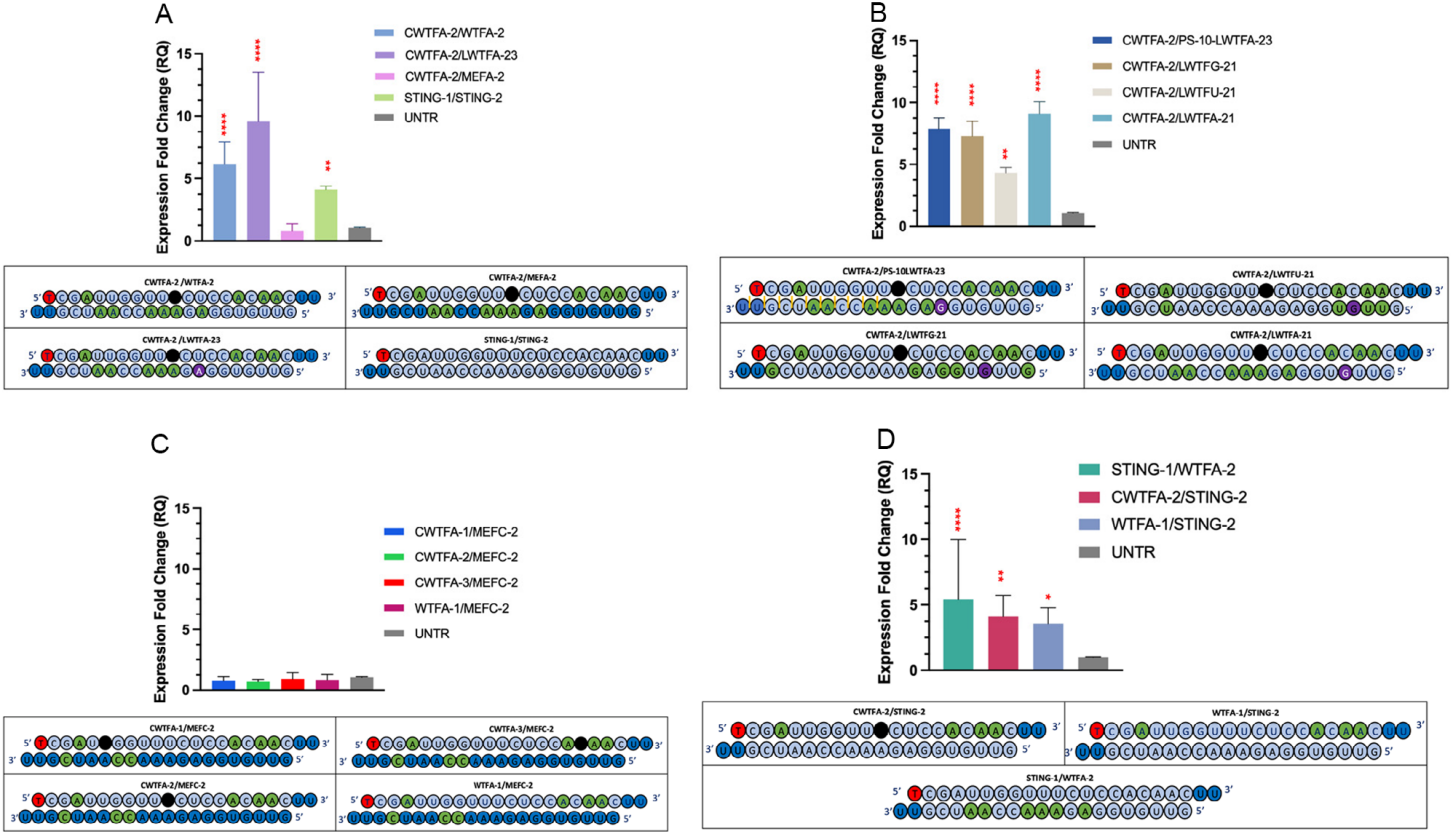
(A–D) Upregulation data of *STING* by saRNA duplexes at 10 nM, monitored 72 h post‐transfection in A549 cells. Expression fold change values (RQ) were normalized using GAPDH as a housekeeping gene. UNTR indicates the untreated control that was set to RQ = 1. Error bars represent standard deviation of minimum three independent biological replicates (mean), *p < 0.05, ***p < 0.005, ****p < 0.0001 one‐way ANOVA. (A) Upregulation of *STING* by STING‐1/STING‐2, and CWTFA‐2 annealed to WTFA‐2, LWTFA‐23, and MEFA‐2. (B) Upregulation of *STING* by CWTFA‐2 annealed to PS‐10‐LWTFA‐23, LWTFG‐21, LWTFU‐21, and LWTFA‐21. (C) Upregulation of *STING* by STING‐1/WTFA‐2, CWTFA‐2/STING‐2, and WTFA‐1/STING‐2. (D) Upregulation of *STING* by CWTFA‐1, CWTFA‐2, and CWTFA‐3, each annealed to MEFC‐2. RQ values of some saRNA duplexes are taken from data obtained in a previous publication but included in these figures for ease of comparison (11).

Activity of the unmodified duplex (STING‐1/STING‐2) was approximately fourfold. Adding a single C_3_‐alkyl propyl spacer and 2′‐F adenosine modifications in the passenger strand and 2′‐F adenosine modifications to the guide strand (CWTFA‐2/WTFA‐2) clearly increased *STING* mRNA expression levels (RQ 6.2) (Figure 2A). Adding an LNA to position 8 (CWTFA‐2/LWTFA‐23) of the guide strand further increased *STING* expression (Tables 1 and 2, Figure 2A). We observed that moving the LNA from position 8 to position 4 (in the seed region) and adding a 2′‐F adenosine to position 8 (CWTFA‐2/LWTFA‐21) maintained activity (Figure 2B). We also explored the effect of placing phosphorothioated linkages in half of the guide strand on the 3′‐end (CWTFA‐2/PS‐10‐LWTFA‐23). Interestingly, this duplex maintained strong gene activation (RQ = 7.4) (Figure 2B). Together, these data suggest a degree of chemical and positional versatility within the guide strand. However, we observed that a fully modified guide strand had a complete loss of activity (CWTFA‐2/MEFA‐2) (Figure 2A), which was expected given positions 2 and 14 were modified with 2′‐*O*‐Me and studies have shown that these modifications are incompatible with loading into AGO protein [42, 43].

To examine whether we could shuffle the 2′‐F modifications from adenosines to either guanosines or uridines, while keeping the single LNA intact at position 4, we generated guide strands LWTFU‐21 and LWTFG‐21, which contain 2′‐F uridines and 2′‐F guanosines, respectively. These designs were created in order to observe whether the 2′‐fluorination of a particular base would impact activity. *STING* activation with 2′‐F modifications on guanine was similar to 2′‐F on adenosine. However, 2′‐F modifications of uridines clearly reduced *STING* activation to levels similar to the unmodified STING‐1/STING‐2 duplex (Figure 2B). Taken together, these results show that 2′‐F modifications when incorporated as single nucleobases (2′‐fluorinated adenosine with or without phosphorothioates, 2′‐fluorinated guanosine, or 2′‐fluorinated cytosines) in combination with a single LNA on the guide strand yielded *STING* upregulation when paired with the passenger strand CWTFA‐2. This result is understandable, as the LNA modification is placed within the seed region and typically works well, as the seed region is central in target binding initiation.

To understand which modifications in the buildup from a 2′‐F adenosine partially modified oligo to a fully modified 2′‐F and 2′‐*O*‐Me modified active strand are detrimental to saRNA activity, we replaced the 2′‐F from adenosines to cytosines, with the remaining nucleobases as 2′‐*O*‐Me (CWTFA‐2/MEFC‐2, Table 1). As observed with CWTFA‐2/MEFA‐2, there was no *STING* activation with CWTFA‐2/MEFC‐2 (Figure 2A,C). These results showed that the pairing of a passenger strand associated with high gene upregulation (CWTFA‐2) with either fully modified guide strands like MEFA‐2 and/or MEFC‐2 does not trigger gene upregulation (Figure 2A,C). Again, this is due to how AGO2 does not tolerate 2′‐*O*‐Me in position 2 of the guide strand [42, 43]. These data support the intolerance of 2′‐*O*‐alkylated modifications at these positions.

To investigate whether changing the position of the C_3_ linker on the passenger strand could recover upregulation activity, we annealed guide strand MEFC‐2 to passenger strands CWTFA‐1 and CWTFA‐3, where we moved the C_3_‐propyl spacer from the central region to areas closer to the 5′ and 3′ ends, respectively. As shown in Figure 2C, this failed to rescue *STING* activation.

**FIGURE 3:**
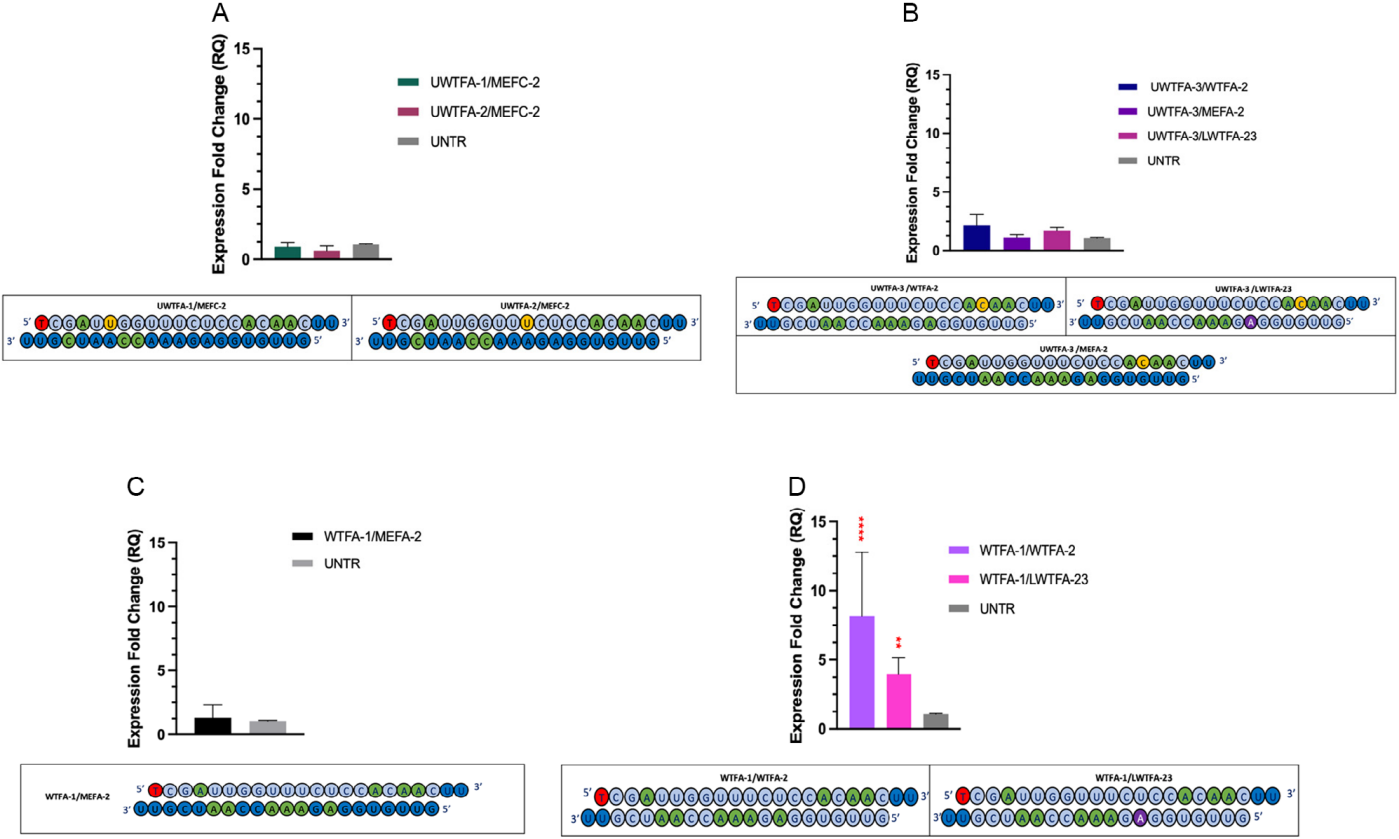
(A–D) Upregulation data of *STING* by saRNA duplexes at 10 nM, monitored 72 h post‐transfection in A549 cells. Expression fold change values (RQ) were normalized using GAPDH as a housekeeping gene. UNTR indicates the untreated control that was set to RQ = 1. Error bars represent standard deviation of minimum three independent biological replicates (mean), *p < 0.05, ***p < 0.005, ****p < 0.0001 one‐way ANOVA. (A) Upregulation of *STING* by UWTFA‐1 and UWTFA‐2 annealed to MEFC‐2. (B) Upregulation of *STING* by UWTFA‐3 annealed to WTFA‐2, LWTFA‐23, and MEFA‐2. (C) Upregulation of *STING* by WTFA‐1/MEFA‐2. (D) Upregulation of STING by WTFA‐1 annealed to WTFA‐2 and LWTFA‐23.

To assess if the destabilizing properties of an UNA at certain positions of the passenger strand could overcome the inhibitory effects of 2′‐*O*‐Me on positions 2 and 14 on AGO loading, we paired MEFC‐2 with UNA‐containing passenger strands, UWTFA‐1 (position 5) and UWTFA‐2 (position 10). Again, *STING* activation was not observed (Figure 3A). Duplex UWTFA‐3/MEFA‐2 was generated and tested to see if replacing 2′‐F modifications on cytosines to adenosines and shifting the UNA to position 16 on these passenger strand would have any effect. However, no statistical significant induction of expression was observed (RQ = 0.9) (Figure 3B). These results show that changing the position of the C_3_‐propyl linker or adding an UNA in the passenger strand does not recover gene upregulation activity of saRNA when the duplex has a fully 2′‐fluoro and 2′‐*O*‐Me chemically modified type guide strand (MEFA‐2 and MEFC‐2). While it is possible that modifying the position of the linker or UNA could enhance saRNA activity, any effects were likely overwritten by the effect of 2′‐*O*‐Me in positions 2 and 14 of the guide strand [42, 43].

Another passenger strand associated with strong gene upregulation from our previous study, WTFA‐1, was also paired with guide strands MEFA‐2 and MEFC‐2 to generate WTFA‐1/MEFA‐2 and WTFA‐1/MEFC‐2, in order to observe the effect of 2′‐F adenosines solely. The resultant RQ values increased marginally to 1.7 and 1.1, respectively, but still show no sign of significant upregulation (Figures 2C and 3C). This observation is in contrast to duplexes WTFA‐1/WTFA‐2 and WTFA‐1/LWTFA‐23, which have RQs of 8.1 and 4.0, respectively (Figure 3D), in addition to WTFA‐1/STING‐2 showing RQ of 3.6 (Figure 2D).

**FIGURE 4:**
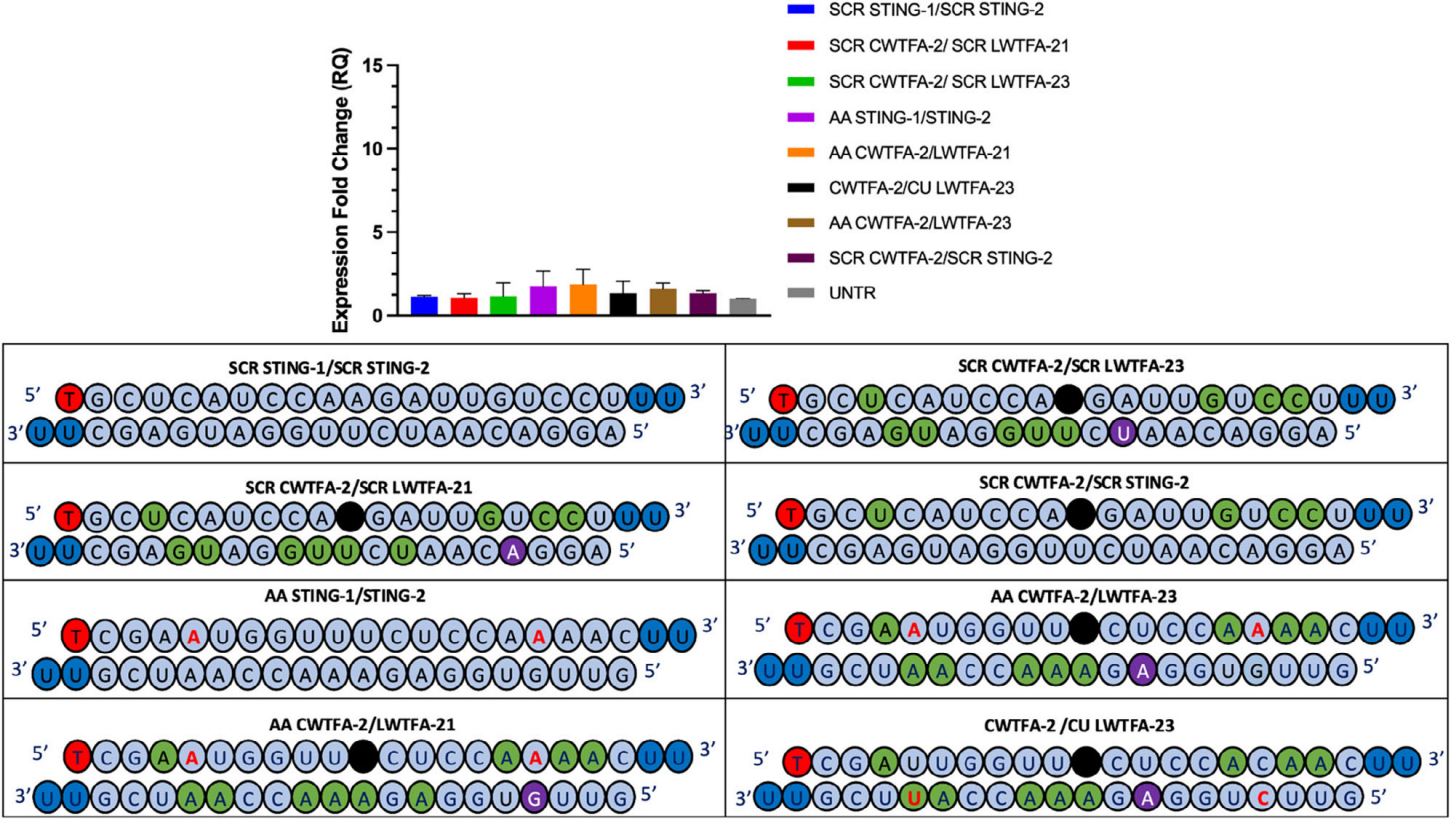
Upregulation data of *STING* by saRNA duplexes at 10 nM, monitored 72 h post‐transfection in A549 cells. Expression fold change values (RQ) were normalized using GAPDH as a housekeeping gene. UNTR indicates the untreated control that was set to RQ = 1. Upregulation of *STING* by scramble and mismatch control duplexes: SCR STING‐1/SCR STING‐2, SCR CWTFA‐2/SCR LWTFA‐21, SCR CWTFA‐2/SCR LWTFA‐23, AA STING‐1/STING‐2, AA CWTFA‐2/LWTFA‐21, CWTFA‐2/CU LWTFA‐23, AA CWTFA‐2/LWTFA‐23, and SCR CWTFA‐2/SCR STING‐2. RQ values of some saRNA duplexes are taken from data obtained in a previous publication but included in these figures for ease of comparison (11).

In order to confirm target gene specificity of these RNAs, we annealed scramble control duplexes SCR STING‐1/STING‐2, SCR CWTFA‐2/SCR LWTFA‐21, SCR CWTFA‐2/SCR LWTFA‐23, and SCR CWTFA‐2/SCR STING‐2 (Figure 4). These controls have the same nucleoside composition pattern as the test sequence, where the sequence is in a randomized order. Additional duplexes, such as AA STING‐1/STING‐2, AA CWTFA‐2/LWTFA‐21, AA CWTFA‐2/LWTFA‐23, and CWTFA‐2/CU LWTFA‐23 (Figure 4), were generated as mismatch controls, where there is a two‐base‐pair mismatch in either the passenger or guide strand (Figure 1). Results showed that the scramble controls did not upregulate *STING* expression (Table 2). For the mismatch controls, we observed that mismatches in the passenger strand only (AA STING‐1/STING‐2, AA CWTFA‐2/LWTFA‐21, AA CWTFA‐2/LWTFA‐23) had minimal upregulation (RQs ≤1.6, Figure 4). Mismatches in the guide strand (CWTFA‐2/CU LWTFA‐23) revealed no gene upregulation, with an RQ of 0.8 (Figure 4 and Table 2). Testing of these controls confirmed the sequence specificity of the test sequence to the target *STING*, and that data are not a result of chemical modification artifacts. While a mismatch in the passenger strand was not expected to have much effect, we observed that there was slight upregulation (AA STING‐1/STING‐2, AA CWTFA‐2/LWTFA‐21, AA CWTFA‐2/LWTFA‐23). These results emphasize how the guide strand is the strand driving upregulation, as the mismatch passenger strands were annealed to guide strands with no mismatches.

In conclusion, gene activation of *STING* can be impacted by chemical modification patterns and design, and its duplex melting temperature. To determine whether the saRNAs could act as siRNA substrates, we tested these same duplexes for gene silencing.

### 4.3 Gene Silencing Studies

As mentioned above, the saRNA was designed such that the active guide strand orientation was in the 3–5′ direction to the target sequence. As such, it was possible that the AGO–saRNA complex could bind to complementary mRNA targets in the cytoplasm. To first determine whether the active guide strand in the *STING* duplex could also perform RNA interference (RNAi), we designed and cloned a pGL3‐*STING* plasmid containing the gene target from *STING*—complementary to and targeted by the guide strand—into the 3′‐UTR of the luciferase reporter gene. The *STING* saRNA sequence used was the same as that used in a previous publication, corresponding to position 416bp downstream of the transcriptional start site (TSS) of the *STING* gene [11]. The saRNAs were transfected into HeLa cells along with pGL3‐*STING* and pRL‐SV40 plasmids where the percent of siRNA activity was measurable via reduction in luciferase activity. Gene silencing results of select saRNAs in A549 cells can be found in the SI (Figure S29).

**FIGURE 5:**
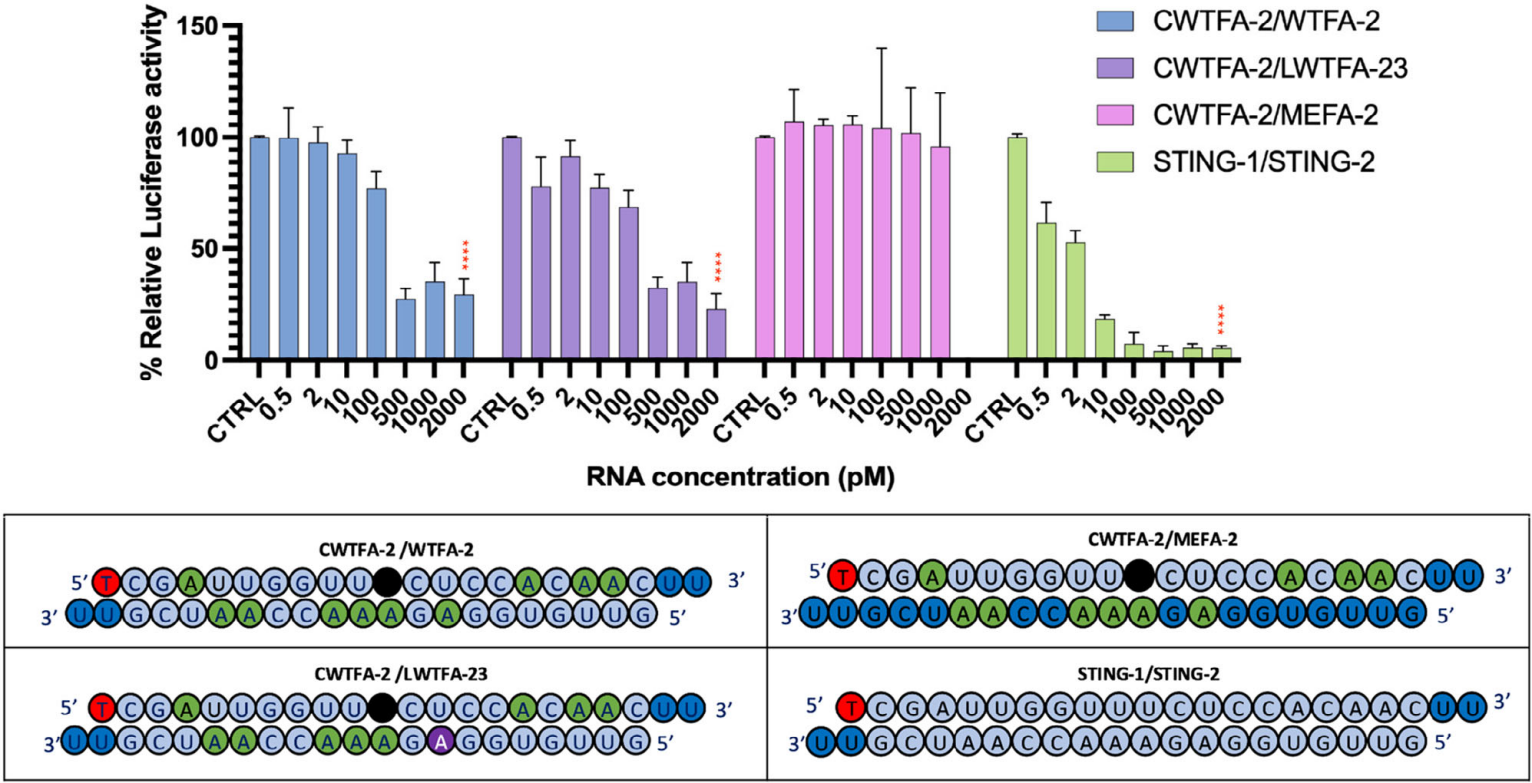
Gene silencing data of *STING* by RNA duplexes STING‐1/STING‐2 (WT) and CWTFA‐2 annealed to MEFA‐2, WTFA‐2, and LWTFA‐23 at various concentrations (pM), monitored 24 h post‐transfection in HeLa cells. CTRL indicates the untreated control, set to 100% luciferase expression, where ****p < 0.0001 one‐way ANOVA for the highest tested concentration. The same passenger strand with a C_3_ alkyl spacer was annealed to three different guide strands with different chemical modification designs, including 2′‐*O*‐methylated bases, 2′ fluorinated adenosines, and locked nucleic acid (LNA) to observe the effect of different guide strand designs on activity. *Firefly* luciferase activity was normalized to *Renilla* luciferase. Mean with error bars that represent standard deviation of minimum two independent biological replicates.

We first tested STING‐1/STING‐2 (least modified duplex), which demonstrated a fourfold upregulation of *STING* mRNA in A549 cells. As shown in Figure 5, this duplex acted as a highly potent siRNA, with an IC_50_ of 1.5 pM (Figure 3). From these results, we were curious to investigate whether medicinal chemistry patterns on the active guide strand could moderate the siRNA activity, while retaining the saRNA activity. As mentioned earlier, we did not expect chemical modification of the passenger strand to strongly modify siRNA activity as a) it is in a 5′ to 3′ orientation and b) we used an inverted dT on the 5′‐end of the passenger strand to inhibit its loading onto AGO, making it the inactive strand.

We tested the activity of the duplex with 2′‐F adenosine modifications to the guide strand (CWTFA‐2/WTFA‐2), with 2′‐F adenosines and LNA at position 8 (CWTFA‐2/LWTFA‐23), and a fully modified guide strand (CWTFA‐2/MEFA‐2), all with a C_3_‐propyl spacer in the central position of the passenger strand. We selected these, as all WTFA‐2 and LWTFA‐23 as passenger strands had demonstrated strong *STING* activation, while MEFA‐2 as the guide strand was not active. Both WTFA‐2 and LWTFA‐23 demonstrated dose‐dependent siRNA activity, though were not as potent as STING‐1/STING‐2, with IC_50_s of 197.3 and 330.3 pM, respectively (Table 2 and Figure 5). The fully modified guide strand, however, did not demonstrate any siRNA activity, even at high doses (CWTFA‐2/MEFA‐2). Again, this is attributed to the effect of 2′‐*O*‐Me in position 2 on the guide strand, incompatible with strand loading into the AGO protein [42, 43].

**FIGURE 6:**
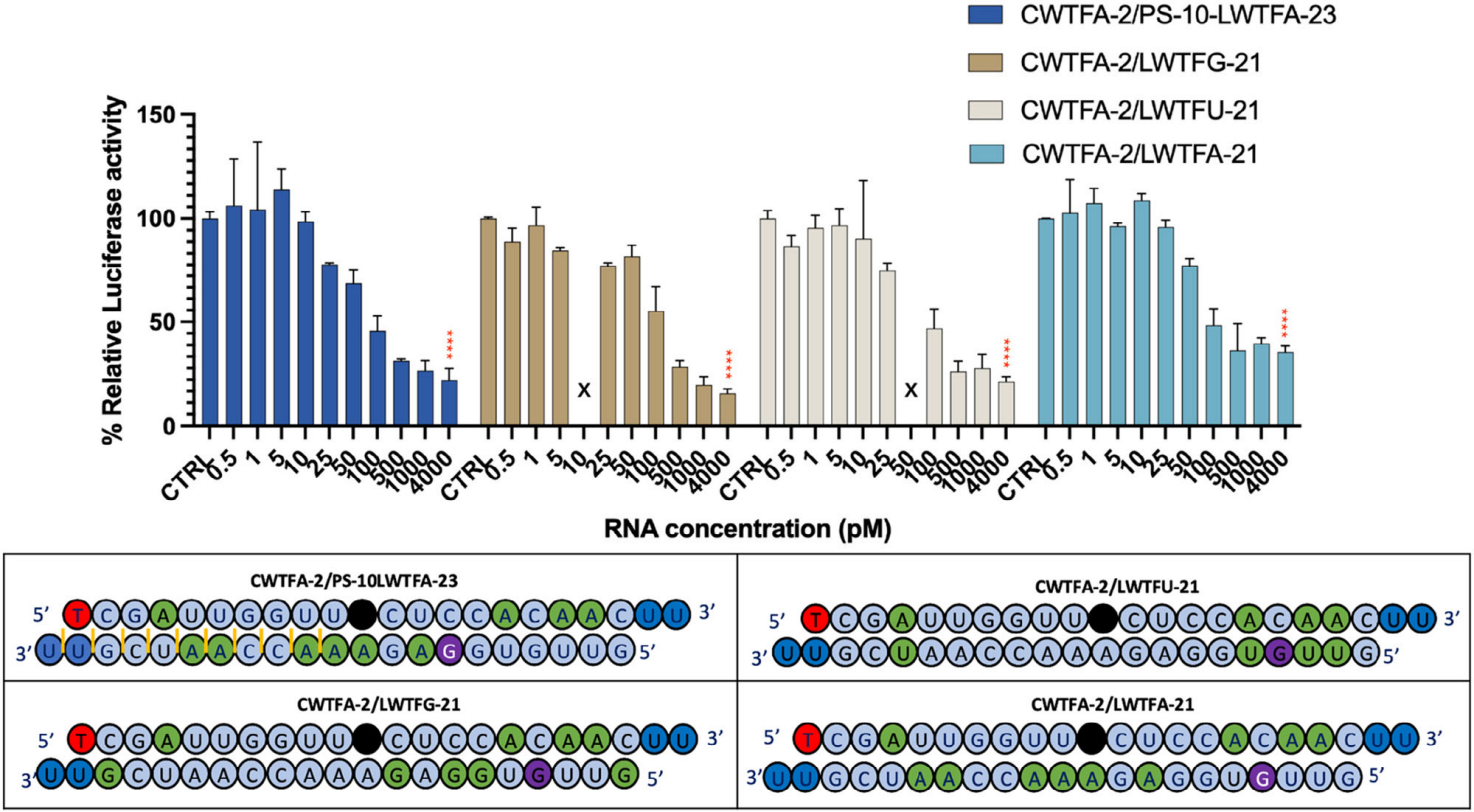
Gene silencing data of *STING* by RNA duplexes CWTFA‐2 annealed to PS‐10‐LWTFA‐23, LWTFG‐21, LWTFU‐21, and LWTFA‐21 at various concentrations (pM), monitored 24 h post‐transfection in HeLa cells. CTRL indicates the untreated control, set to 100% luciferase expression, where ****p < 0.0001 one‐way ANOVA for the highest tested concentration. The same passenger strand with a C_3_ alkyl spacer was annealed to four different guide strands with different chemical modification designs, including 2′ fluorinated adenosines, uridines, guanosines, locked nucleic acid (LNA), and phosphorothioate backbone at the 5′‐end to observe the effect of different guide strand designs on activity. *Firefly* luciferase activity was normalized to *Renilla* luciferase. Mean with error bars that represent standard deviation of minimum two independent biological replicates. The “*x*” represents no data at that concentration for the respective duplex.

We then examined whether some of the strongest *STING* activators had siRNA activity, specifically 2′‐F on either adenosines, guanosines, or uridines on the guide strand (Figure 6). We were interested to see if these 2′‐fluorinated base patterns would influence gene silencing. All three duplexes showed clear silencing activity, CWTFA‐2/LWTFA‐21 had an IC_50_ of 385.4 pM, while CWTFA‐2/LWTFG‐21 and CWTFA‐2/LWTFU‐21 had IC_50_s of 167.2 and 143.5 pM, respectively (Table 2 and Figure 6). Overall, there were no appreciable differences in siRNA activity between the duplex RNAs as they were all within the same order of magnitude.

Additionally, we tested CWTFA‐2/PS‐10‐LWTFA‐23 (RQ = 7.4), which is the same as CWTFA‐2/LWTFA‐23, but includes thiolations in the backbone from the central position to the 3′‐end in the guide strand. This duplex had an IC_50_ of 171.8 pM (Table 2 and Figure 6). In comparison, CWTFA‐2/LWTFA‐23 (RQ = 9.0) had an IC_50_ of 197.3 pM. Overall, both upregulation and silencing activity were observed for the duplexes, the melting temperature ranged between 52°C and 72°C. We previously observed that a melting temperature range of around 65°C–72°C was optimal for gene activation [11]. These results suggest that most of these duplexes also fall within that optimal range for upregulation and still promote gene silencing. Melting temperatures within this range are critical for AGO loading and unwinding, as outlined as one of the essential parameters in both saRNA and siRNA designs [44, 45].

**FIGURE 7:**
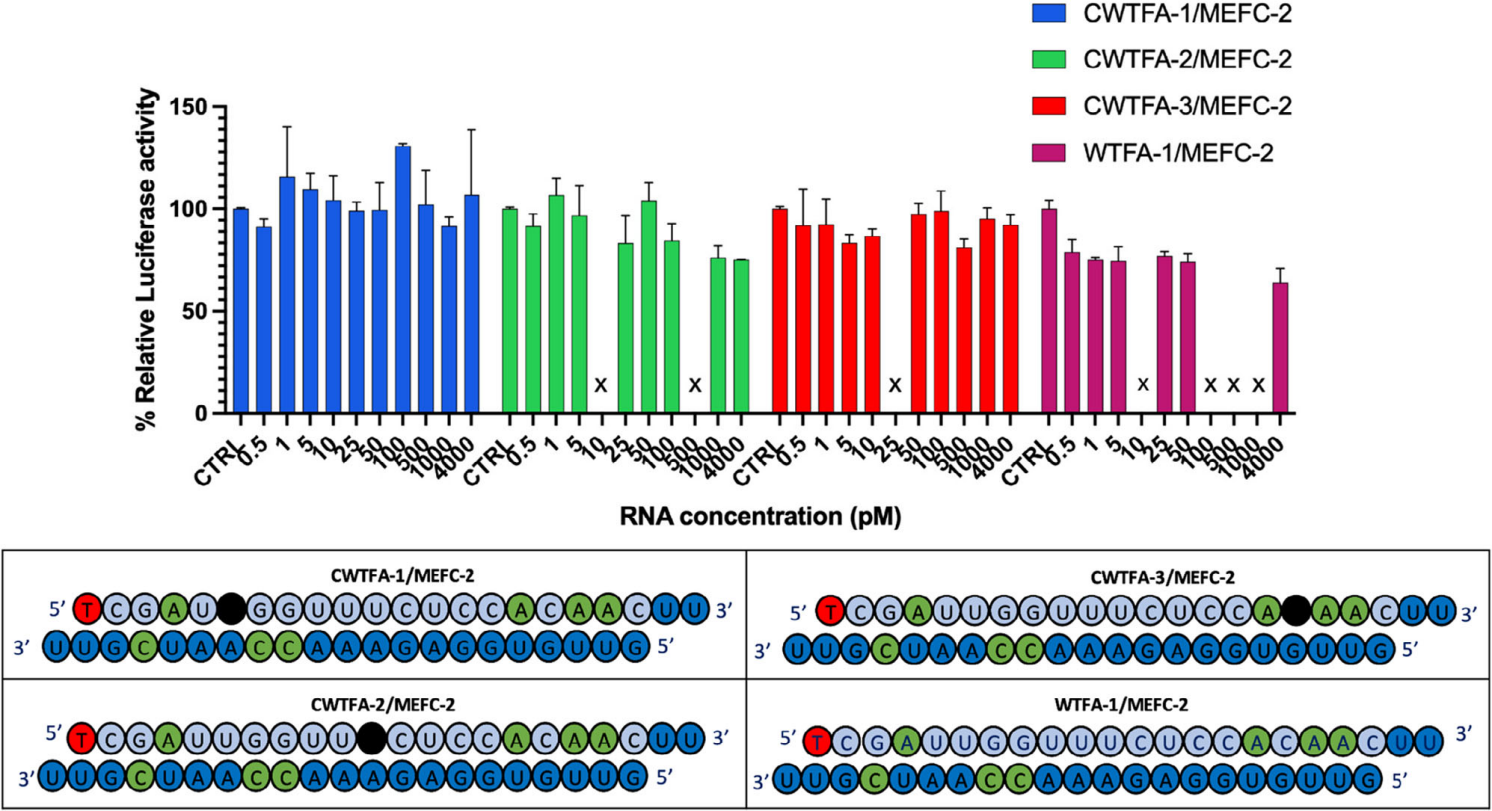
Gene silencing data of *STING* by RNA duplexes CWTFA‐1‐3, each annealed to MEFC‐2 at various concentrations (pM), monitored 24 h post‐transfection in HeLa cells. CTRL indicates the untreated control, set to 100% luciferase expression. The passenger strands with 2′‐fluorinated adenosines and C_3_ alkyl linker at different positions were each annealed to the same guide strand with 2′‐fluorinated adenosines and 2′‐*O*‐methyl modifications to observe the effect of different passenger strand linker positions on activity. *Firefly* luciferase activity was normalized to *Renilla* luciferase. Mean with error bars that represent standard deviation of minimum two independent biological replicates. Concentrations at which a duplex is missing a bar indicate no data for that duplex at that concentration. The “*x*” represents no data at that concentration for the respective duplex.

**FIGURE 8:**
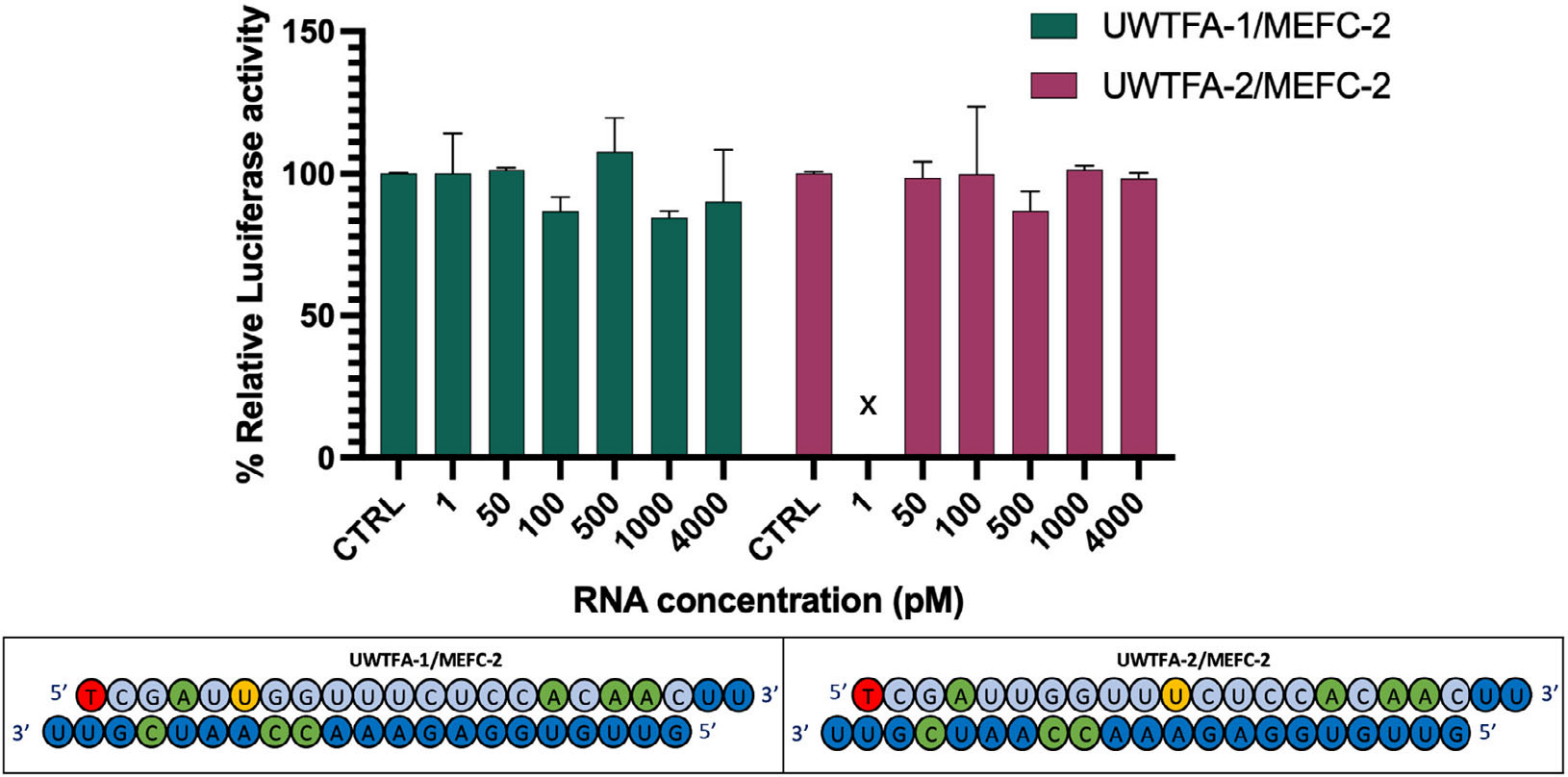
Gene silencing data of *STING* by RNA duplexes UWTFA‐1 and UWTFA‐2 each annealed to MEFC‐2 at various concentrations (pM), monitored 24 h post‐transfection in HeLa cells. CTRL indicates the untreated control, set to 100% luciferase expression. The passenger strands with 2′‐fluorinated adenosines and unlocked nucleic acid (UNA) at different positions were each annealed to the same guide strand with 2′‐fluorinated adenosines and 2′‐*O*‐methyl modifications to observe the effect of different passenger strand UNA positions on activity. *Firefly* luciferase activity was normalized to *Renilla* luciferase. Mean with error bars that represent standard deviation of minimum two independent biological replicates. The “*x*” represents no data at that concentration for the respective duplex.

We then wanted to test if the presence and location of a C_3_‐propyl linker within the passenger strand would affect silencing ability when paired with the fully modified guide strand, MEFC‐2. We anticipate with the fully modified guide strands that poor to no silencing would occur. As expected, with duplexes CWTFA‐1/MEFC‐2, CWTFA‐2/MEFC‐2, CWTFA‐3/MEFC‐2, and WTFA‐1/MEFC‐2, no dose–responses were found illustrating that these duplexes did not show any indication of silencing activity (Figure 7). This trend was observed again in a subsequent set, where guide strand MEFC‐2 was paired with passenger strands, UWTFA‐1 and UWTFA‐2, to generate UWTFA‐1/MEFC‐2 and UWTFA‐2/MEFC‐2. The melting temperature for these fully modified guide strands (MEFA‐2 and MEFC‐2)‐type paired duplexes ranged from 64°C to 82°C (Table 2 and Figure 8). Although some of these duplexes do fall within the “ideal” *T*_*m*_ range that we observed for strong activation in our previous study, this is not translated to gene activation nor gene silencing activity when the guide strand is completely chemically modified [11].

**FIGURE 9:**
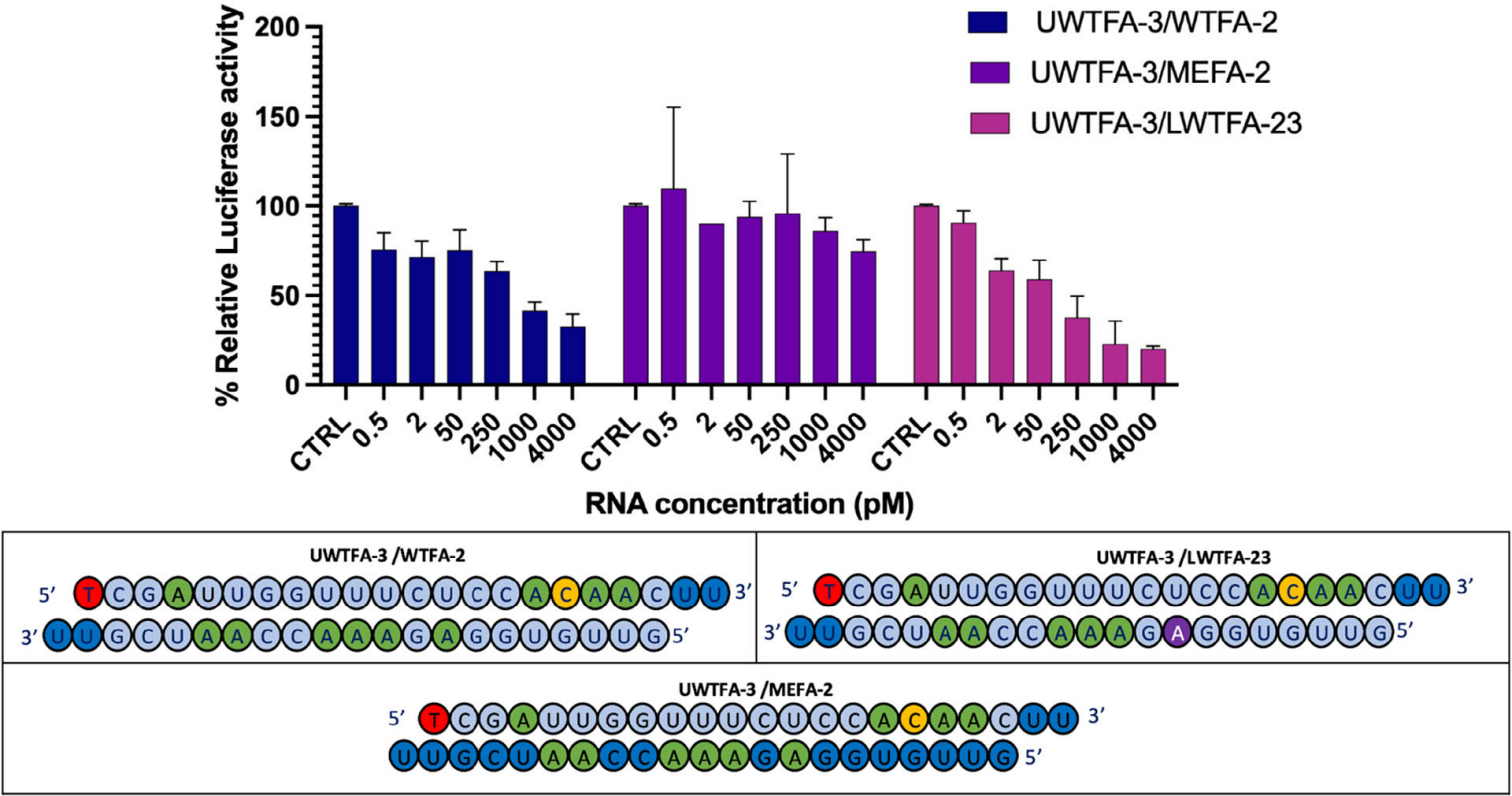
Gene silencing data of *STING* by RNA duplexes UWTFA‐3 annealed to WTFA‐2, MEFA‐2, and LWTFA‐23 at various concentrations (pM), monitored 24 h post‐transfection in HeLa cells. CTRL indicates the untreated control, set to 100% luciferase expression, where ****p < 0.0001 one‐way ANOVA for the highest tested concentration. The same passenger strand with an unlocked nucleic acid was annealed to three different guide strands with different chemical modification designs, including 2′‐*O*‐methylated bases, 2′ fluorinated adenosines, and locked nucleic acid (LNA) to observe the effect of different guide strand designs on activity. *Firefly* luciferase activity was normalized to *Renilla* luciferase. Mean with error bars that represent standard deviation of minimum two independent biological replicates.

Next, we were interested in changing the position of the UNA within the passenger strands and comparing it to partly modified guide strands. Thus, UWTFA‐3 was annealed with WTFA‐2, LWTFA‐23, and MEFA‐2 to generate UWTFA‐3/WTFA‐2, UWTFA‐3/LWTFA‐23, and UWTFA‐3/MEFA‐2. UWTFA‐3/WTFA‐2′s dose–response showed an IC_50_ of 497.6 pM and UWTFA‐3/LWTFA‐23 had an IC_50_ of 58.5 pM. UWTFA‐3/MEFA‐2 showed no silencing activity and had no measurable IC_50_. The melting temperature of each duplex in this set ranged from 71°C to 73°C (Table 2 and Figure 9). Data revealed that although reduced in comparison to duplexes like CWTFA‐2/LWTFA‐23, duplexes UWTFA‐3/WTFA‐2 and UWTFA‐3/LWTFA‐23 both show clear dose‐dependent gene silencing.

**FIGURE 10:**
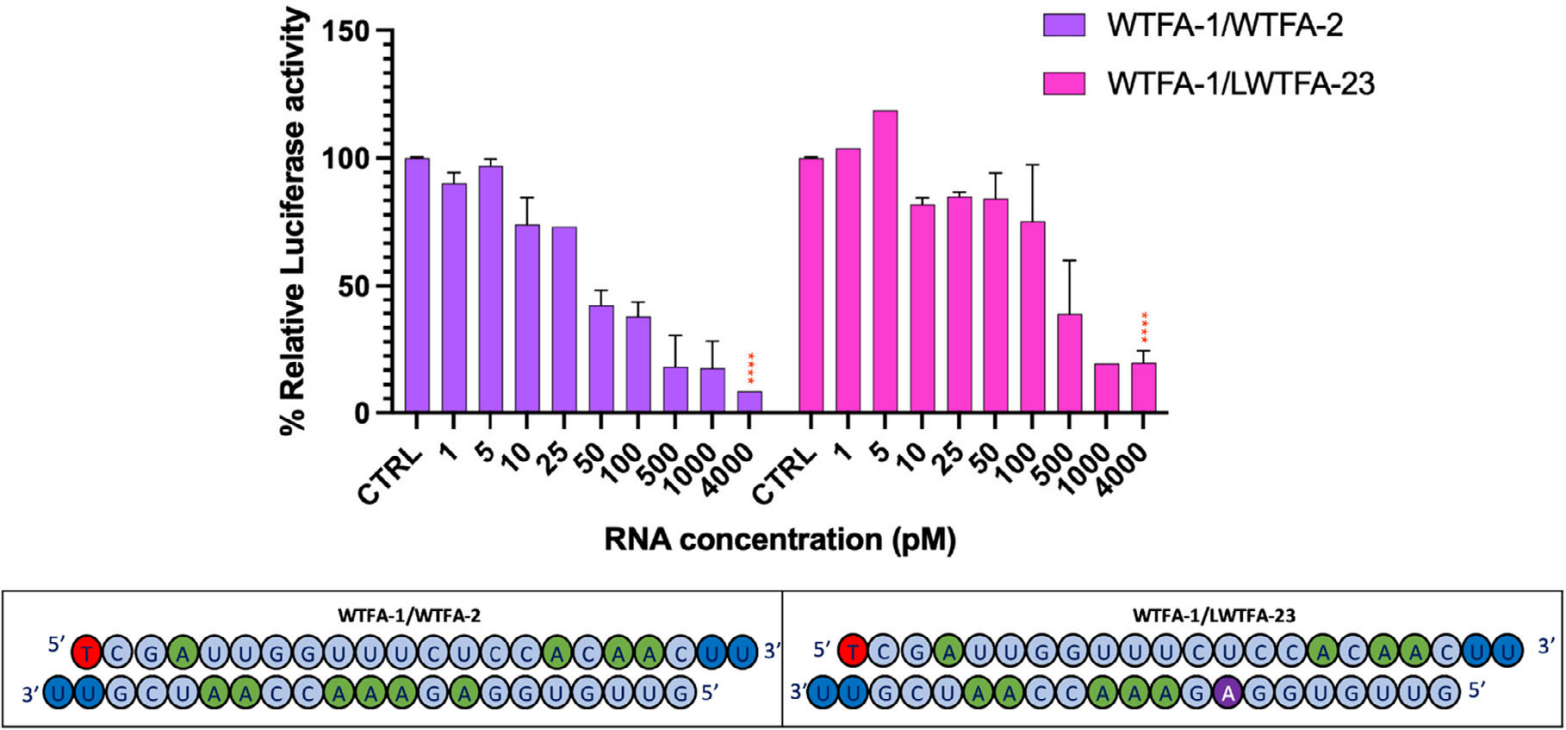
Gene silencing data of *STING* by RNA duplexes WTFA‐1 annealed to WTFA‐2 and LWTFA‐23 at various concentrations (pM), monitored 24 h post‐transfection in HeLa cells. CTRL indicates the untreated control, set to 100% luciferase expression, where ****p < 0.0001 one‐way ANOVA for the highest tested concentration. The same passenger strand with 2′‐fluorinated adenosines was annealed to two different guide strands with different chemical modification designs, including 2′ fluorinated adenosines and locked nucleic acid (LNA) to observe the effect of different guide strand designs on activity. *Firefly* luciferase activity was normalized to *Renilla* luciferase. Mean with error bars that represent standard deviation of minimum two independent biological replicates.

We then turned our attention to modifications of the guide strand and whether modifications herein could retain saRNA activity, while minimizing siRNA activity. We used the passenger strand with the inverted dT and 2′‐F adenosines with guide strands that had a) 2′‐F adenosines (WTFA‐1/WTFA‐2), or b) 2′‐F adenosines with an LNA at position 8 (WTFA‐1/LWTFA‐23), or c) the fully modified guide strand (WTFA‐2/MEFA‐2). As shown in Figure 10, both duplexes demonstrated a 25% reduction in luciferase activity at 10 pM. While siRNA activity increased in a dose‐dependent manner for WTFA‐1/WTFA‐2, siRNA activity did not begin for WTFA‐1/LWTFA‐23 until 500 pM. These data suggest that the LNA at position 8 of the guide strand was at least partially able to restrain siRNA activity. However, as shown in Figure 3D, the WTFA‐2 strand was also more effective at *STING* induction, suggesting that the LNA modifies duplex activity overall rather than specifically titrating saRNA versus siRNA activity.

**FIGURE 11:**
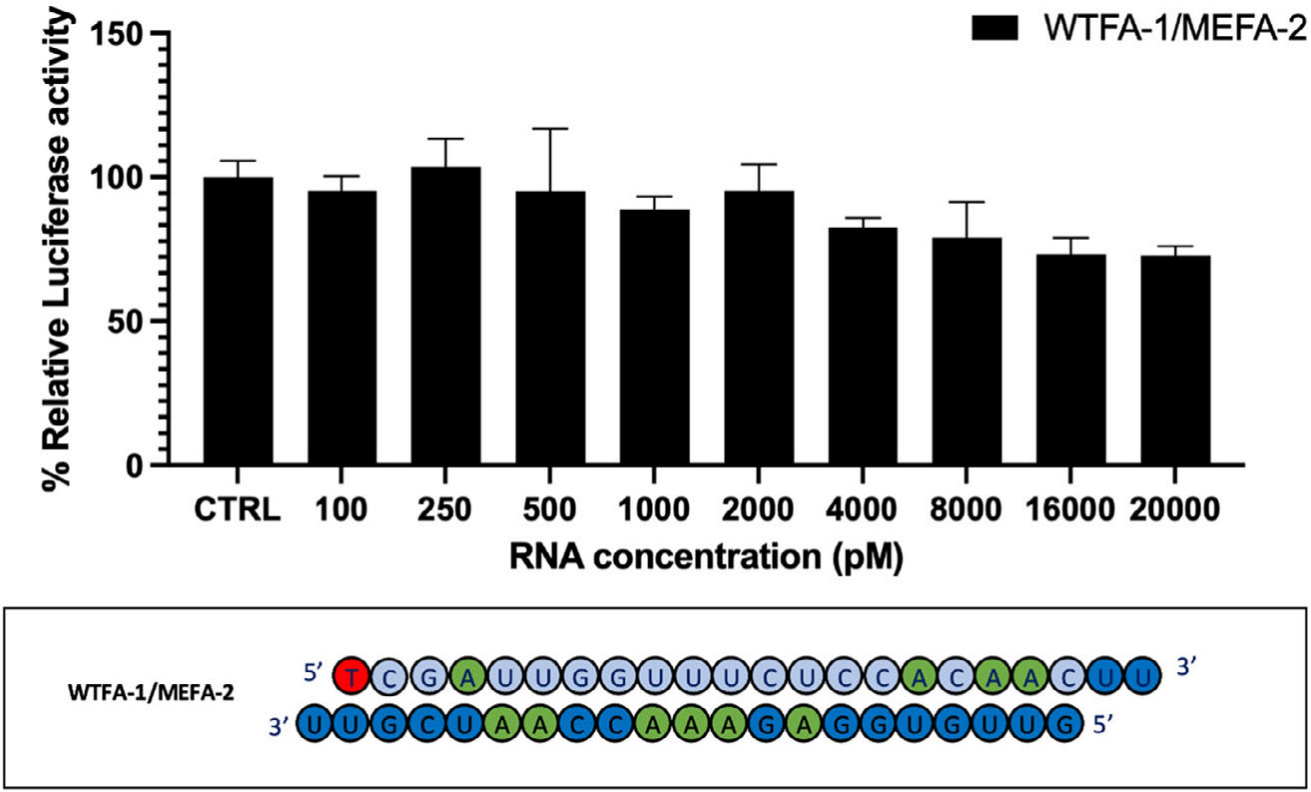
Gene silencing data of *STING* by RNA duplex WTFA‐1/MEFA‐2 at various concentrations (pM), monitored 24 h post‐transfection in HeLa cells. CTRL indicates the untreated control, set to 100% luciferase expression. The passenger strand with 2′‐fluorinated adenosines was annealed to a guide strand with 2′‐fluorinated adenosines and 2′‐*O*‐methyl modifications. *Firefly* luciferase activity was normalized to *Renilla* luciferase. Mean with error bars that represent standard deviation of minimum two independent biological replicates.

We then tested WTFA‐1/MEFA‐2 alongside WTFA‐1/WTFA‐2 and WTFA‐1/LWTFA‐23 to further observe the extent of how saRNAs could perform silencing and to test duplexes with minimally modified passenger strands and compare to those with a UNA or C_3_ (Figures 10 and 11). WTFA‐1/WTFA‐2 and WTFA‐1/LWTFA‐23 both demonstrated evident dose‐dependent silencing and had IC_50_s of 55.8 and 317.3 pM, with melting temperatures of 73°C and 79°C, respectively. WTFA‐1/MEFA‐2 showed no signs of gene silencing and possessed a melting temperature of 81°C (Table 2). This observation highlights that having an LNA in the guide strand slightly hinders gene silencing (WTFA‐1/LWTFA‐23 compared to WTFA‐1/WTFA‐2).

**FIGURE 12:**
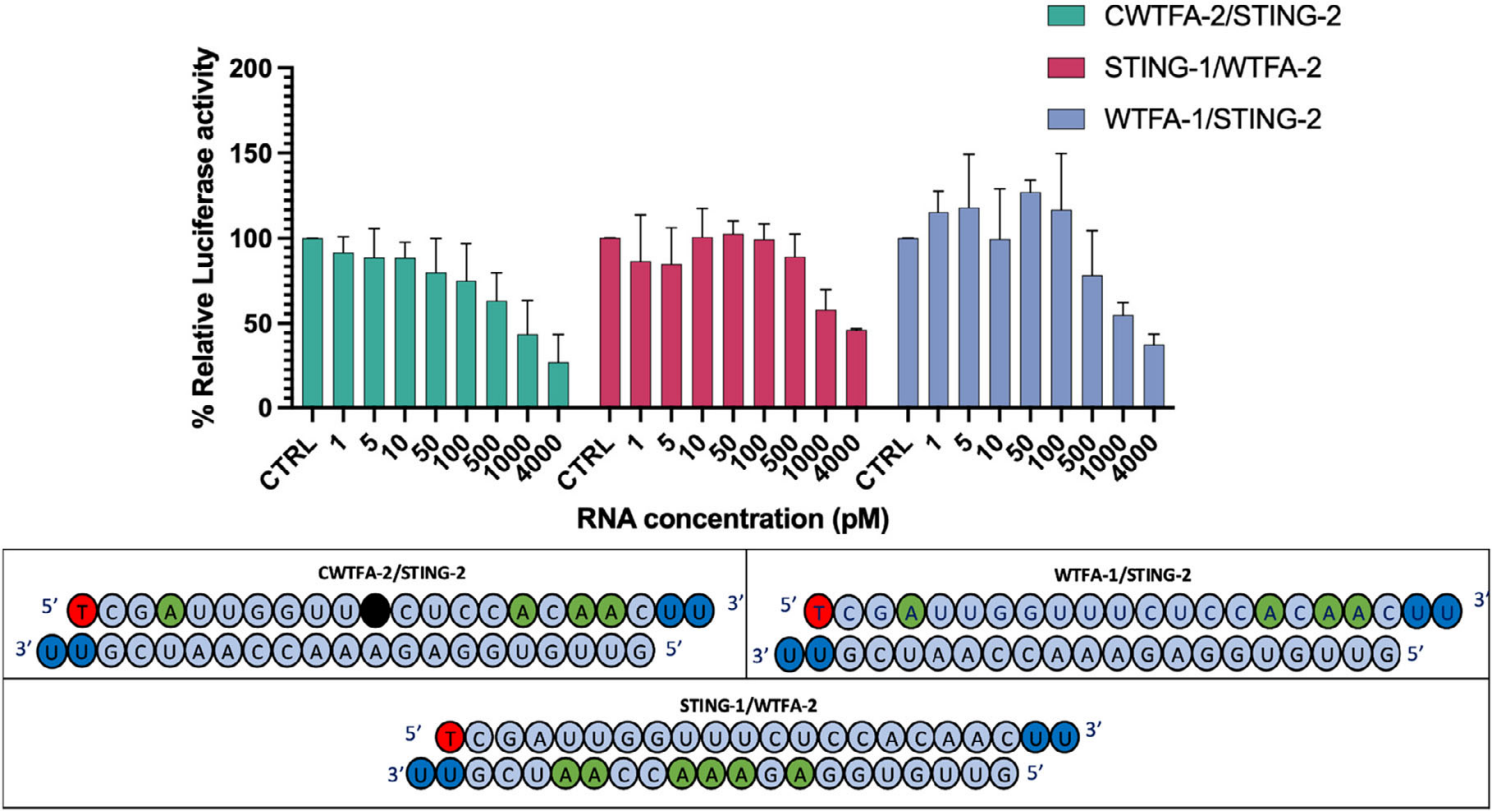
Gene silencing data of *STING* by RNA duplexes CWTFA‐2/STING‐2, STING‐1/WTFA‐2, and WTFA‐1/STING‐2 at various concentrations (pM), monitored 24 h post‐transfection in HeLa cells. CTRL indicates the untreated control, set to 100% luciferase expression, where ****p < 0.0001 one‐way ANOVA for the highest tested concentration. The passenger strands with 2′‐fluorinated adenosines and different C_3_ alkyl linker presence alongside wildtype passenger strand were annealed to a guide strand with 2′‐fluorinated adenosines and to wildtype guide strand to observe the effect of only one lightly modified strand in different combinations. *Firefly* luciferase activity was normalized to *Renilla* luciferase. Mean with error bars that represent standard deviation of minimum two independent biological replicates.

Finally, we tested CWTFA‐2/STING‐2, STING‐1/WTFA‐2, and WTFA‐1/STING‐2 to observe the effects of minimal chemical modifications on both the passenger and guide strands separately. CWTFA‐2/STING‐2, STING‐1/WTFA‐2, and WTFA‐1/STING‐2 had melting temperatures of 52°C, 73°C, and 77°C, respectively (Table 2). They each exhibited evident dose–response silencing ability, with IC_50_s of 786.4, 2720, and 1783 pM, respectively (Figure 12).

**FIGURE 13:**
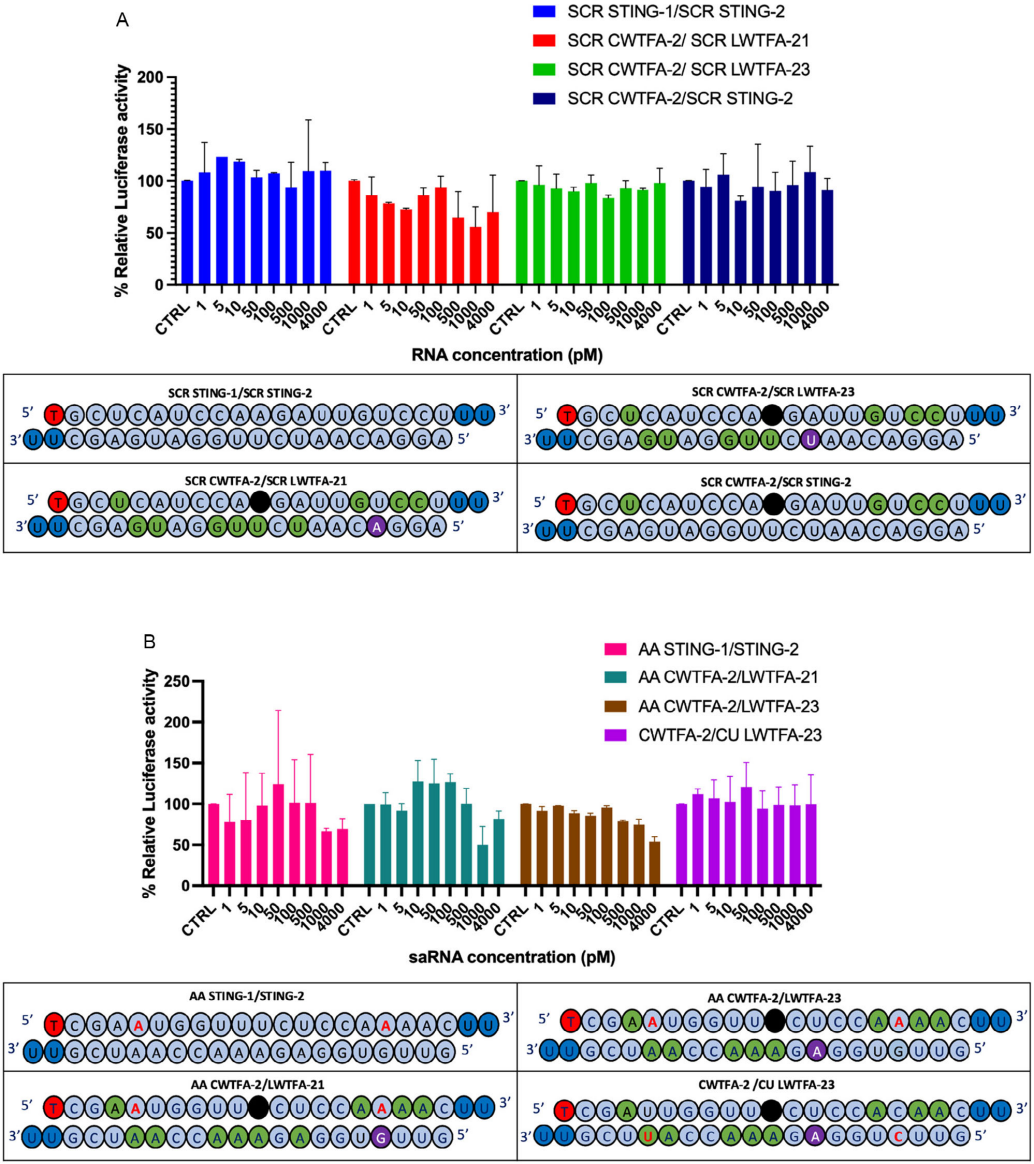
Gene silencing data of *STING* by control RNA scramble and mismatch control duplexes (A) SCR STING‐1/SCR STING‐2, SCR CWTFA‐2/SCR LWTFA‐21, SCR CWTFA‐2/SCR LWTFA‐23, and SCR CWTFA‐2/SCR STING‐2. (B) AA STING‐1/STING‐2, AA CWTFA‐2/LWTFA‐21, CWTFA‐2/CU LWTFA‐23, AA CWTFA‐2/LWTFA‐23. Duplexes were tested at various concentrations (pM), monitored 24 h post‐transfection in HeLa cells.CTRL indicates the untreated control, set to 100% luciferase expression. Scrambled passenger strands were annealed to scrambled guide strands with modification content identical to each scramble control′s parent duplex. Two‐base mismatch passenger strands were annealed to parent guide strands. Parent passenger strands were annealed to two‐base mismatch guide strands. *Firefly* luciferase activity was normalized to *Renilla* luciferase. Mean with error bars that represent standard deviation of minimum two independent biological replicates.

To confirm that all observations were not the product of nonspecific gene silencing or artifacts due to chemical modifications, mismatch and scramble sequences were designed. The scramble controls have the same nucleoside composition pattern as the test sequence, but the sequence is randomized. The mismatch controls have two bases that are mismatched in either the passenger or guide strand. Testing shows that none of the scramble controls resulted in gene silencing (Figure 13A). Of the controls that showed very little activity (RQ < 1.6), they were the duplexes that possessed a mismatched passenger strand paired with a nonmismatched guide strand (ex. AA CWTFA‐2/LWTFA‐2, Figure 13B). Such results were unsurprising, as a nonmismatched guide strand would potentially exhibit some degree of binding to its target, as complete or near to complete target complementarity is required for RNAi [11, 29, 46, 47]. Such observations continued in silencing assays, where the SCR STING‐1/SCR STING‐2, SCR CWTFA‐2/SCR STING‐2, SCR CWTFA‐2/SCR LWTFA‐21, and SCR CWTFA‐2/SCR LWTFA‐23 scramble duplexes showed no *STING* knockdown (Table 2).

The control with a mismatched guide—CWTFA‐2/CU LWTFA‐23, which has a mismatch within the seed region—also showed no silencing activity. Controls with mismatches in only the passenger strand, AA STING‐1/STING‐2, AA CWTFA‐2/LWTFA‐21, and AA CWTFA‐2/LWTFA‐23, possessed some degree of gene silencing, as expected. These control duplexes demonstrate that similar to gene activation, gene silencing is sequence specific and its activity is dependent on the chemical modifications present. Further, while mismatches in flanking regions are typically well tolerated and still allow for miRNA‐like activity, a seed region mismatch will impact both RNAi and mi‐RNA‐like mechanisms to the point of no activity.

In statistical analysis for the entire set of silencing data, dose–response curves differed significantly (extra sum‐of‐squares *F* test, *p *= 0.0001), where, null hypothesis (*H*_0_): all curves share the same IC_50_, and alternate hypothesis (*H*_1_): at least one curve has a different IC_50_ (*p *< 0.05). In conjunction with each saRNA duplex's IC_50_ value and confidence interval (see Table 2), their respective dose–response curves can be observed in the SI (Figures S11–S19).

**FIGURE 14:**
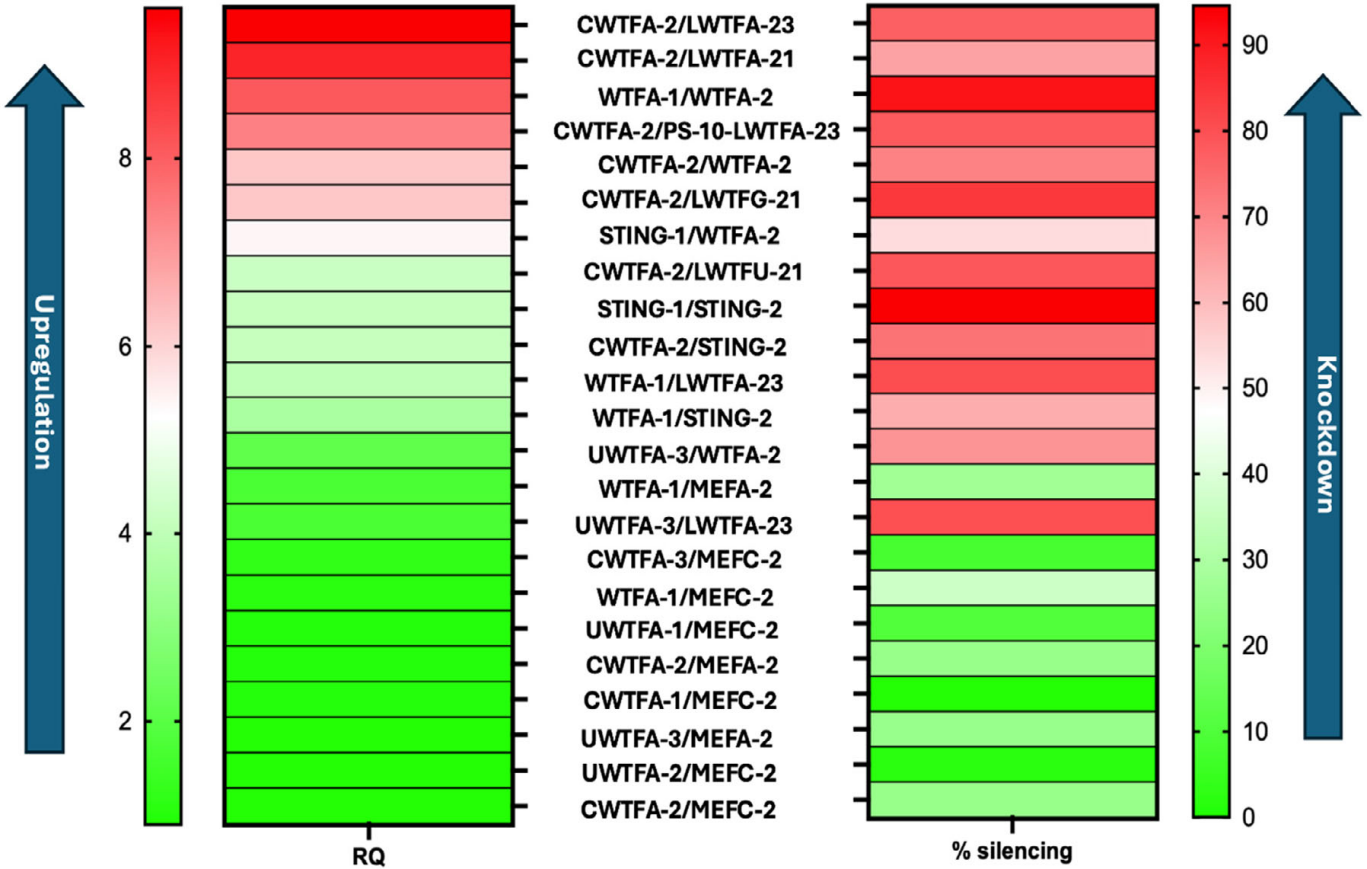
Heatmaps of dsRNA duplex upregulation and silencing activity. RQ heatmap represents the expression fold change of *STING* at 10 nM after 72 h, and % silencing represents the percent of luciferase knocked down at the highest duplex concentration tested (4 nM⪯) after 24 h. On each gradient, a stronger shade of red indicates stronger activity; strong upregulation for RQ, and strong gene knockdown for % silencing. Green indicates weaker activity, weak upregulation for RQ, and poor gene knockdown for % silencing. Heatmap data were generated using data from Table 2 for ease of trend identification.

To observe the overall effect of upregulation and silencing activity from a particular RNA, Figure 14 represents a heatmap of the cumulative data.

## 5 Discussion

To the best of our knowledge, this marks the first study that uses a RNAi reporter assay as a surrogate readout for potential silencing effects from duplex RNAs designed for activation as an saRNA. In complementary studies, some existing work in the literature has come across instances of siRNAs intended for gene silencing turned out to perform gene upregulation instead, directly or indirectly [48, 49, 50]. Our observations highlight that in general, strong gene activating saRNAs also are capable of strong gene silencing, whereas poor gene activator saRNAs are also poor gene silencers.

The degree of activity is governed by the number and type of chemical modifications, especially within the guide strand. Table 2 categorizes the activity of the duplex RNAs based on both activation and silencing. Strong activators were defined as having an RQ > 2, and strong silencing activity correlated to IC_50_s within <1500 pM. Moderate activators were defined as having an RQ between 1.2 and 2.0, and moderate silencing activity was defined as IC_50_s between 1500 and 3000 pM. IC_50_ curves for each duplex can be found in the SI (Figures S11–S19). Finally, weak activators had RQs < 1.2, and poor silencers had IC_50_s above 3000 pM, or IC_50_s that could not be calculated (NA—nonapplicable). The RQ category parameters were set based on the typical concentration ranges used in RNAa experiments that use concentrations in the low nanomolar range for activity testings [11].

Figures 2A,C and 3A–C, show that when RNA duplexes are highly modified with a guide strand, composed of only 2′‐*O*‐Me and three to six units of 2′‐F modifications—and no 2′‐*O*‐Me in the 2′‐position—no gene activation nor gene silencing occurs. Figures 5, 7–9, and 11 show that these RNAs that did not trigger upregulation such as CWTFA‐2/MEFA‐2, and other duplexes containing a fully modified guide strand (MEFA‐2 and MEFC‐2), were also not gene silencers, consistent with impaired AGO loading as previously described [38, 39]. RNA duplexes that are partially modified on the guide strand exhibit good activity and gene silencing when paired with a variety of passenger strands. For example, RNAs with higher RQ values demonstrate stronger silencing ability of the duplexes tested such as CWTFA‐2/LWTFA‐23 (Figures 2A and 5).

Delving deeper, we first examined the effect of 2′‐F nucleosides. From our previous study, we focused on 2′‐F modification on only the adenosine nucleobases [11]. In this study, we explored the effect of 2′‐F modification within guanosines and uridines to create CWTFA‐2/LWTFG‐21 and CWTFA‐2/LWTFU‐21, respectively. The melting temperatures for both CWTFA‐2/LWTFG‐21 and CWTFA‐2/LWTFU‐21 are slightly lower than CWTFA‐2/LWTFA‐21 by around 4°C for both. However, we observe that CWTFA‐2/LWTFA‐21, CWTFA‐2/LWTFG‐21, and CWTFA‐2/LWTFU‐21 all have strong activation (RQ > 4) and strong gene silencing responses (IC_50_s of 150 pM for both) (Figures 2B and 6). Additionally, we were interested in the effects of phosphorothioate backbone modifications on one of our saRNA designs and their upregulation ability. CWTFA‐2/PS‐10‐LWTFA‐23 demonstrates strong upregulation and silencing with a melting temperature (68°C) similar to that of STING‐1/STING‐2 (68°C) and CWTFA‐2/LWTFA‐23 (68°C), thus highlighting that this phosphorothioate backbone modification does not impede gene activation or gene silencing activity. This is unsurprising, as phosphorothioate backbone modifications are well tolerated in siRNAs, especially in the terminal regions and improve cellular uptake [51, 52].

Duplexes with melting temperatures within the “ideal” optimal range (65°C–72°C) for strong gene activation were also able to perform gene silencing, highlighting that early steps in their mechanism may overlap. In RNAi, AGO2 has been studied to receive the entire siRNA within RISC prior to siRNA passenger strand cleavage. Matranga et al. postulated two distinct mechanisms, by which the siRNA passenger strand was the AGO2 cleavage substrate. One mechanism was that upon siRNA strand separation, mature RISC is formed, in *trans*. The second mechanism envisioned the formation of a mature RISC prior to strand dissociation, so‐called *cis* loading [53]. The possibility of the pathway eventually leading to an active saRNA complex, potentially differing from that which produces an active siRNA complex, would be an avenue of great interest.

Finally, in general, we observe similar correlations between gene activation and gene silencing with the other RNAs studied. Figure 14 heatmap highlights trends between upregulation (% *STING* expression, RQ) and silencing (% knockdown), where we can see that duplexes that perform well at one mechanism (e.g., activation) generally perform well at the other mechanism (e.g., silencing). On each heatmap gradient, a deeper shade of red indicates stronger activity, where on the RQ heatmap, a deeper red indicates greater expression fold change, and on the % silencing heatmap, a deeper red indicates greater gene knockdown. A deeper shade of green indicates decreasing gene expression and decreasing gene knockdown on their respective heatmaps. White was used as the middle transition shade to better allow visualization between the red and green gradients.

We believe that understanding the full biological output of short duplexes in the cell is important, because RNAs, designed to be siRNAs, may have an unknown consequence in gene activation. Therefore, if one is trying to silence a particular transcript, one may need to consider the off‐target effects of gene activation of the same or similar targets. On the other hand, if one is designing an saRNA, one should take into account any potential gene silencing mechanisms that could result in decreased mRNA throughput or off‐target silencing. For example, if the target site on the desired gene is also on a mature mRNA, potential RNAi involvement must be considered, and we must find a way to design an saRNA to “bypass” the RNAi route in order to maximize saRNA allocation to gene upregulation.

## 6 Conclusion

In summary, we have demonstrated that chemically modified saRNAs that we have previously shown can upregulate the *STING* gene target (Section 4.2), are also capable of performing gene silencing, and act as siRNAs (Section 4.3). The same active guide strand can bind to a mRNA that we designed from a reporter plasmid system bearing this sequence. Our findings emphasize how saRNAs with stronger upregulation abilities tend to make for stronger gene knockdown abilities, whereas poor saRNA duplexes also make poor siRNA gene silencers. These data highlight how siRNA and saRNA, and their associated pathways of RNAi and RNAa, respectively, are related to each other, where both siRNA and saRNA's respective gene expression pathways must each first interact with AGO2. AGO2 localization is an important factor to be considered for better understanding of this relationship. It is clear that this unexplored relationship could be a key player to further understand the relationships between RNAi and RNAa. Ultimately, our goal will be to further optimize short RNA designs with chemical modifications to favor one pathway over another.

## Author Contributions

**Jean‐Paul** **Desaulniers, Troels Koch,** and **Henrik Hansen:** conceptualization. **Virginia Wing‐Nam** **Chiu, Matthew Lawrence Hammill, Marwah Elabed,** and **Yulia Lomonosova** **:** data curation. **Virginia Wing‐Nam Chiu:** formal analysis. **Troels Koch,** and **Jean‐Paul Desaulniers:** funding acquisition. **Virginia Wing‐Nam Chiu, Matthew Lawrence Hammill, Marwah Elabed, Sierra Varley, Joseph Hoare, Jon Voutila, Yulia Lomonosova, Jean‐Paul Desaulniers, Troels Koch,** and **Henrik Hansen**: investigation. **Joseph Hoare, Matthew Lawrence Hammill, Sierra Varley,** and **Virginia Wing‐Nam Chiu**: methodology. **Troels Koch,** and **Jean‐Paul Desaulniers:** project administration. **Troels Koch,** and **Jean‐Paul Desaulniers:** resources. **Yulia Lomonosova,** and **Joseph Hoare:** supervision. **Virginia Wing‐Nam Chiu, Matthew Lawrence Hammill, Sierra Varley, Joseph Hoare, Jon Voutila, Yulia Lomonosova, Jean‐Paul Desaulniers, Troels Koch,** and **Henrik Hansen:** validation. **Virginia Wing‐Nam Chiu,** and **Jon Voutila:** visualization. **Virginia Wing‐Nam Chiu:** writing – original draft. **Virginia Wing‐Nam Chiu, Jean‐Paul Desaulniers, Troels Koch, Henrik Hansen, Yulia Lomonosova, Jon Voutila,** and **Joseph Hoare:** writing – review and editing.

## Funding

This study was supported by Natural Sciences and Engineering Research Council of Canada (RGPIN‐2025‐05248).

## Conflicts of Interest

The authors declare no conflicts of interest.

## Supporting information

Supplementary Material

## Data Availability

The data that support the findings of this study are available from the corresponding author upon reasonable request.
