# Supplementary material for: Working So Hard: Double‐Stranded RNAs That Can Perform Both Gene Activation and Gene Silencing
Source: Chembiochem. 2026 Jun 30;27(13):e70434. doi: 10.1002/cbic.70434 (PMC13316730; doi:10.1002/cbic.70434)
Supplement: Supplementary file 1 — Supplementary Material [file CBIC-27-e70434-s001.pdf]

Working So Hard: Double-Stranded RNAs that Can Perform Both Gene Activation and Gene Silencing

Virginia Wing-Nam Chiu[a], Matthew Lawrence Hammill[a], Marwah Elabed[a], Sierra Varley[a], Yulia Lomonosova[b] Joseph Hoare[b], Jon Voutila[b], Henrik Hansen[b], Troels Koch[b], and Jean-Paul Desaulniers[a*]

[a] Faculty of Science, Ontario Tech University, 2000 Simcoe Street North, Oshawa Ontario L1G 0C5
[b] MiNA Therapeutics, 84 Wood Lane, London, United Kingdom W12 0BZ

Figures

Figure S-1: CD spectra of modified saRNA Wildtype S2
Figure S-2: CD spectra of modified saRNAs STING-1/WTFA-1 and WTFA-1/STING-2 ....S2
Figure S-3: CD spectra of modified saRNAs WTFA-1 and WTFA-2, LWTFA-23, MEFX-2 ....S3
Figure S-4: CD spectra of modified saRNAs CWTFA-2 and Wildtype, WTFA-2, LWTFX-21 ....S3
Figure S-5: CD spectra of modified saRNAs CWTFA-2 and LWTFA-23, PS-10-LWTFA-23, MEFX-2....S4
Figure S-6: CD spectra of modified saRNAs CWTFA-X, UWTFA-X, and MEFC-2 ....S4
Figure S-7: CD spectra of modified saRNAs UWTFA-3 and WTFA-2, LWTFA-23, MEFA-2 ....S5
Figure S-8: CD spectra of modified saRNAs SCR and mismatches part 1 ....S5
Figure S-9: CD spectra of modified saRNAs SCR and mismatches part 2 ....S6
Figure S-10: CD spectra of modified saRNA SCR CWTFA-2/SCR STING-2 ....S6
Figure S-11: Drug response curve of modified saRNAs Wildtype, CWTFA-2 and WTFA-2, LWTFA-23, MEFA-2 ....S7
Figure S-12: Drug response curve of modified saRNAs CWTFA-2 and (PS-10)LWTFX-2X S7
Figure S-13: Drug response curve of modified saRNAs UWTFA-3 and WTFA-2, MEFA-2, LWTFA-23....S8
Figure S-14: Drug response curve of modified saRNAs WTFA-1 and WTFA-2, LWTFA-23 ....S8
Figure S-15: Drug response curve of modified saRNAs (C)WTFA-X and MEFC-2 ....S9
Figure S-16: Drug response curve of modified saRNA WTFA-1 and MEFA-2 ....S9
Figure S-17: Drug response curve of UWTFA-X and MEFC-2 ....S10
Figure S-18: Drug response curve of STING-1, WTFA-1, CWTFA-2 and STING-2, WTFA-2 ....S10
Figure S-19: Drug response curve of mismatch and scramble control saRNAs S11
Figure S-20: UV melting temperature curves of modified saRNAs CWTFA-2, LWTFU-21, LWTFG-21, PS-10-LWTFA-23, MEFA-2 and MEFC-2 ....S11
Figure S-21: UV melting temperature curves of WTFA-1, MEFA-2 and MEFC-2 ....S12
Figure S-22: UV melting temperature curves of CWTFA- (X), MEFC-2, and UWTFA-3/MEFA-2....S12
Figure S-23: UV melting temperature curves OF UWTFA-(X) and MEFC-2 ....S13
Figure S-24: HPLC chromatogram of MEFA-2 ....S13
Figure S-25: HPLC chromatogram of MEFC-2 ....S14
Figure S-26: HPLC chromatogram of LWTFU-21 ....S14
Figure S-27: HPLC chromatogram of LWTFG-21 ....S15
Figure S-28: HPLC chromatogram of PS-10-LWTFA-23 ....S15
Table S-1: Table of chemically modified RNA sequences and observed masses S16
Figure S-29: Gene silencing of saRNAs STING-1/STING-2, CWTFA-2/LWTFA-23, CWTFA-2/MEFA-2, WTFA-1/WTFA-2, SCR STING-1/SCR STING-2, and SCR CWTFA-2/SCR LWTFA-23 in A549 cells S16
Figure S-30: Plasmid map of pGL3-*STING* S17
Figure S-31: Enlarged image of pGL3-STING region of pGL3-*STING* plasmid map S17
Figure S-32: pGL3-*STING* plasmid sequencing result of *STING* insert location S18

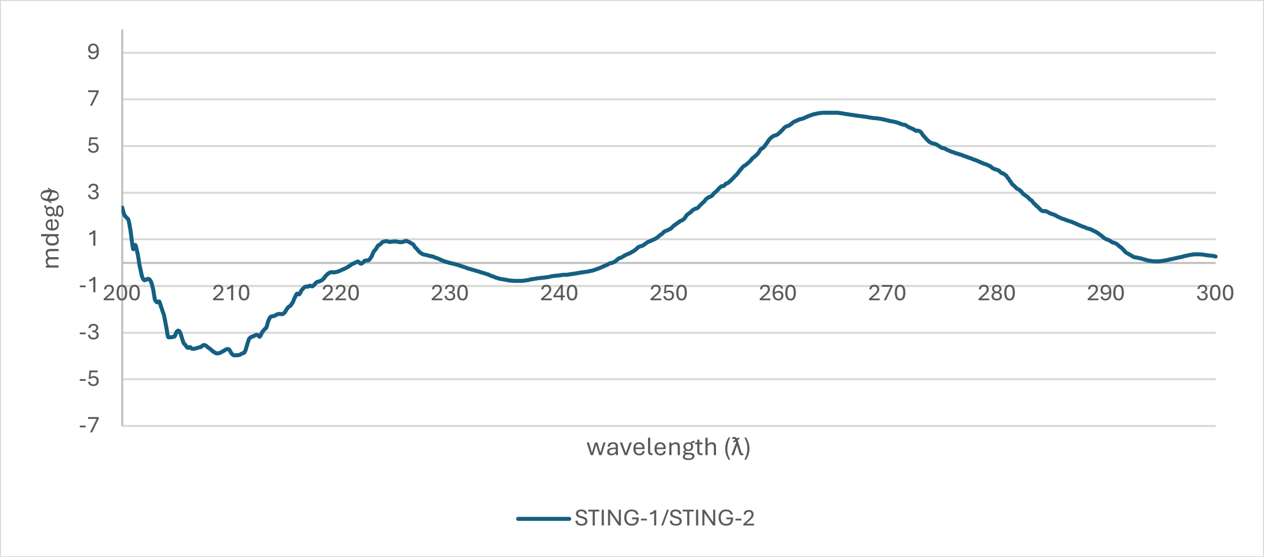

Figure S-1. CD spectra of wildtype STING-1/STING-2 saRNA (10 µM/duplex). Duplex was suspended in 500 µL of a sodium phosphate buffer (90.0 mM NaCl, 10.0 mM Na_2HPO_4, 1.00 mM EDTA, pH 7.00) and scanned from 200-350 nm at 15 °C with a screening rate of 20.0 nm/min and a 0.20 nm data pitch. All scans were performed in triplicate and averaged using Jasco's Spectra Manager version 2. CD spectra of some saRNA duplexes are taken from data obtained in a previous publication but included in these figures for ease of comparison (11).

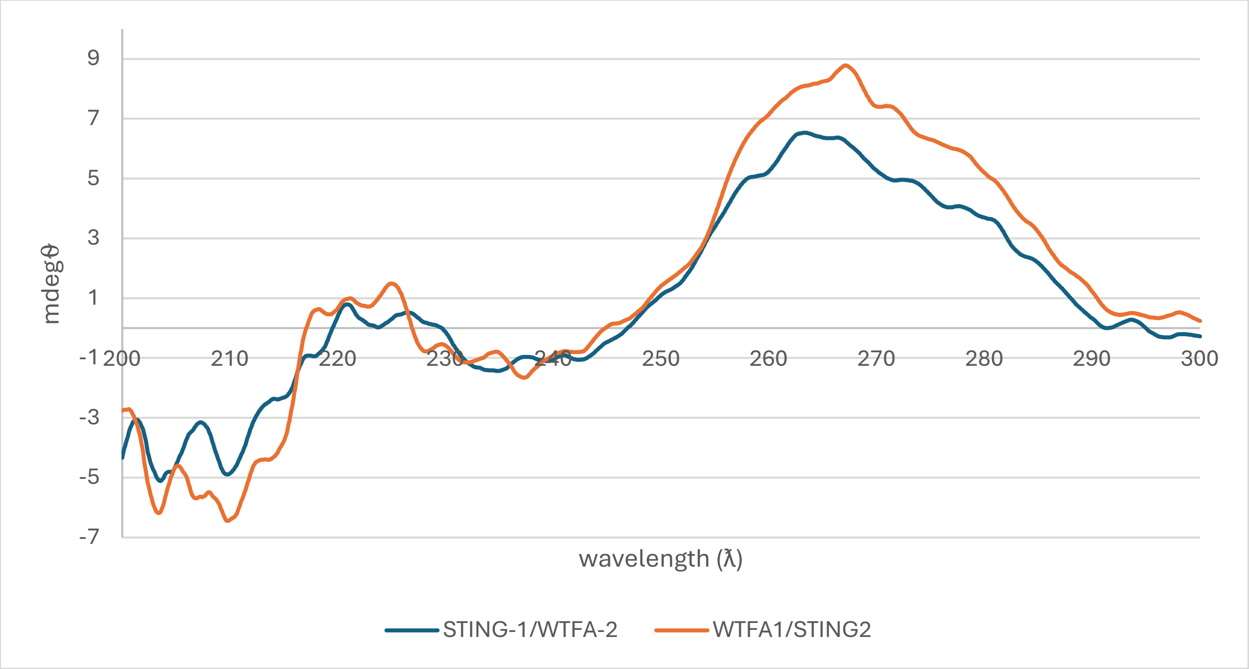

Figure S-2. CD spectra of STING-1/WTFA-1 and WTFA-1/STING-2 anti-*STING* saRNAs (10 µM/duplex). Duplexes were suspended in 500 µL of a sodium phosphate buffer (90.0 mM NaCl, 10.0 mM Na_2HPO_4, 1.00 mM EDTA, pH 7.00) and scanned from 200-350 nm at 15 °C with a screening rate of 20.0 nm/min and a 0.20 nm data pitch. All scans were performed in triplicate and averaged using Jasco's Spectra Manager version 2. CD spectra of some saRNA duplexes are taken from data obtained in a previous publication but included in these figures for ease of comparison (11).

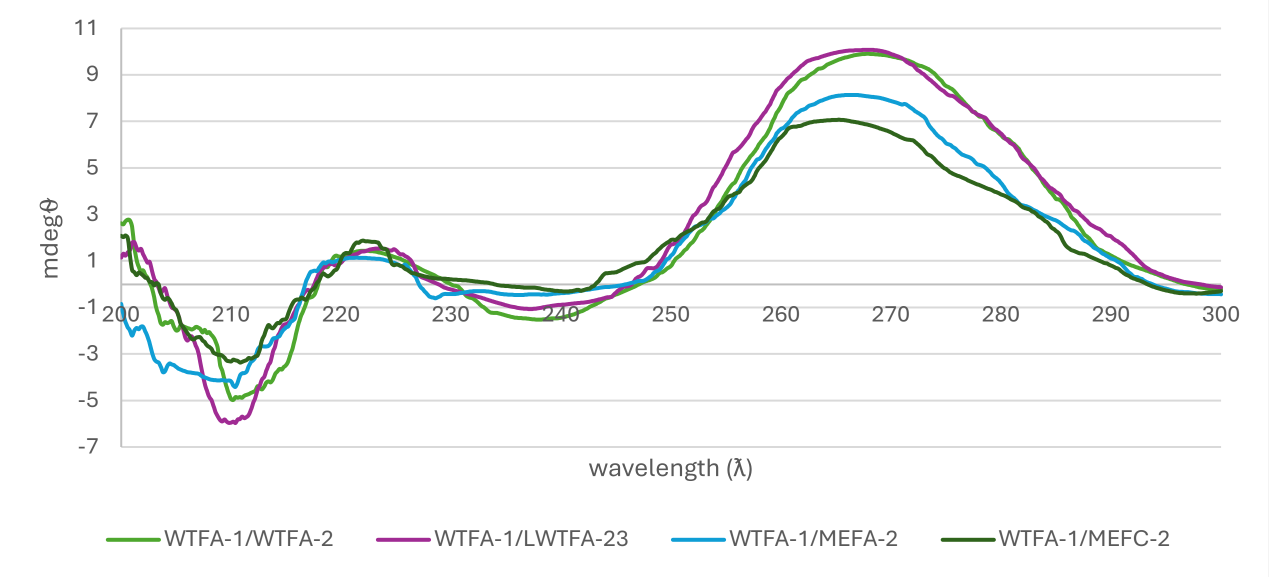

Figure S-3. CD spectra of WTFA-1 and WTFA-2, LWTFA-23, MEFX-2 modified anti-*STING* saRNAs (10 µM/duplex). Duplexes were suspended in 500 µL of a sodium phosphate buffer (90.0 mM NaCl, 10.0 mM Na_2HPO_4, 1.00 mM EDTA, pH 7.00) and scanned from 200-350 nm at 15 °C with a screening rate of 20.0 nm/min and a 0.20 nm data pitch. All scans were performed in triplicate and averaged using Jasco's Spectra Manager version 2. CD spectra of some saRNA duplexes are taken from data obtained in a previous publication but included in these figures for ease of comparison (11).

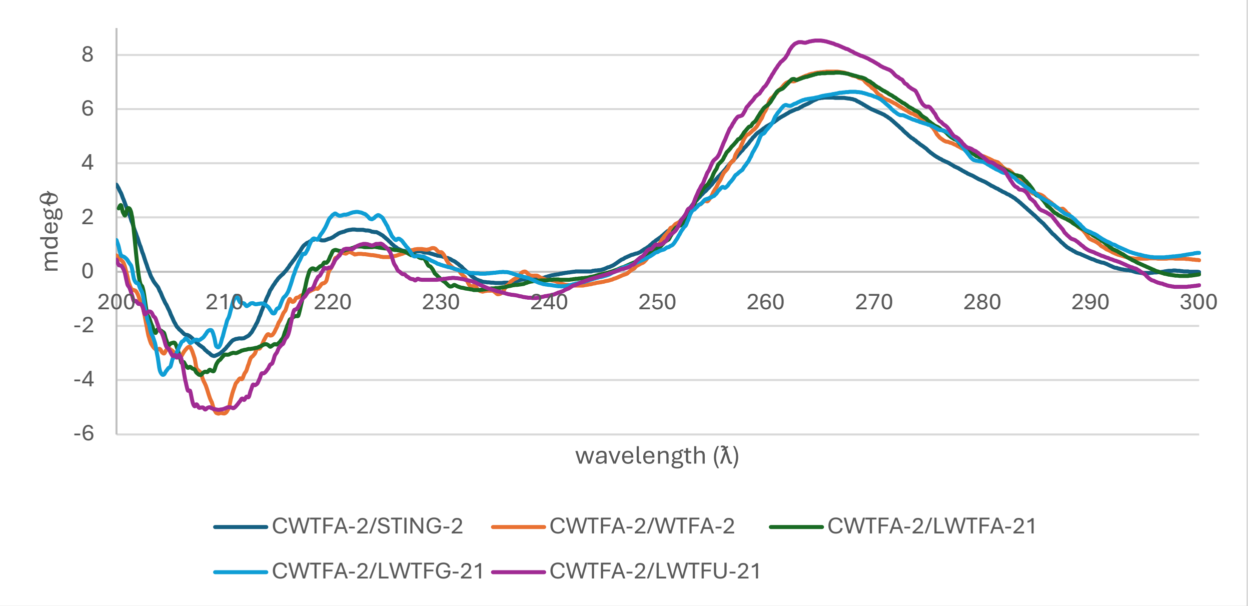

Figure S-4. CD spectra of CWTFA-2 annealed to STING-2, WTFA-2, LWTFX-21 modified anti-*STING* saRNAs (10 µM/duplex). Duplexes were suspended in 500 µL of a sodium phosphate buffer (90.0 mM NaCl, 10.0 mM Na_2HPO_4, 1.00 mM EDTA, pH 7.00) and scanned from 200-350 nm at 15 °C with a screening rate of 20.0 nm/min and a 0.20 nm data pitch. All scans were performed in triplicate and averaged using Jasco's Spectra Manager version 2. CD spectra of some saRNA duplexes are taken from data obtained in a previous publication but included in these figures for ease of comparison (11).

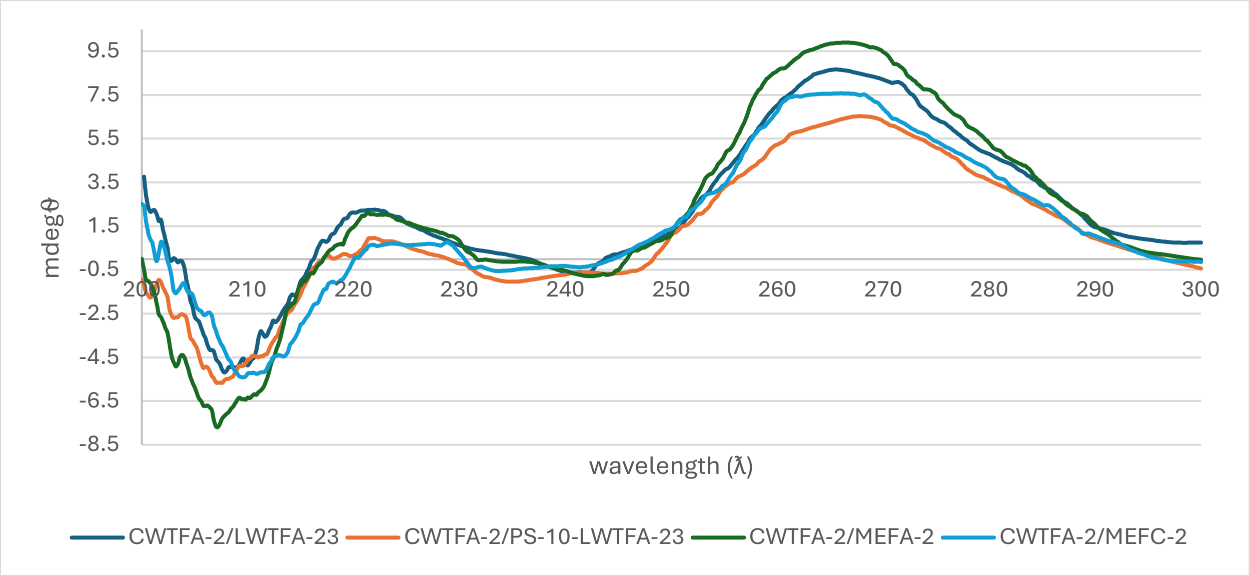

Figure S-5. CD spectra of CWTFA-2 and LWTFA-23, PS-10-LWTFA-23, MEFX-2 modified anti-*STING* saRNAs (10 µM/duplex). Duplexes were suspended in 500 µL of a sodium phosphate buffer (90.0 mM NaCl, 10.0 mM Na_2HPO_4, 1.00 mM EDTA, pH 7.00) and scanned from 200-350 nm at 15 °C with a screening rate of 20.0 nm/min and a 0.20 nm data pitch. All scans were performed in triplicate and averaged using Jasco's Spectra Manager version 2. CD spectra of some saRNA duplexes are taken from data obtained in a previous publication but included in these figures for ease of comparison (11).

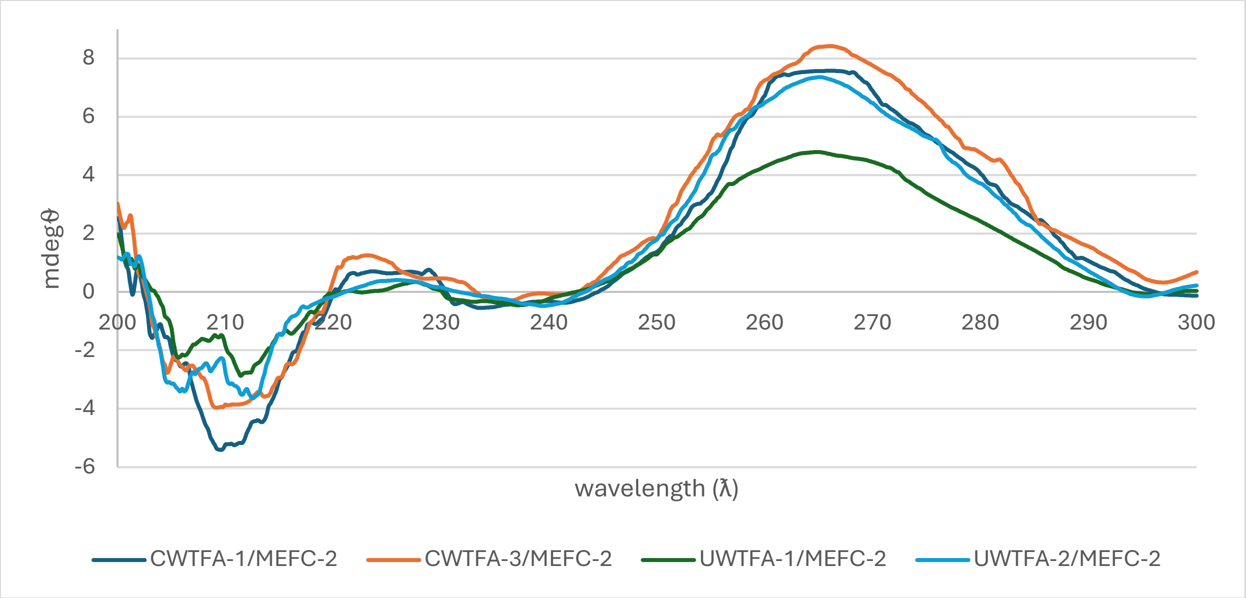

Figure S-6. CD spectra of CWTFA-X, UWTFA-X, and MEFC-2 modified anti-*STING* saRNAs (10 µM/duplex). Duplexes were suspended in 500 µL of a sodium phosphate buffer (90.0 mM NaCl, 10.0 mM Na_2HPO_4, 1.00 mM EDTA, pH 7.00) and scanned from 200-350 nm at 15 °C with a screening rate of 20.0 nm/min and a 0.20 nm data pitch. All scans were performed in triplicate and averaged using Jasco's Spectra Manager version 2. CD spectra of some saRNA duplexes are taken from data obtained in a previous publication but included in these figures for ease of comparison (11).

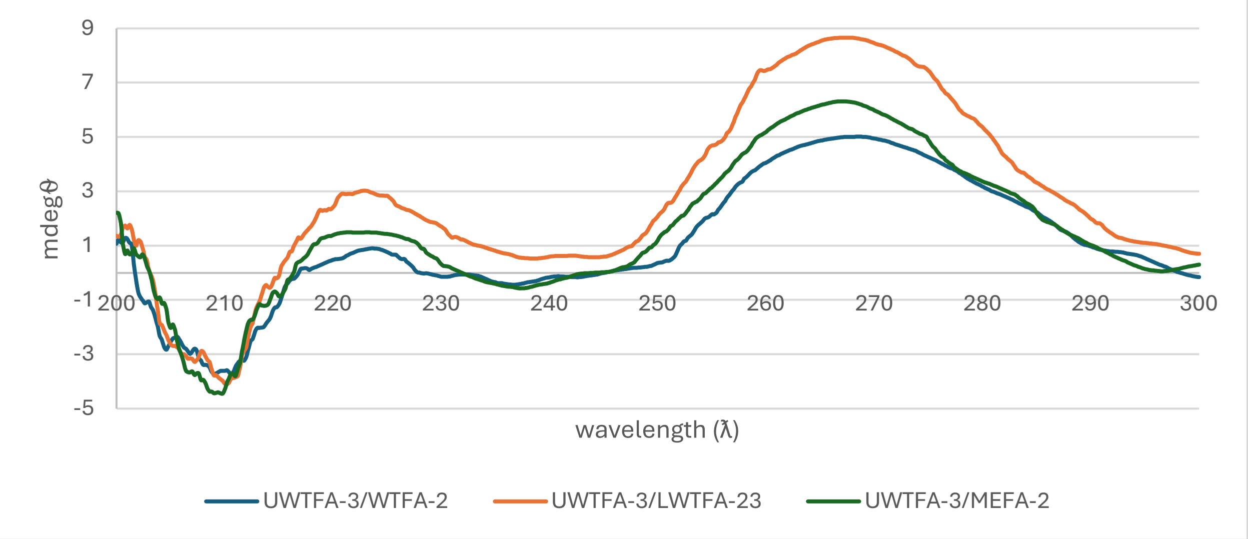

Figure S-7. CD spectra of UWTFA-3 and WTFA-2, LWTFA-23, MEFA-2 modified anti-*STING* saRNAs (10 µM/duplex). Duplexes were suspended in 500 µL of a sodium phosphate buffer (90.0 mM NaCl, 10.0 mM Na_2HPO_4, 1.00 mM EDTA, pH 7.00) and scanned from 200-350 nm at 15 °C with a screening rate of 20.0 nm/min and a 0.20 nm data pitch. All scans were performed in triplicate and averaged using Jasco's Spectra Manager version 2. CD spectra of some saRNA duplexes are taken from data obtained in a previous publication but included in these figures for ease of comparison (11).

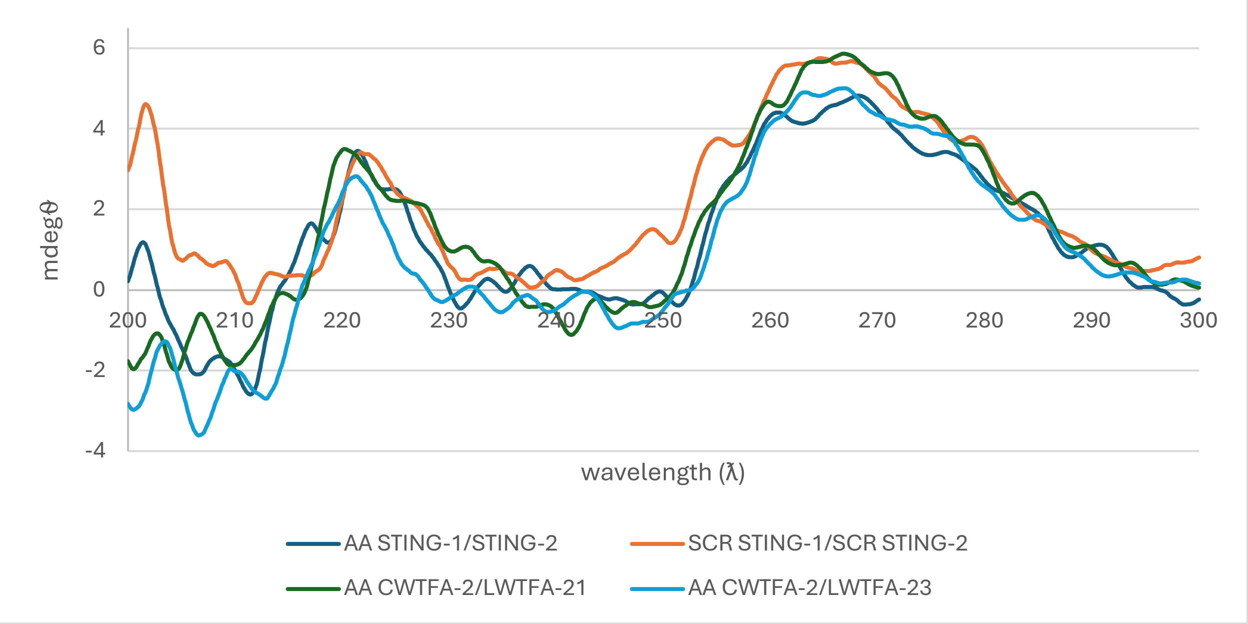

Figure S-8. CD spectra of SCR and mismatches part 1, AA STING-1/STING-2. AA CWTFA-2/LWTFA-21, SCR STING-1/SCR STING-2, and AA CWTFA-2/LWTFA-23 (10 µM/duplex). Duplexes were suspended in 500 µL of a sodium phosphate buffer (90.0 mM NaCl, 10.0 mM Na_2HPO_4, 1.00 mM EDTA, pH 7.00) and scanned from 200-350 nm at 15 °C with a screening rate of 20.0 nm/min and a 0.20 nm data pitch. All scans were performed in triplicate and averaged using Jasco's Spectra Manager version 2. CD spectra of some saRNA duplexes are taken from data obtained in a previous publication but included in these figures for ease of comparison (11).

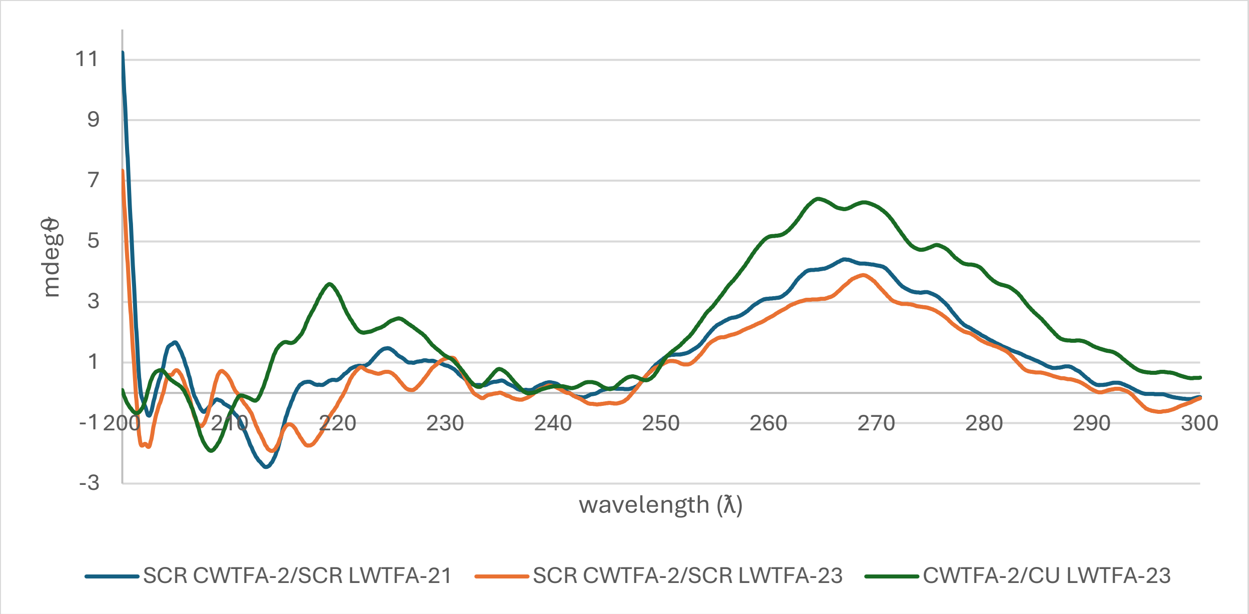

Figure S-9. CD spectra of modified saRNAs targeting *STING* mRNAs. SCR and mismatches part 2, SCR CWTFA-2/SCR LWTFA-21, SCR CWTFA-2/SCR LWTFA-23, CWTFA-2/CU LWTFA-23 (10 µM/duplex). Duplexes were suspended in 500 µL of a sodium phosphate buffer (90.0 mM NaCl, 10.0 mM Na_2HPO_4, 1.00 mM EDTA, pH 7.00) and scanned from 200-350 nm at 15 °C with a screening rate of 20.0 nm/min and a 0.20 nm data pitch. All scans were performed in triplicate and averaged using Jasco's Spectra Manager version 2. CD spectra of some saRNA duplexes are taken from data obtained in a previous publication but included in these figures for ease of comparison (11).

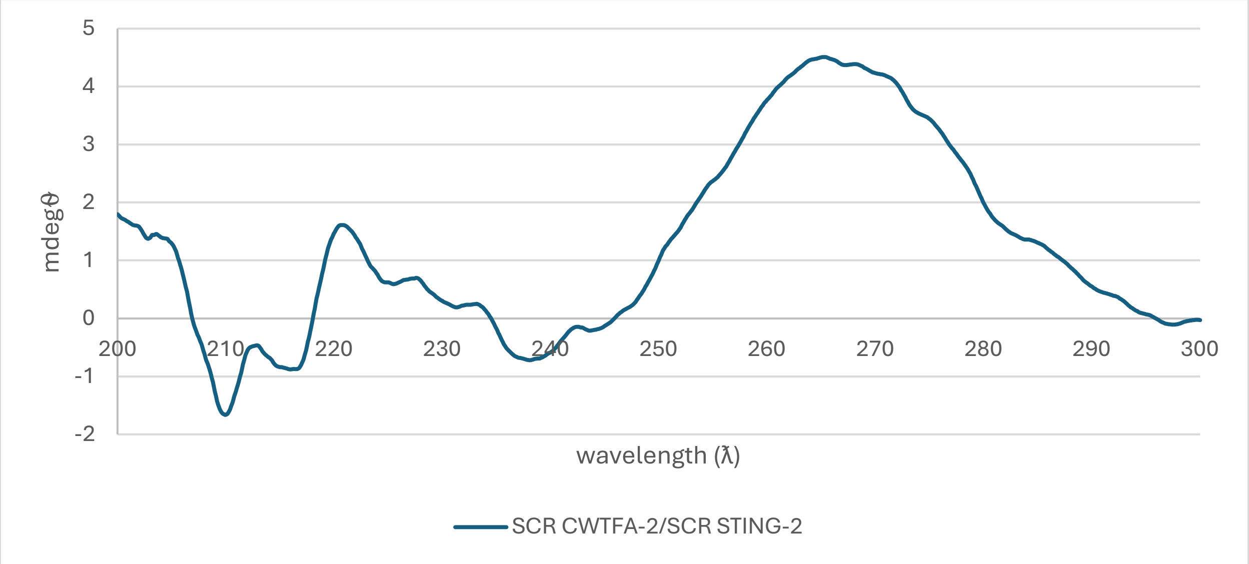

Figure S-10. CD spectra of SCR CWTFA-2/SCR STING-2 (10 µM/duplex). Duplex was suspended in 500 µL of a sodium phosphate buffer (90.0 mM NaCl, 10.0 mM Na_2HPO_4, 1.00 mM EDTA, pH 7.00) and scanned from 200-350 nm at 15 °C with a screening rate of 20.0 nm/min and a 0.20 nm data pitch. All scans were performed in triplicate and averaged using Jasco's Spectra Manager version 2. CD spectra of some saRNA duplexes are taken from data obtained in a previous publication, but included in these figures for ease of comparison (11).

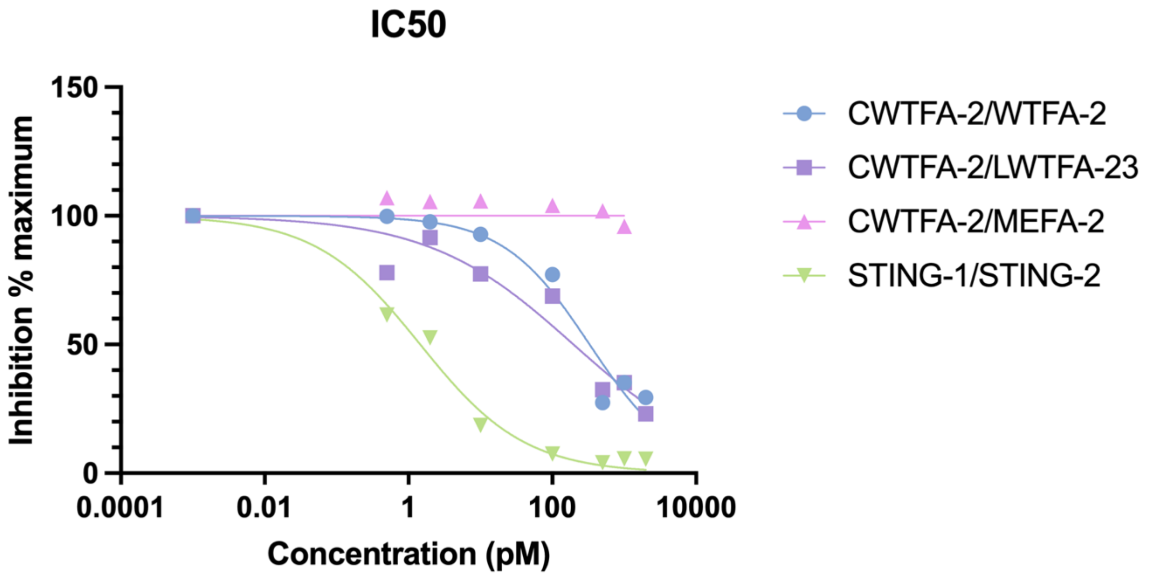

Figure S-11. Drug response curve of modified saRNAs targeting STING mRNA. CWTFA-2 and WTFA-2, LWTFA-23, MEFA-2 modified anti-*STING* saRNAs. Duplexes (4 nM to 1 pM) were transfected into HeLa cells and incubated 24 hours before cell lysis and subjection to a dual luciferase assay, with mean % relative luciferase expression obtained from minimum n=2. All IC50 values were obtained using the nonlinear regression model in GraphPad Prism version 10.4.1.

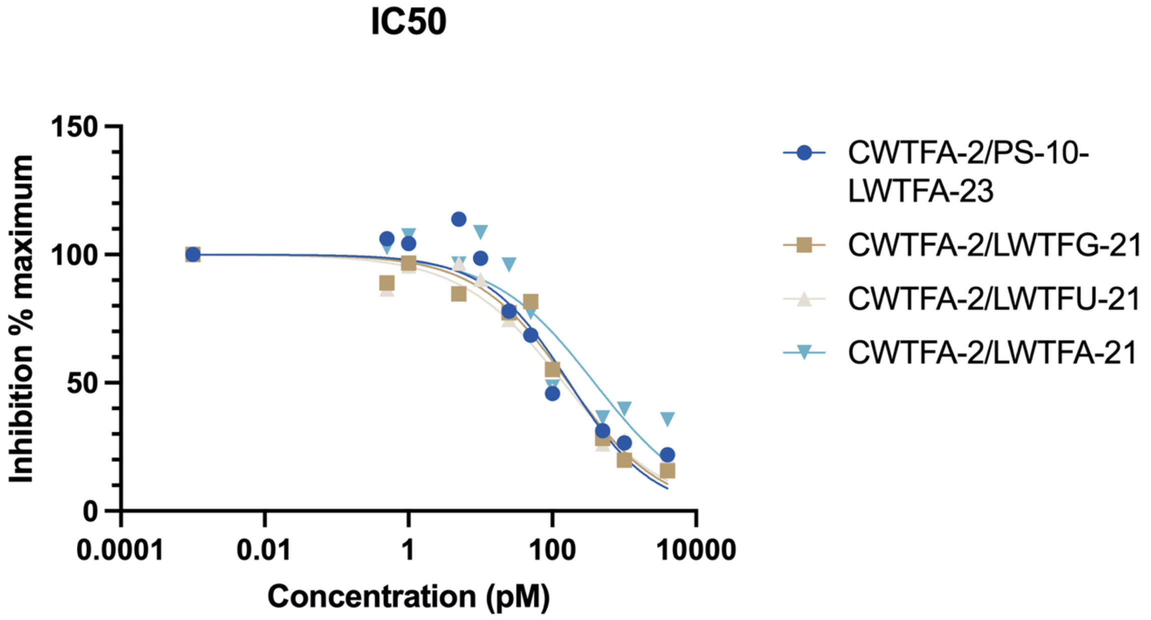

Figure S-12. Drug response curve of modified saRNAs targeting STING mRNA. CWTFA-2 and (PS-10)LWTFX-21 modified anti-*STING* saRNAs. Duplexes (4 nM to 1 pM) were transfected into HeLa cells and incubated 24 hours before cell lysis and subjection to a dual luciferase assay, with mean % relative luciferase expression obtained from minimum n=2. All IC50 values were obtained using the nonlinear regression model in GraphPad Prism version 10.4.1.

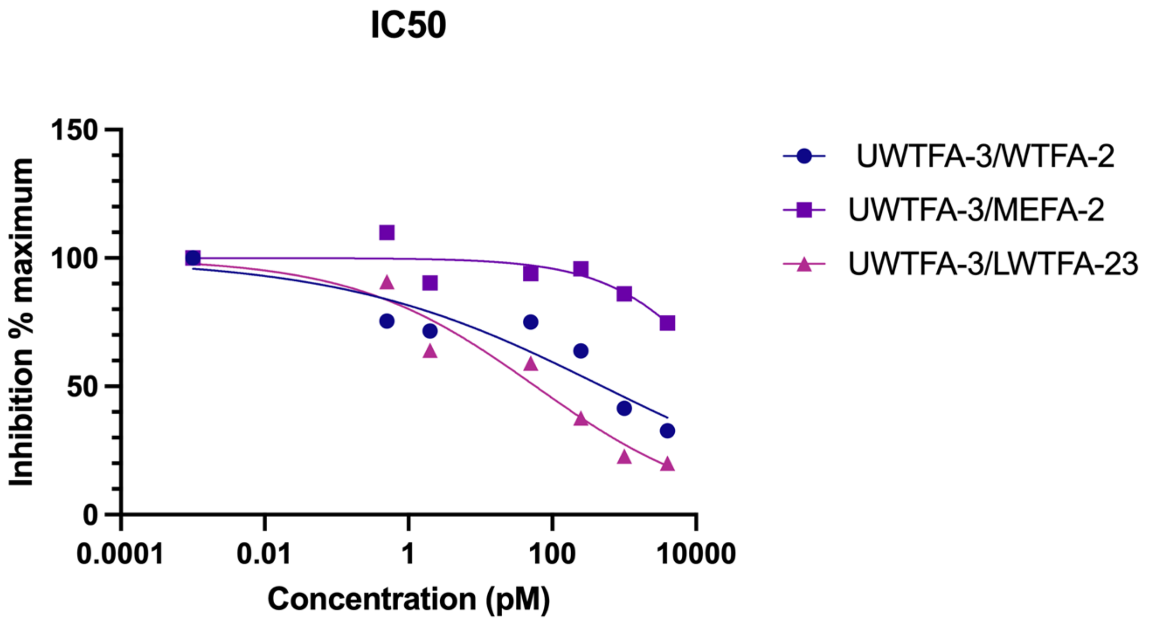

Figure S-13. Drug response curve of modified saRNAs targeting STING mRNA. UWTFA-3 and WTFA-2, LWTFA-23, MEFA-2 modified anti-*STING* saRNAs. Duplexes (4 nM to 1 pM) were transfected into HeLa cells and incubated 24 hours before cell lysis and subjection to a dual luciferase assay, with mean % relative luciferase expression obtained from minimum n=2. All IC50 values were obtained using the nonlinear regression model in GraphPad Prism version 10.4.1.

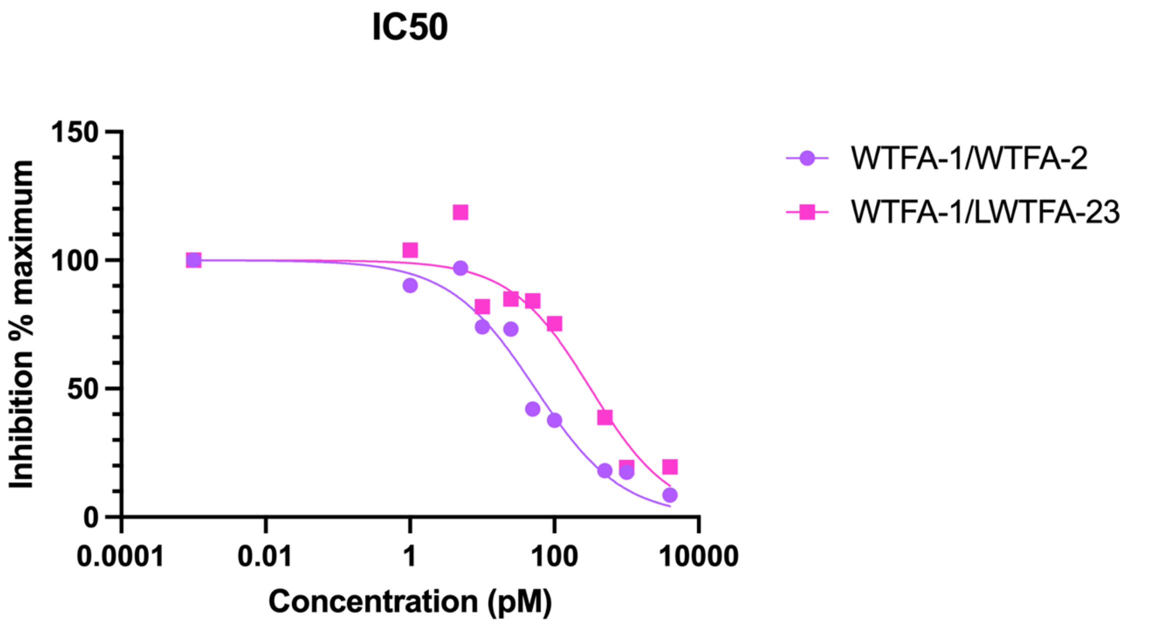

Figure S-14. Drug response curve of modified saRNAs targeting STING mRNA. WTFA-1 and WTFA-2, LWTFA-23 modified anti-*STING* saRNAs. Duplexes (4 nM to 1 pM) were transfected into HeLa cells and incubated 24 hours before cell lysis and subjection to a dual luciferase assay, with mean % relative luciferase expression obtained from minimum n=2. All IC50 values were obtained using the nonlinear regression model in GraphPad Prism version 10.4.1.

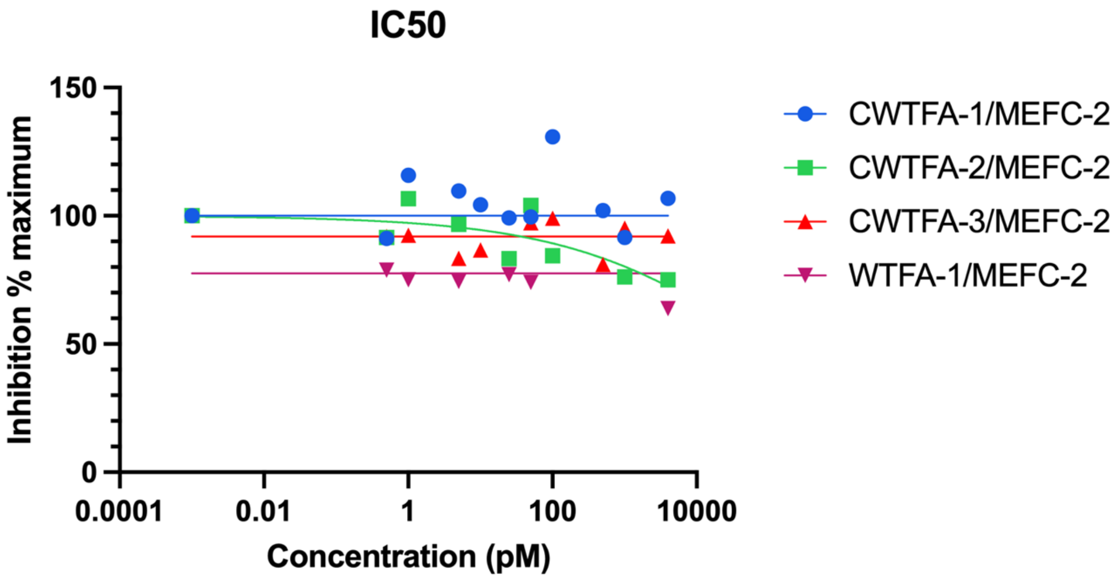

Figure S-15. Drug response curve of modified saRNAs targeting STING mRNA. (C)WTFA-X and MEFC-2modified anti-*STING* saRNAs. Duplexes (4 nM to 1 pM) were transfected into HeLa cells and incubated 24 hours before cell lysis and subjection to a dual luciferase assay, with mean % relative luciferase expression obtained from minimum n=2. All IC50 values were obtained using the nonlinear regression model in GraphPad Prism version 10.4.1.

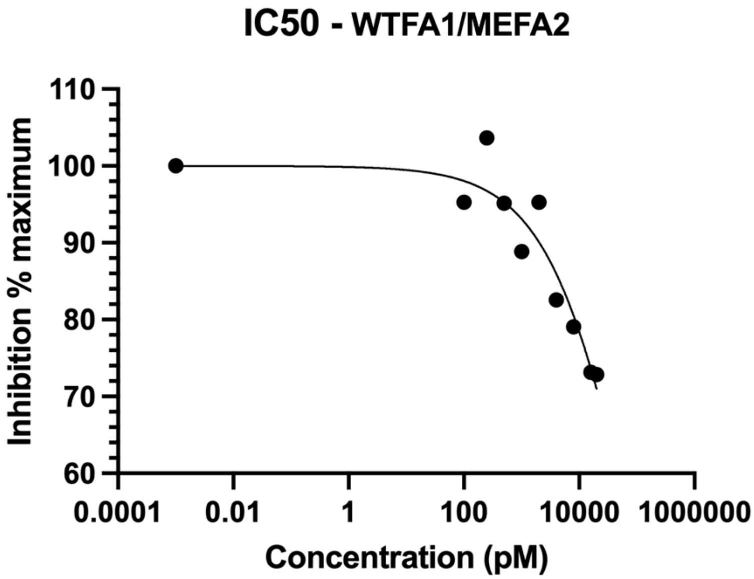

Figure S-16. Drug response curve of modified saRNA targeting STING mRNA. WTFA-1 and MEFA-2 modified anti-*STING* saRNA. Duplex (20 nM to 100 pM) was transfected into HeLa cells and incubated 24 hours before cell lysis and subjection to a dual luciferase assay, with mean % relative luciferase expression obtained from minimum n=2. All IC50 values were obtained using the nonlinear regression model in GraphPad Prism version 10.4.1.

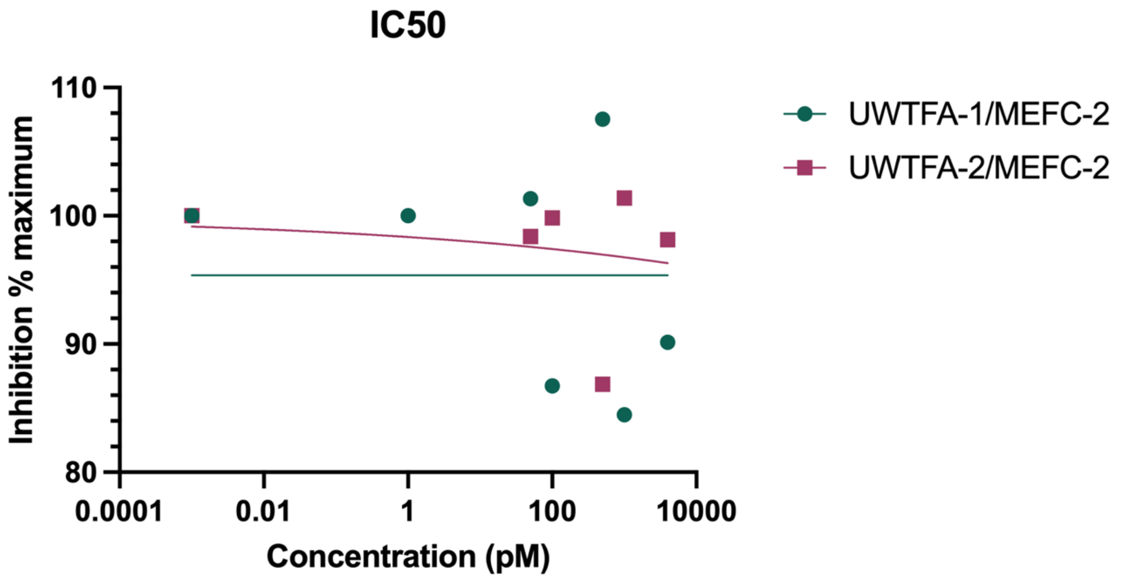

Figure S-17. Drug response curve of modified saRNAs targeting STING mRNA. UWTFA-X and MEFC-2 modified anti-*STING* saRNAs. Duplexes (4 nM to 1 pM) were transfected into HeLa cells and incubated 24 hours before cell lysis and subjection to a dual luciferase assay, with mean % relative luciferase expression obtained from minimum n=2. All IC50 values were obtained using the nonlinear regression model in GraphPad Prism version 10.4.1.

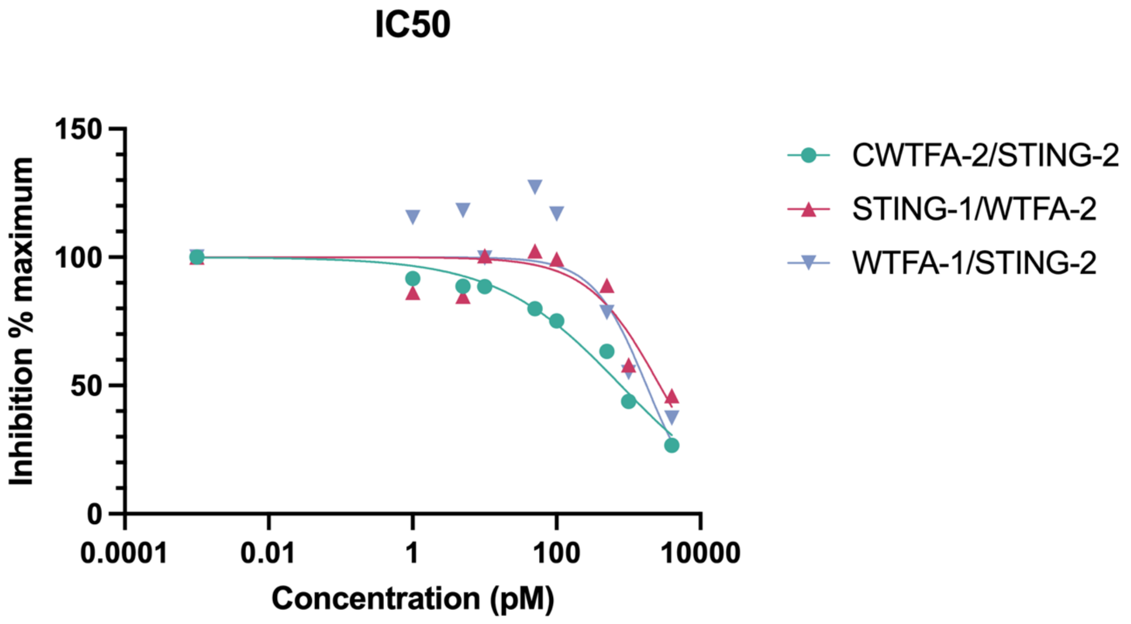

Figure S-18. Drug response curve of modified saRNAs targeting STING mRNA. STING-1, CWTFA-2, WTFA-1 and WTFA-2 and STING-2 modified anti-*STING* saRNAs. Duplexes (4 nM to 1 pM) were transfected into HeLa cells and incubated 24 hours before cell lysis and subjection to a dual luciferase assay, with mean % relative luciferase expression obtained from minimum n=2. All IC50 values were obtained using the nonlinear regression model in GraphPad Prism version 10.4.1.

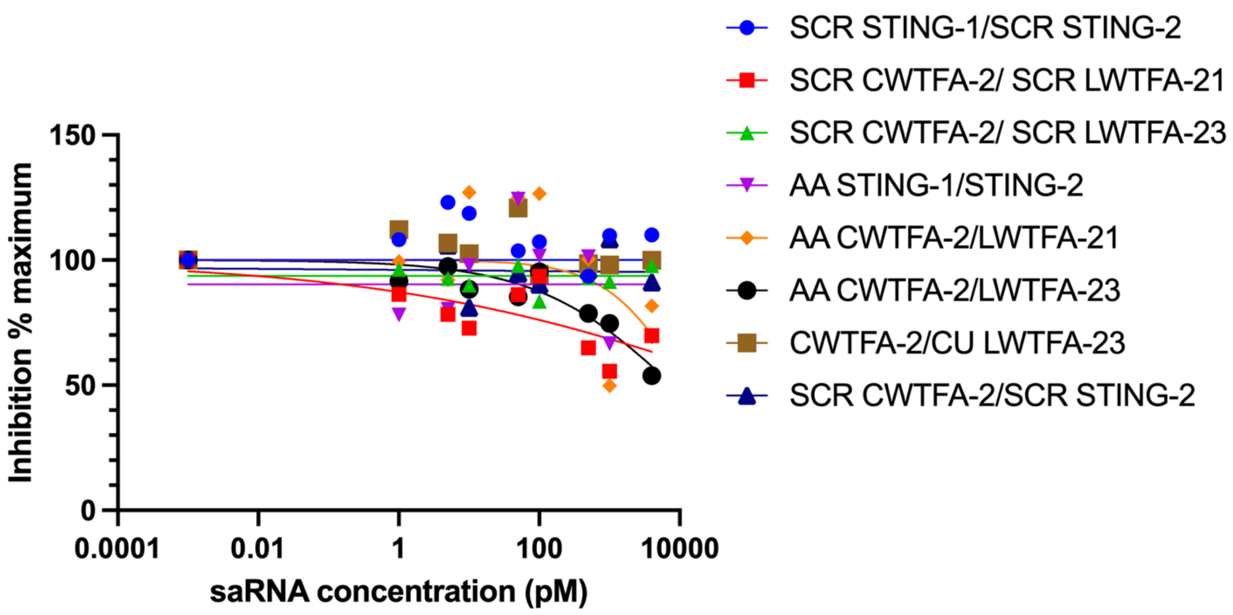

Figure S-19. Drug response curve of modified scrambled and mismatch control saRNAs targeting STING mRNA. Duplexes (4 nM to 1 pM) were transfected into HeLa cells and incubated 24 hours before cell lysis and subjection to a dual luciferase assay, with mean % relative luciferase expression obtained from minimum n=2. All IC50 values were obtained using the nonlinear regression model in GraphPad Prism version 10.4.1.

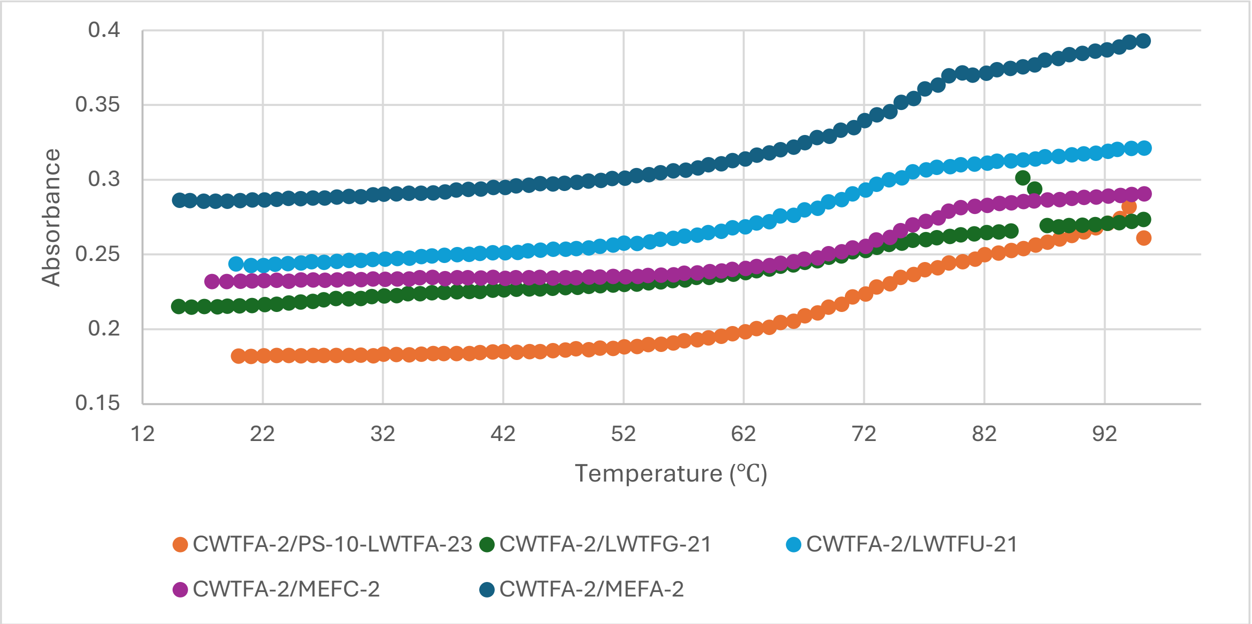

Figure S-20. Melt curves of modified saRNAs targeting *STING* mRNAs. CWTFA-2 and LWTFU-21, LWTFG-21, PS-10-LWTFA-23, MEFA-2 and MEFC-2. 10 µM/duplex were suspended in 500 µL of a sodium phosphate buffer (90.0 mM NaCl, 10.0 mM Na2HPO4, 1.00 mM EDTA, pH 7.00) and scanned from 15-95 °C with a ramp rate of 1 °C/min, with absorbance being measured once per 1 °C. All melts were performed in duplicate or triplicate and averaged using Jasco's Spectra Manager version 2 or in Microsoft excel. Tm values were determined using Meltwin v3.5 software assuming a 2 state model.

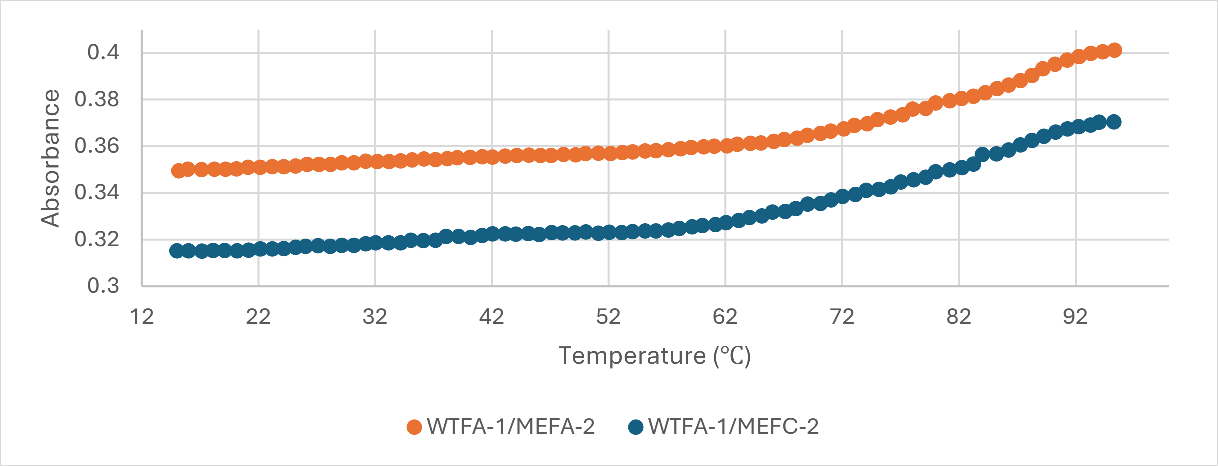

Figure S-21. Melt curves of modified saRNAs targeting *STING* mRNAs. WTFA-1, MEFA-2 and MEFC-2. 10 µM/duplex were suspended in 500 µL of a sodium phosphate buffer (90.0 mM NaCl, 10.0 mM Na2HPO4, 1.00 mM EDTA, pH 7.00) and scanned from 15-95 °C with a ramp rate of 1 °C/min, with absorbance being measured once per 1 °C. All melts were performed in duplicate or triplicate and averaged using Jasco's Spectra Manager version 2 or in Microsoft excel. Tm values were determined using Meltwin v3.5 software assuming a 2 state model.

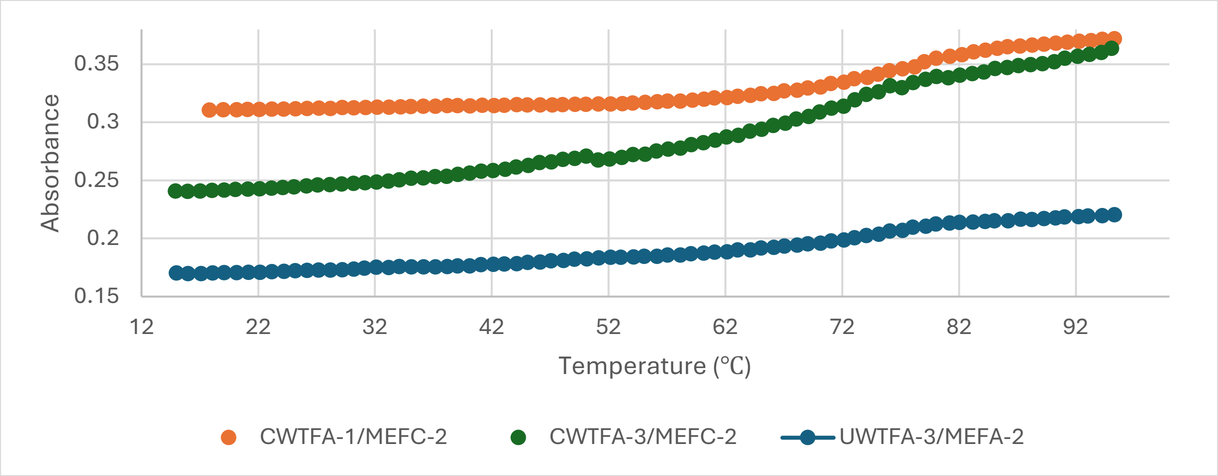

Figure S-22. Melt curves of modified saRNAs targeting *STING* mRNAs. (C/U)WTFA(X), MEFA-2 and MEFC-2. 10 µM/duplex were suspended in 500 µL of a sodium phosphate buffer (90.0 mM NaCl, 10.0 mM Na2HPO4, 1.00 mM EDTA, pH 7.00) and scanned from 15-95 °C with a ramp rate of 1 °C/min, with absorbance being measured once per 1 °C. All melts were performed in duplicate or triplicate and averaged using Jasco's Spectra Manager version 2 or in Microsoft excel. Tm values were determined using Meltwin v3.5 software assuming a 2 state model.

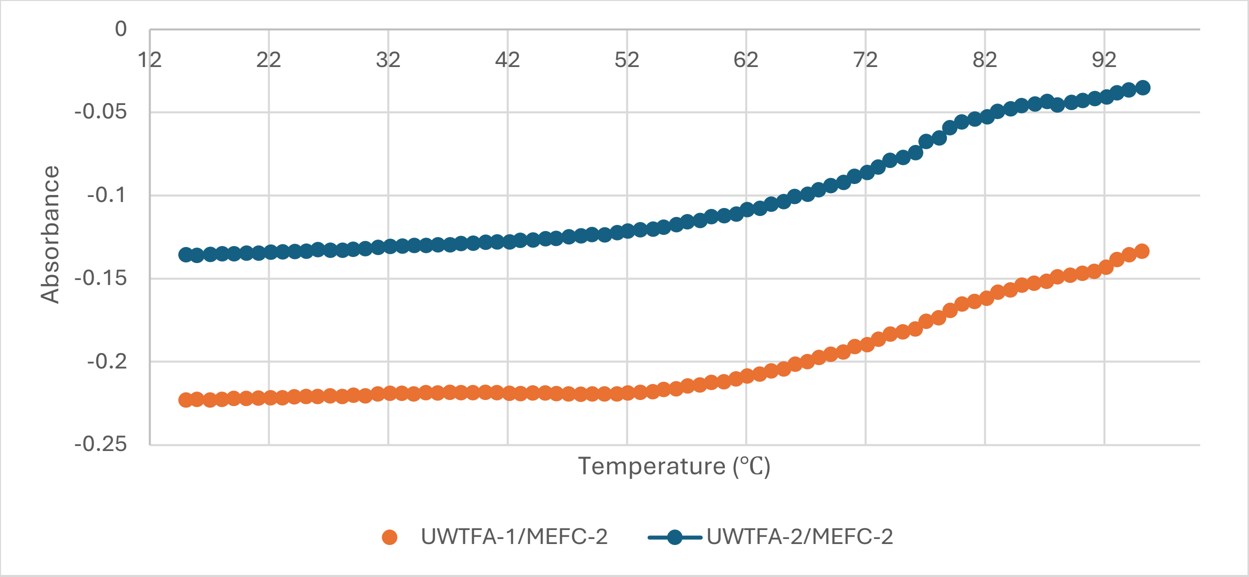

Figure S-23. Melt curves of modified saRNAs targeting *STING* mRNAs. (C/U)WTFA(X), MEFA-2 and MEFC-2. 10 µM/duplex were suspended in 500 µL of a sodium phosphate buffer (90.0 mM NaCl, 10.0 mM Na2HPO4, 1.00 mM EDTA, pH 7.00) and scanned from 15-95 °C with a ramp rate of 1 °C/min, with absorbance being measured once per 1 °C. All melts were performed in duplicate or triplicate and averaged using Jasco's Spectra Manager version 2 or in Microsoft excel. Tm values were determined using Meltwin v3.5 software assuming a 2 state model.

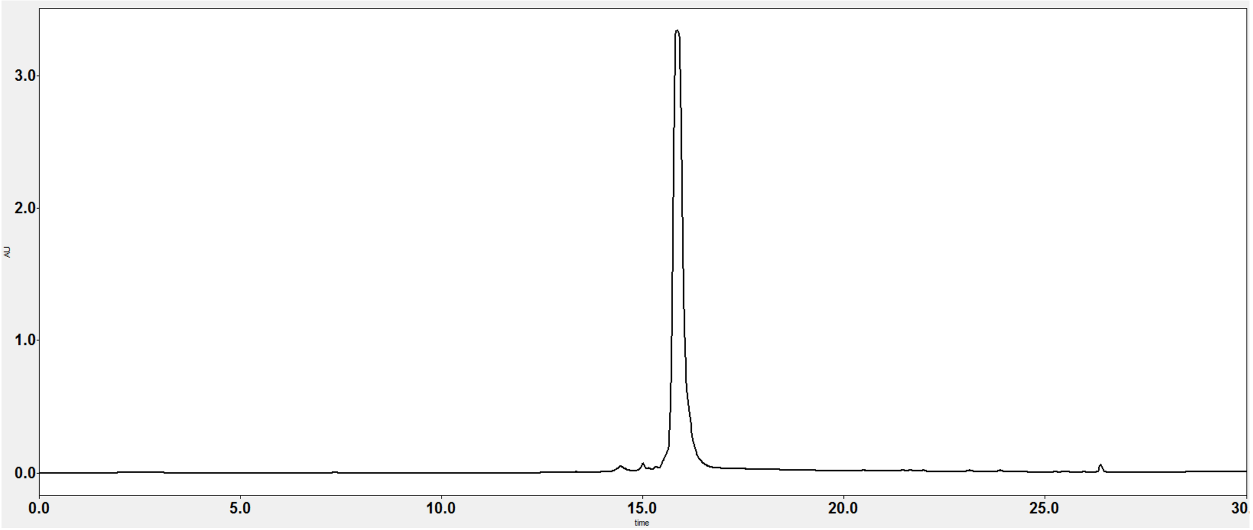

Figure S-24. HPLC chromatogram of sense strand MEFA-2. Conditions were 5% acetonitrile in 95% 0.1 M TEAA (Triethylamine-Acetic Acid) buffer up to 100% acetonitrile over 30 min. Spectra were processed using the Empower 3 software.

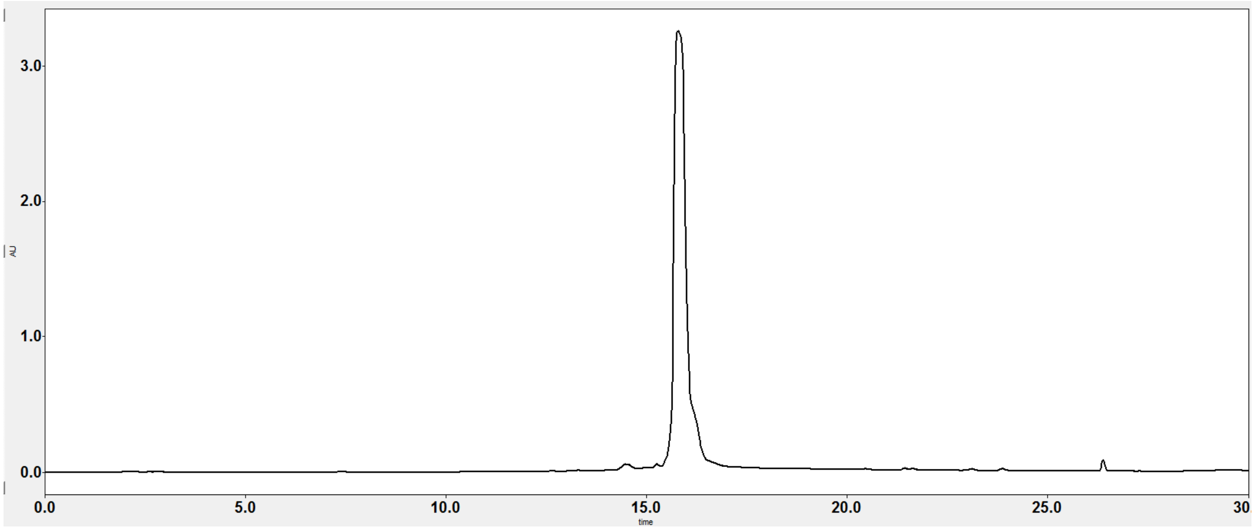

Figure S-25. HPLC chromatogram of sense strand MEFC-2. Conditions were 5% acetonitrile in 95% 0.1 M TEAA (Triethylamine-Acetic Acid) buffer up to 100% acetonitrile over 30 min. Spectra were processed using the Empower 3 software.

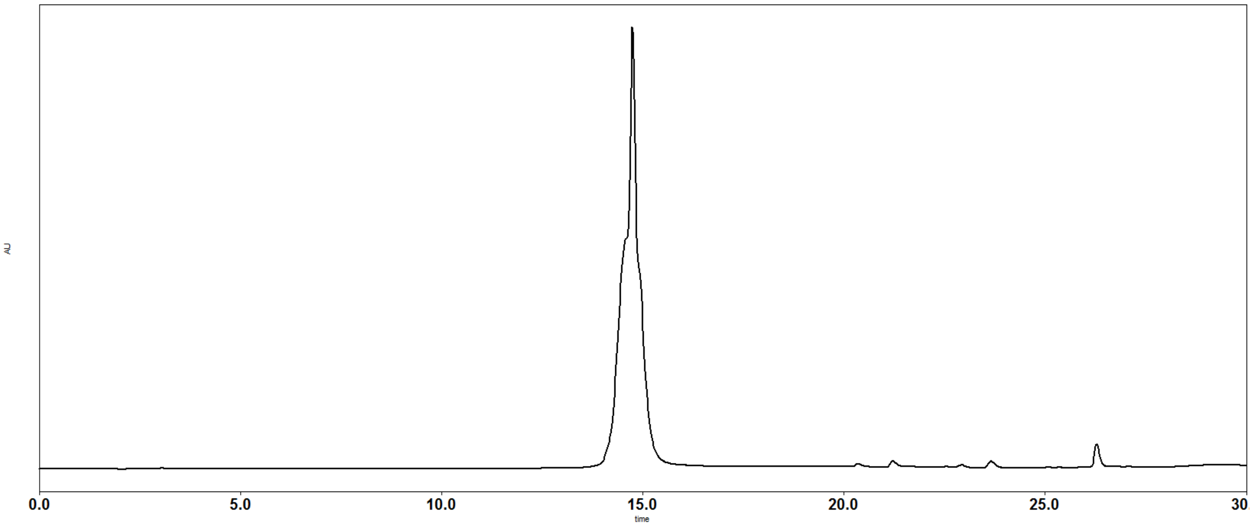

Figure S-26. HPLC chromatogram of sense strand LWTFU-21. Conditions were 5% acetonitrile in 95% 0.1 M TEAA (Triethylamine-Acetic Acid) buffer up to 100% acetonitrile over 30 min. Spectra were processed using the Empower 3 software.

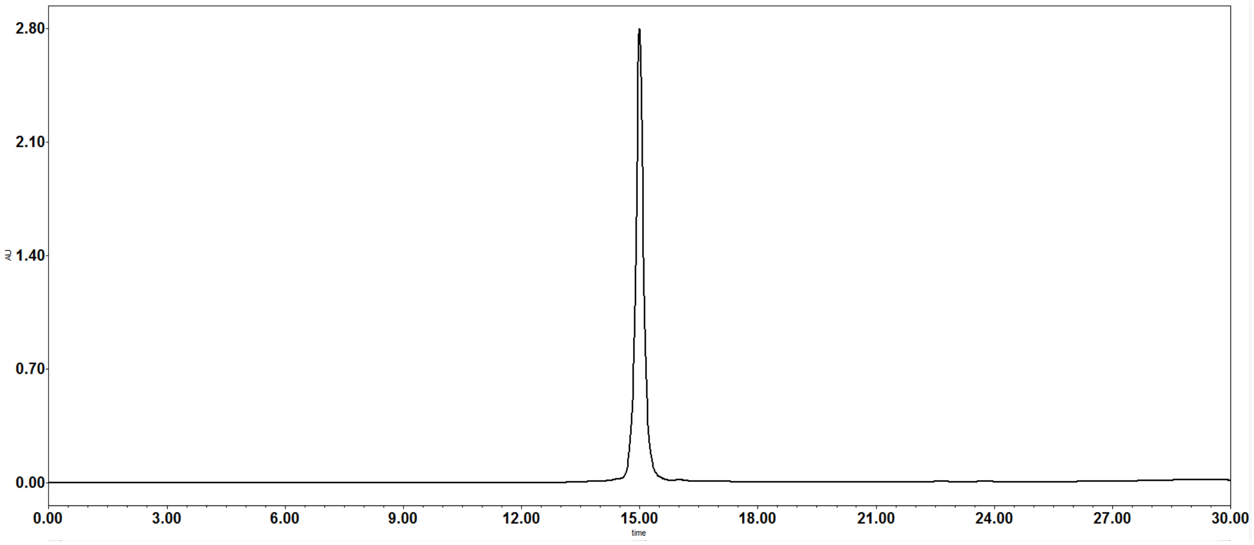

Figure S-27. HPLC chromatogram of sense strand LWTFG-21. Conditions were 5% acetonitrile in 95% 0.1 M TEAA (Triethylamine-Acetic Acid) buffer up to 100% acetonitrile over 30 min. Spectra were processed using the Empower 3 software.

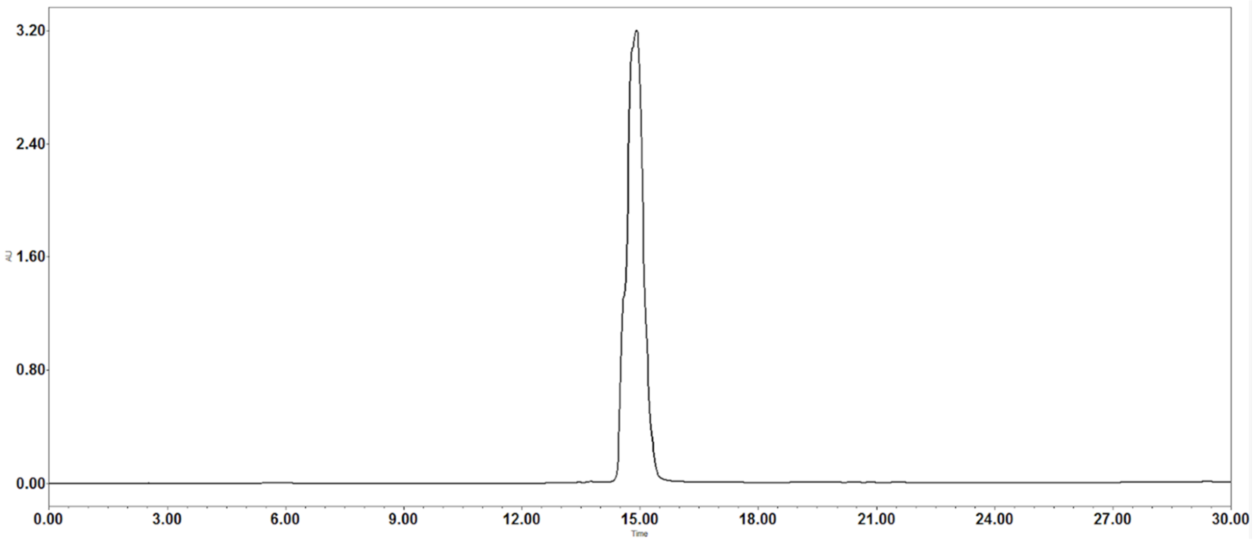

Figure S-28. HPLC chromatogram of sense strand PS-10-LWTFA-23. Conditions were 5% acetonitrile in 95% 0.1 M TEAA (Triethylamine-Acetic Acid) buffer up to 100% acetonitrile over 30 min. Spectra were processed using the Empower 3 software.

***HPLC chromatogram of all other RNA sense and antisense strands can be found in the supplement of our previous publication: "**Desaulniers, J-P.; Hammill, M.L; Giorgees, I.; Chiu, V.W-N.; Pendergraff, H.; Aguti, S.; Voutila, J.; Hansen, H.; Habib, N.; Lomonosova, Y.; Koch, T. Exploring Chemically-Modified Short Activating RNAs to Increase Nuclease Stability and Gene Activation. *J Med Chem*. **2025,** Vol. 68 Issue 21 Pages 22650-22664."

Table 1. Table of chemically modified RNA sequences and observed masses[a]

Antisense	Sequence	Expected Mass	Observed Mass
MEFA-2	3’-$U_MU_MG_MC_MU_MA_FA_FC_MC_MA_FA_FA_FG_MA_FG_MG_MU_MG_MU_MU_MG_M$-5’	6959.4	6959.1
MEFC-2	3’-$U_MU_MG_MC_FU_MA_MA_MC_FC_FA_MA_MA_MG_MA_MG_MG_MU_MG_MU_MU_MG_M$-5’	6995.5	6995.1
LWTFU-21	3’-U_MU_MMGCU_FAACCAAAGAGGU_FGU_FU_FG-5’	6781.9	6781.3
LWTFG-21	3’-$U_MU_MG_F$CUAACCAAAG_FAG_FG_FUG_FUUG_F-5’	6783.9	6783.3
PS-10-LWTFA-23	3’-$U_{M*}U_{M*}G_{*}C_{*}U_{*}A_{F*}A_{F*}C_{*}C_{*}A_{F*}A_FA_FGA_L$GGUGUUG-5’	6943.7	6943.3

[a] Subscript indicates chemical modifications to the monomer: M= 2’-OMe, F= 2’-Fluoro, L= LNA, U= UNA, *= phosphorothioate linkage. The top strand corresponds to the sense strand; the bottom strand corresponds to the antisense strand.

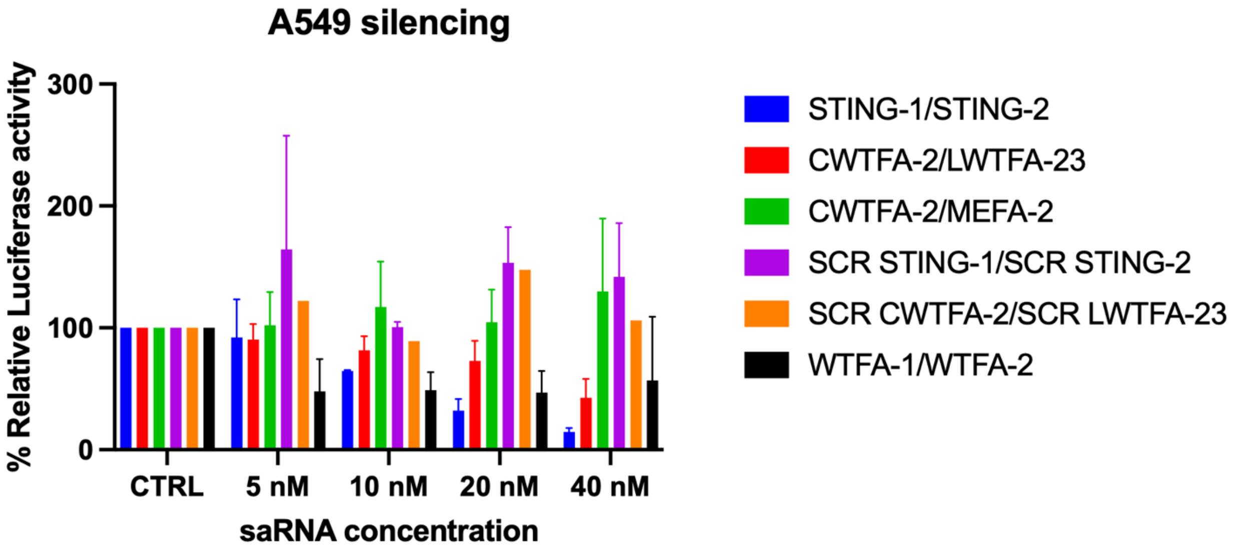

Figure S-29. Gene silencing data of *STING* by RNA duplexes CWTFA-2 annealed to MEFA-2 and LWTFA-23, and duplexes WTFA-1/WTFA-2, STING-1/STING-2, SCR STING-1/SCR STING-2 and SCR CWTFA-2/SCR LWTFA-23 at various concentrations (nM), monitored 48 hours post-transfection in A549 cells. CTRL indicates the untreated control, set to 100% luciferase expression. The sense strand CWTFA-2 with 2’-fluorinated adenosines and central C_3 propyl linker was annealed to two different antisense strands with different chemical modification designs, including 2’ fluorinated adenosines, and locked nucleic acid (LNA), and 2’-O-methylated bases. For WTFA-1/WTFA-2 the sense strand with 2’ fluorinated adenosines was annealed to an antisense strand with 2’ fluorinated adenosines. Firefly luciferase activity was normalized to *Renilla* luciferase. Mean with error bars that represent standard deviation of minimum two independent biological replicates for all duplexes except for SCR CWTFA-2/SCR LWTFA-23, where n=1.

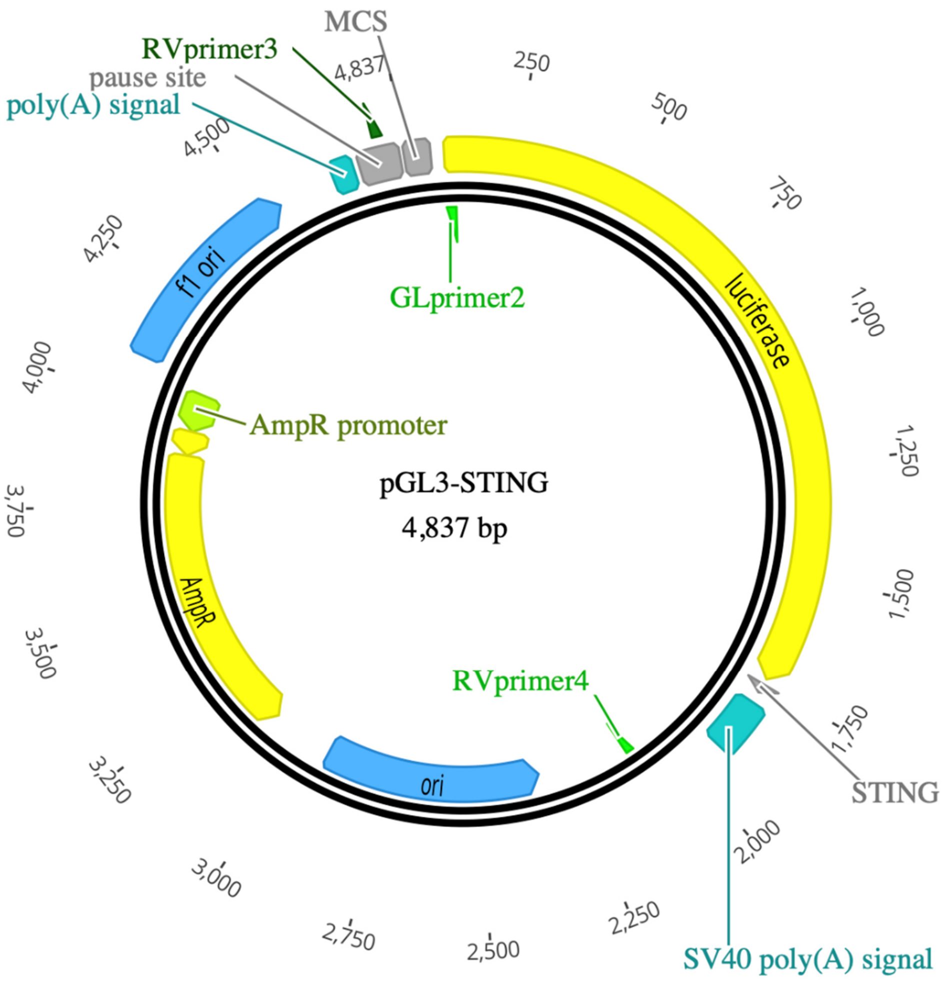

Figure S-30. Plasmid map of pGL3-STING. *STING* sequence inserted in the 3' UTR region of luciferase gene.

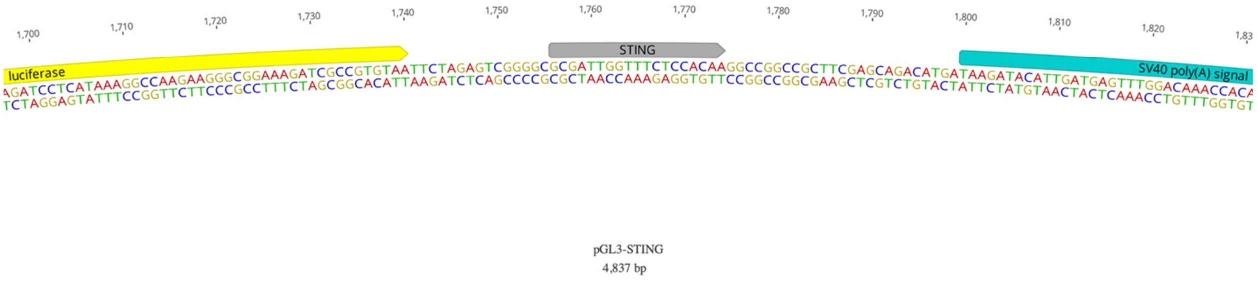

Figure S-31. Enlarged view of pGL3-STING insert region in the pGL3-STING plasmid map, in the 3' UTR region of the luciferase gene.

pGL3 GCAAGAAAAATCAGAGAGATCCTCATAAAGGCCAAGAAGGGCGGAAAGATCGCCGTGTAA 1740
reverse1 gcaagaaaaatcagagagatcctcataaaggccaagaagggcggaaagatcgccgtgtaa

pGL3 TTCTAGAGTCGGGGC-------------------GGCCGGCCGCTTCGAGCAGACATGAT 1781
reverse1 ttctagagtcggggcgcgattggtttctccacaacgccggccgcttcgagcagacatgat

pGL3 AAGATACATTGATGAGTTTGGACAAACCACAACTAGAATGCAGTGAAAAAAATGCTTTAT 1841
reverse1 aagatacattgatgagtttggacaaaccacaactagaatgcagtgaaaaaaatgctttat

Figure S-32. pGL3-*STING* plasmid sequencing result of *STING* insert location in the 3' UTR of the luciferase gene (highlighted in red).
